# Cocoa polyphenols and fiber modify colonic gene expression in rats

**DOI:** 10.1007/s00394-016-1230-0

**Published:** 2016-06-02

**Authors:** Malen Massot-Cladera, Àngels Franch, Margarida Castell, Francisco J. Pérez-Cano

**Affiliations:** 1 Department of Physiology, Faculty of Pharmacy, University of Barcelona, Av Joan XXIII s/n, Edifici B, 3a planta, 08028 Barcelona, Spain; 2 Nutrition and Food Safety Research Institute (INSA), University of Barcelona, Prat de la Riba, 171, Santa Coloma de Gramenet, 08921 Spain

**Keywords:** Cocoa, Colonic gene expression, Dietary fiber, Immune function, Lipid metabolism

## Abstract

**Purpose:**

Cocoa intake has been associated with health benefits, improving cardiovascular function and metabolism, as well as modulating intestinal immune function. The aim of this study was to take an in-depth look into the mechanisms affected by the cocoa intake by evaluating the colonic gene expression after nutritional intervention, and to ascertain the role of the fiber of cocoa in these effects.

**Methods:**

To achieve this, Wistar rats were fed for 3 weeks with either a reference diet, a diet containing 10 % cocoa (C10), a diet based on cocoa fiber (CF) or a diet containing inulin (I). At the end of the study, colon was excised to obtain the RNA to evaluate the differential gene expression by microarray. Results were validated by RT-PCR.

**Results:**

The C10 group was the group with most changes in colonic gene expression, most of them down-regulated but a few in common with the CF diet. The C10 diet significantly up-regulated the expression of *Scgb1a1* and *Scnn1* *g* and down-regulated *Tac4*, *Mcpt2*, *Fcer1a* and *Fabp1* by twofold, most of them related to lipid metabolism and immune function. The CF and I diets down-regulated the expression of *Serpina10* and *Apoa4* by twofold. Similar patterns of expression were found by PCR.

**Conclusion:**

Most of the effects attributed to cocoa consumption on genes related to the immune system (B cell and mast cell functionality) and lipid metabolism in the colon tissue were due not only to its fiber content, but also to the possible contribution of polyphenols and other compounds.

**Electronic supplementary material:**

The online version of this article (doi:10.1007/s00394-016-1230-0) contains supplementary material, which is available to authorized users.

## Introduction

The increasing interest in finding food-based strategies with health-improving effects has turned cocoa, the fruit of the cocoa tree (*Theobroma cacao* L.), into one of the subject matters of study. The health benefits provided by cocoa consumption in the prevention of diseases and physiological disorders have been described. Thus, cocoa consumption has long been demonstrated to improve cardiovascular status, decreasing blood pressure and reducing platelet aggregation [1, 2]. In addition, cocoa intake has been shown to increase HDL cholesterol, to protect against LDL oxidation and to improve hyperlipidemia, insulin sensitivity and hyperglycemia [3, 4], and there is emerging evidence of the beneficial role of chocolate in reducing body weight/body fat [5, 6]. In addition, studies concerning the cocoa protector effect against carcinogenesis [7], neurodegenerative diseases and cognitive function [8] have also been published.

Preclinical studies have demonstrated that cocoa exerts immune modulation in both systemic and intestinal compartments [9]. In this context, immunoglobulin production is modulated after cocoa intake and, in consequence, cocoa has been tested as a possible adjuvant treatment in allergic reactions [10]. In addition, the intake of cocoa affects the intestinal compartment by modulating the microbiota, as assessed in vivo and clinical studies [11] and also the Toll-like receptor (TLR) expression, key molecules involved in the recognition of microorganisms and inductors of inflammatory response [12] both in the inductive and effectors intestinal immune tissues, where antigens are sampled lymphocytes are activated and where the effector cells contribute to the formation of S-IgA antibodies, respectively [13–15].

Whatever the cocoa effect, most of these beneficial actions of cocoa have been attributed to its high content of polyphenols, mainly flavonoids such as (−)-epicatechin, (+)-catechin and their polymeric forms called procyanidins [16]. Thus, flavonoids have been related to cocoa-induced cardiovascular effects [1, 2], protection against carcinogenesis [7], and cognitive function [8]. Nevertheless, it must be added that cocoa, besides being a strong source of flavonoids, also has a high content of dietary fiber (26–40 %, mostly insoluble fiber) which could strengthen or be in part responsible for the effects described above. In general, dietary fiber appears to be particularly beneficial against various Western diseases, including metabolic syndrome [17] and, in particular, cocoa fiber, can prevent obesity by reducing body weight and improving glucose levels as well as controlling the blood lipid profile and lipid peroxidation in animals and human interventions [18].

One of the several possible mechanisms involved in the body weight control might be through the cocoa fiber’s capacity to increase fat elimination in feces [19, 20] by binding bile acids and dietary fats, and interfering with lipid absorption in the small intestine [21, 22]. In addition, the effects of cocoa fiber assisting in the control of arterial blood pressure in hypercholesterolemic subjects have been reviewed [20]. Protection against disorders related to the gastrointestinal tract (i.e., constipation, irritable bowel syndrome) or colorectal and other types of cancer has also been attributed to cocoa fiber fermentation by the colonic microbiota which leads to the formation of short chain fatty acids (SCFA) [23].

Taking the above evidences together, the influence of cocoa consumption on the systemic and intestinal compartments is clear. These effects can be attributed to both its polyphenols and fiber content which can reach the colon, but also to their microbial metabolites produced after cocoa consumption. Therefore, we hypothesize that important events after cocoa intake are produced in the colon environment, where bioactive compounds of cocoa would interfere then in the gene expression of colon cells. The objective of the present work was to study in-depth the mechanisms involved in the beneficial effects of cocoa, mainly focused on immune system and lipid metabolism modulation, by evaluating changes in colonic gene expression after a preclinical nutritional intervention, and to ascertain whether cocoa fiber is responsible for these effects. For this purpose, the technique chosen is microarray analysis due to the fact that it is a powerful tool that allows global analysis of the expression of thousands of genes to be performed in a single assay [24].

## Materials and methods

### Animals and diets

Female Wistar rats (3 weeks old) were obtained from Janvier (Saint-Berthevin, France) and housed in cages under conditions of controlled temperature and humidity in a 12:12 light–dark cycle. The rats were randomly distributed into four dietary groups (*n* = 10/each). The reference group (REF) was fed with a standard diet AIN-93 M (Harlan, Barcelona, Spain); the cocoa group (C10) was fed with a diet containing 0.4 % of polyphenols, 0.85 % of soluble fiber and 2.55 % of insoluble fiber provided by 10 % cocoa; the cocoa fiber group (CF) received a diet with the same cocoa soluble and insoluble fiber proportion as the C10 group but with a very low amount of polyphenols (<0.02 %); and a fourth group (I) received the same amount of soluble fiber as the C10 group (0.85 %) but in the form of inulin in order to distinguish the particular effect of cocoa fiber (Table 1). Natural Forastero cocoa and cocoa fiber powders [Idilia Foods SL (formerly Nutrexpa SL), Barcelona, Spain] with 4.02 and 0.35 % of polyphenols, respectively, were used to elaborate the C10 and CF diets. Inulin from chicory roots (Fibruline^®^ Instant; InnovaFood 2005, S.L, Barcelona, Spain) was used as a reference soluble fiber. The three experimental diets were elaborated from AIN-93 M formula by subtracting the amount of carbohydrates, proteins, lipids and insoluble fibers provided by the corresponding cocoa or cocoa fiber extracts. The resulting chows were isoenergetic and had the same proportion of macronutrients (carbohydrates, proteins and lipids) and insoluble fiber as the reference diet (Table 1).

**Table 1:** Composition of nutrients and polyphenols provided by the three experimental diets (g/kg diet)

Components	REF (g/kg) AIN-93 M	C10 (g/kg)	CF (g/kg)	I (g/kg)
Casein	121.5	97.1	109.7	118.7
L-Cystine	1.8	1.4	1.4	1.6
Corn starch	418.1	423.7	437.2	426.4
Maltodextrin	148.5	118.7	120.4	131.5
Sucrose	102.6	108.7	110.9	110.9
Soybean oil	38.2	26.2	33.5	38.9
Cellulose	50	24.5	26.5	50.0
Minerals	35.3	27.7	27.9	31.3
Vitamins	9.1	7.2	7.2	8.1
Choline bitartrate	2.5	2.0	2.0	2.2
*tert*-Butylhydroquinone	0.008	0.006	0.006	0.006
Water	72.4	63	71.1	72
Cocoa powder	–	100	–	–
Protein	–	22	–	–
Carbohydrate	–	16	–	–
Lipid	–	11	–	–
Fiber (insoluble/soluble)	–	34 (25.5/8.5)	–	–
Minerals	–	6	–	–
Total polyphenols^1^	–	4	–	–
Water	–	7	–	–
Cocoa fiber powder	–	–	52.3	–
Protein	–	–	8	–
Carbohydrate	–	–	0.5	–
Lipid	–	–	4.8	–
Fiber (insoluble/soluble)	–	–	31.9 (23.4/8.5)	–
Minerals	–	–	7	–
Total polyphenols^1^	–	–	0.2	–
Water	–	–	–	–
Inulin powder	–	–	–	8.5
Protein	–	–	–	–
Carbohydrate	–	–	–	–
Lipid	–	–	–	–
Fiber (insoluble/soluble)	–	–	–	8.5 (−/8.5)
Minerals	–	–	–	–
Total polyphenols^1^	–	–	–	–
Water	–	–	–	–

^1^Total polyphenol compounds were quantified by Folin–Ciocalteu method

Animals were given free access to water and chow. The diets lasted for 3 weeks. Studies were performed according to the criteria outlined by the Guide for the Care and Use of Laboratory Animals. Experimental procedures were reviewed and approved by the Ethical Committee for Animal Experimentation of the University of Barcelona (ref. 358/12).

### Sample collection and preparation

At the end of the 3-week nutritional intervention, the rats were anesthetized intramuscularly with ketamine (90 mg/kg) (Merial Laboratorios, S.A., Barcelona, Spain) and xylazine (10 mg/kg) (Bayer A.G, Leverkusen, Germany). A 5-mm piece of the middle of the colon was collected from all animals in aseptic conditions in RNAlater^®^ and kept at 4 °C overnight before storing at −20 °C for further gene expression analysis by microarray.

### RNA extraction

For RNA isolation, tissue samples were transferred into lysing matrix tubes (MP Biomedicals, Illkirch, France) containing an appropriate buffer and were homogenized in a FastPrep^®^ instrument (MP Biomedicals) for 30 s. Lysates were centrifuged for 3 min at 12,000 *g* to eliminate excess tissue debris. The RNA was isolated by the RNeasy Mini Kit (Qiagen, Madrid, Spain) following the manufacturer’s recommendations and then quantified with a NanoDrop spectrophotometer and NanoDrop IVD-1000 v.3.1.2 software (NanoDrop Technologies, Wilmington, DE, USA). The Agilent 2100 Bioanalyzer with the RNA 6000 LabChip kit (Agilent Technologies, Madrid, Spain) was used to provide an RNA integrity number for each sample. All samples used for further experiments showed an RNA integrity number (RIN) ≥ 9 and purity between 1.885 and 2.042 using the A_260_/A_280_ ratio.

### Microarray procedure

The study of the differential expression profiling was carried out with a SurePrint-G3 Rat GE 8 × 60 K microarray kit (ID 028279, Agilent Technologies, Madrid, Spain), following a *loop* experimental design in which a pairwise comparison was done. Quadruplicate samples for each experimental condition were employed, and dye swaps (Cy3 and Cy5) were performed to the RNA amplified from each sample. RNA quality was assessed using a TapeStation (Agilent Technologies). RNA concentration and dye incorporation were measured using a UV–Vis spectrophotometer (Nanodrop 1000, Agilent Technologies, Wilmington, DE, USA). Labeling and hybridization to microarray were conducted following the manufacturer’s two-color protocol (*Two*-*Color Microarray*-*Based Gene Expression Analysis v. 6.5, Agilent Technologies*), using *LowInput QuickAmp Labeling Kit* and *Agilent Microarray Hybridization Chamber Kit* for labeling and hybridization, respectively. Microarray chips were then washed and immediately scanned using a DNA Microarray Scanner (Model G2505C, Agilent Technologies) by the Genetic Diagnostic Bioarray facilities (Bioarray, Alicante, Spain).

### Microarray data analysis

Data extraction was performed with Agilent Feature Extraction Software v.10.7 (Agilent Technologies). Bioinformatic analysis was performed with Bioconductor software under R environment, using the following packages: *limma* (v.3.16.1) for background correction and normalization, *Marray* and *pcaMethods* for quality control plots, *RankProd* for differential expression and finally *GOstats* (v.2.26.0.) and *GSEABase* for gene ontology functional analysis. Latest gene annotations available were used. Raw feature intensities were background-corrected using *normexp* background correction algorithm. Within-array normalization was done using spatial and intensity-dependent *loess*. *Aquantile* normalization was used to normalize between arrays. The expression of each gene is reported as the base 2 logarithm of ratio of the value obtained of each condition relative to control condition (REF group). A gene is considered differentially expressed if it displays a PFP (percentage of false prediction, equivalent of false discovery rate, FDR) less than 0.05 by rank product nonparametric method (RankProd). Venn diagrams in GX allowed finding differently expressed genes that followed the same pattern (e.g., up-regulated or down-regulated) in common among the experimental conditions. Finally, up- and down-regulated genes were analyzed in terms of gene ontology by using a hypergeometric analysis (GOStats). The output of this analysis was then filtered using two different criteria. On the one hand, data were filtered by fold expression, generating lists of differentially expressed genes by at least twofold, whereas on the other hand, data were filtered taking into account the statistical significance of the enrichment analysis for the over and under represented GO terms belonging to the biological process (BP), cellular component (CC) and molecular function (MF) domains. The microarray data have been lodged in the Gene Expression Omnibus (http://www.ncbi.nlm.nih.gov/geo/) as accession number: GSE7095.

### Gene function analysis

Gene ontology annotation of broad terms belonging to the BP domain was performed using ClueGO v2.2.4 and Clupedia v1.2.4, a Cytoscape plugins [25, 26]. Briefly, gene symbols from both up- and down-regulated genes (Tables 2, 3, respectively) were uploaded and analyzed with the following default parameters: enrichment (right-sided hypergeometric test) correction method using Bonferroni step down analysis mode and showing only the pathways with pV ≤ 0.05; “Function” load a gene cluster list for *Rattus norvegicus*; view style setting set to “Cluster”; evidenced codes set to “All”; networking specificity set to “medium” (GO levels 3–8); advanced term/pathway selection option set to 3 for the minimum genes and 4 % of genes for both clusters; and Kappa score threshold set to 0.3. A network was constructed for each pairwise comparison (C10 *vs* REF; CF *vs* REF and I *vs* REF; 3 networks in total). These networks provide a global view of potentially relevant, interacting partners of genes with abundant changes.

**Table 2:** List of up-regulated genes differentially expressed by ≥twofold with a *P* < 0.05 obtained after the 10 % cocoa diet intake (C10 group) compared to the REF group (*n* = 4/group)

Symbol	C10 *vs* REF	CF *vs* REF	I *vs* REF
	*log*2 FC	*log*2 FC	*log*2 FC
*Scgb1a1*	3.948	1.716	1.139
*Scnn1g*	3.119	1.133	0.573
*Fxyd4*	2.583	2.089	0.718
*Slc15a1*	2.332	1.388	0.475
*Gbp1*	2.207	1.662	0.432
*Cyp2f4*	2.185	1.436	0.367
*Dpysl4*	2.069	1.387	***0.242***
*Fmo2*	2.040	1.007	0.426
*Pdyn*	1.873	***0.194***	***0.175***
*Mt2A*	1.656	0.644	−0.147
*RGD1561239*	1.597	1.787	0.742
*Sftpd*	1.583	0.163	0.351
*Mme*	1.580	0.636	0.353
*Hoxd13*	1.575	0.615	0.100
*Chst5*	1.533	0.591	0.082
*Atp12a*	1.457	1.630	0.500
*Thbs4*	1.450	0.908	0.302
*Hoxd10*	1.403	0.668	***0.164***
*Trpv1*	1.390	0.749	***0.058***
*Hoxb13*	1.358	0.786	0.101
*Spink3*	1.338	1.162	0.463
*LOC687303*	1.327	0.734	0.755
*Abat*	1.319	1.272	0.477
*Angptl4*	1.316	0.914	0.670
*Aox4*	1.311	***0.054***	***0.320***
*B3gnt7*	1.265	0.403	0.019
*Pcsk9*	1.253	0.048	0.063
*RGD1560608*	1.225	0.759	0.709
*Guca2b*	1.211	0.811	***0.287***
*Padi3*	1.190	0.832	0.462
*Scnn1b*	1.181	0.499	***0.057***
*LOC287167*	1.178	0.607	0.296
*RGD1308274*	1.142	***0.073***	0.321
*Smc1b*	1.133	0.968	***0.031***
*Pde7a*	1.129	0.708	0.405
*LOC100363350*	1.126	0.621	0.446
*Gsta2*	1.111	0.537	***0.157***
*Slc20a1*	1.110	0.624	***0.056***
*RGD1310110*	1.097	0.410	***0.231***
*LOC689064*	1.095	1.024	0.501
*Rnase1*	1.094	1.063	1.218
*Dnase1*	1.088	1.087	***0.124***
*siat7D*	1.058	0.363	***0.096***
*Serpinc1*	1.053	0.873	0.469
*LOC494499*	1.029	0.576	***0.276***
*Fbxl13*	1.028	1.009	***0.067***
*Alox15*	1.028	0.387	***0.191***
*Creg1*	1.021	0.406	**−** ***0.008***
*Rnase1l2*	1.010	0.857	**1.012**
*Alas2*	1.007	0.997	0.452

This includes the gene symbol for all the up-regulated genes. Values are expressed as a *log*2 of fold change and shown when ≥1. Values for the two other experimental diets are also included, although independently of their fold change and/or their significance. Values in bold and cursive represent the non-statistically significant changes

**Table 3:** List of down-regulated genes differentially expressed by ≥twofold with a *P* < 0.05 obtained after the 10 % cocoa diet intake (C10 group) compared to the REF group (*n* = 4/group)

Symbol	C10 *vs* REF	CF *vs* REF	I *vs* REF	Symbol	C10 *vs* REF	CF *vs* REF	I *vs* REF
	*log*2 FC	*log*2 FC	*log*2 FC		*log*2 FC	*log*2 FC	*log*2 FC
*Syce2*	−1.000	−1.117	***0.028***	*Olfm4*	−1.291	−0.826	***0.002***
*Chga*	−1.011	−0.328	−0.133	*Mt3*	−1.298	−0.631	−0.372
*Smpx*	−1.016	−0.349	−0.381	*Casq2*	−1.298	−0.643	**−** ***0.218***
*Myl2*	−1.019	−0.940	−0.823	*Hsd3b5*	−1.302	−0.648	−0.340
*Ptgis*	−1.026	−0.370	**−** ***0.173***	*Gsta5*	−1.308	−0.560	**−** ***0.158***
*Cfd*	−1.037	**−** ***0.087***	0.009	*Pln*	−1.309	−0.384	−0.349
*Msln*	−1.041	0.382	0.377	*Nox1*	−1.313	−0.256	0.207
*LOC360228*	−1.041	0.559	0.231	*Me1*	−1.324	−0.443	**−** ***0.264***
*Kcnj5*	−1.043	−1.062	−0.766	*Casc4*	−1.334	−0.688	−0.715
*Car3*	−1.045	0.278	−0.263	*LOC682360*	−1.335	−0.459	−0.314
*Slpil2*	−1.050	***0.102***	0.425	*Sln*	−1.360	−0.649	−0.410
*Ca2*	−1.055	−0.551	−0.087	*RGD1563231*	−1.370	−0.170	−0.580
*Rgs2*	−1.059	−0.856	−0.760	*Slpi*	−1.378	***0.188***	0.552
*Mfsd2a*	−1.059	−0.455	**−** ***0.262***	*RGD1562127*	−1.382	−0.687	−0.360
*Tnfrsf12a*	−1.059	−0.600	−1.353	*Sgcg*	−1.386	−0.884	−0.543
*Car8*	−1.060	−0.525	−0.339	*LOC685106*	−1.390	0.215	−0.613
*Mybpc2*	−1.064	−0.459	**−** ***0.353***	*Hdc*	−1.393	**−** ***0.281***	**−** ***0.076***
*Gcnt3*	−1.071	−0.549	−0.183	*Retnlb*	−1.420	1.223	1.939
*Mrap*	−1.072	−0.045	−0.363	*RGD1565374*	−1.430	−0.901	−0.378
*Tmprss9*	−1.077	−1.024	−0.582	*Apobec2*	−1.436	−0.613	**−** ***0.243***
*Gldn*	−1.085	−0.522	**−** ***0.161***	*Sypl2*	−1.457	−0.801	−0.867
*Hsd3b6*	−1.086	−0.518	−0.228	*Fcer1a*	−1.497	**−** ***0.248***	**−** ***0.283***
*Casq1*	−1.086	−0.436	**−** ***0.282***	*Hsd17b6*	−1.503	−0.988	−0.325
*RGD1560314*	−1.089	−0.959	**−** ***0.119***	*RGD1565970*	−1.509	−0.198	0.413
*RGD1309651*	−1.094	−0.426	**−** ***0.103***	*Tph1*	−1.518	−0.642	−0.305
*Ly49i2*	−1.102	−0.604	−1.177	*Cfb*	−1.551	0.167	0.560
*LOC363060*	−1.108	−0.829	−0.616	*Slfn3*	−1.558	***0.166***	0.249
*LOC688635*	−1.113	−0.189	**−** ***0.038***	*Retnlg*	−1.560	**−** ***0.018***	0.509
*Kif1c*	−1.125	−0.943	−0.701	*LOC679045*	−1.562	−0.216	−0.234
*LOC687842*	−1.126	−0.830	−0.489	*Cma1*	−1.583	**−** ***0.288***	0.256
*Scx*	−1.131	−0.368	−0.413	*Cpa3*	−1.609	−0.258	**−** ***0.115***
*Slc13a1*	−1.134	−0.585	−0.301	*Retnla*	−1.627	0.878	1.162
*Ccr10*	−1.137	***0.036***	0.419	*Cyp4f1*	−1.634	−0.582	0.169
*Mzb1*	−1.140	0.064	0.772	*Npc1l1*	−1.662	−1.294	−0.707
*Ms4a2*	−1.144	**−** ***0.127***	***0.012***	*Tff1*	−1.663	−0.786	−0.310
*Rup2*	−1.145	−1.201	−0.220	*Atf3*	−1.681	−1.015	−1.715
*Rrad*	−1.150	−0.320	**−** ***0.193***	*Fabp1*	−1.682	−0.780	−0.074
*Fbln7*	−1.156	−0.540	−0.398	*Tnfrsf17*	−1.690	**−** ***0.139***	0.572
*Bpifb6*	−1.156	−0.825	−0.560	*Spink4*	−1.714	−2.376	−1.237
*Cd55*	−1.162	−0.438	−0.237	*LOC100360169*	−1.718	0.232	1.101
*F2*	−1.165	−0.640	−0.315	*Ros1*	−1.733	−1.914	−1.672
*Fabp4*	−1.170	−0.233	−0.334	*Thrsp*	−1.747	−0.067	−0.380
*Myom2*	−1.171	−0.772	−0.257	*RatNP* − *3b*	−1.761	−2.005	−0.576
*RGD1305928*	−1.180	−0.851	−0.388	*RGD1564563*	−1.793	−0.383	**−** ***0.092***
*Slc18a1*	−1.184	−0.791	−0.599	*Mcpt8*	−1.824	−0.324	0.315
*Rgs1*	−1.184	−0.675	−0.604	*Mcpt8l3*	−1.824	−0.462	***0.151***
*Dapl1*	−1.196	−0.753	−0.375	*Adipoq*	−1.826	0.053	−0.265
*Tmem22*	−1.199	−0.583	−0.419	*Mcpt9*	−1.834	−0.384	***0.240***
*Ccl12*	−1.212	0.206	0.243	*Mcpt4*	−1.952	0.233	0.296
*Siglec1*	−1.213	***0.130***	***0.102***	*Mcpt1*	−1.966	**−** ***0.014***	0.285
*Fcgr3a*	−1.230	**−** ***0.225***	***0.136***	*Mcpt10*	−1.985	−0.523	***0.192***
*LOC686141*	−1.230	−0.200	0.140	*LOC100359793*	−1.987	−0.141	1.137
*LOC683753*	−1.232	−0.795	**−** ***0.121***	*Scd1*	−1.998	0.138	−0.400
*Bhlha15*	−1.233	−0.496	−0.067	*Mcpt8l2*	−2.003	−0.532	***0.196***
*LOC688507*	−1.238	−0.184	0.110	*Pga5*	−2.086	−1.394	−0.775
*H19*	−1.242	**−** ***0.227***	−0.317	*Gkn2*	−2.099	−1.000	−1.584
*Cxcl11*	−1.260	−0.859	−0.267	*Mcpt1l4*	−2.170	−0.112	0.239
*Rgs4*	−1.262	−0.570	−0.720	*RGD1562035*	−2.179	−0.248	***0.176***
*Ces2c*	−1.269	−0.944	−0.445	*Pzp*	−2.223	−1.221	−1.117
*Fut9*	−1.270	−0.997	−0.334	*Fcrls*	−2.342	−0.529	0.208
*Srpk3*	−1.272	**−** ***0.242***	**−** ***0.042***	*Bpifb2*	−2.508	−1.532	−1.300
*RGD1560949*	−1.273	−1.471	−1.135	*Mcpt2*	−2.545	−0.290	***0.255***
*Npw*	−1.276	−0.777	−0.406	*Tac4*	−2.766	−1.731	−1.279
*Rbp4*	−1.283	**−** ***0.026***	−0.205				

This includes the gene symbol for all the down-regulated genes. Values are expressed as a *log*2 of fold change and shown when ≤−1. Values for the two others experimental diets are also included, although independently of their fold change and/or their significance. Values in bold and cursive represent the non-statistically significant changes

### Validation of gene expression by real-time PCR

Two micrograms of total RNA was converted to cDNA. Specific PCR TaqMan^®^ primers and probes (Applied Biosystems, AB, Weiterstadt, Germany) were used to measure selected targets: *Scgb1a1* (Rn00564903_m1, inventoried (I)), *Scnn1* *g* (Rn00566891_m1, I), *Fabp1* (Rn00664587_m1, I), *Serpina10* (Rn00592428_m1, I), *Mcpt2* (Rn00756479_g1, I), *Fcer1a* (Rn00562369_m1, I), *Apoa4* (Rn00562482_m1, I) and *Tac4* (Rn00597278_m1, I). Quantitative real-time PCR assays were performed in duplicate for each sample using an ABI PRISM 7900HT Sequence Detection System (AB). Quantification of the genes being studied was normalized to the housekeeping genes *Gusb* (Rn00566655_m1, I). The SDS v2.4 software (AB) was used to analyze the expression data. Results are expressed as the fold change of the amount of target mRNA relative to the endogenous control expression calculated using the standard 2^−∆∆Ct^ method for the three experimental groups relative to values from the REF group, which represents a onefold change in gene expression.

### Statistics

Statistical analysis of PCR results was performed using the software package SPSS 22.0 (SPSS, Inc.). Levene’s and Kolmogorov–Smirnov tests were applied to assess variance equality and normal distribution, respectively. Nonparametric tests were performed when normal distribution and equality of variance did not exist. Specifically, Kruskal–Wallis and Mann–Whitney *U* tests were used in order to assess significance for independent samples. Significant differences were established at *P* < 0.05.

## Results

### Body weight and chow intake

Body weight and chow intake were monitored throughout the study. The initial body weight was similar among the groups (44.4 ± 0.7 g) increasing during the study up to 154.8 g in the REF group. In animals fed 10 % cocoa diet, body weight increase was lower than that in the other groups, the difference already being significant at day 7, and, at the end, the body weight was ~35 g less than that in the rest of the groups (*P* < 0.05). This effect, also observed in previous studies [13, 14, 27], was not related to a lower chow intake since this was similar in all the experimental groups (data not shown) throughout the study as well as in reported studies [13, 14, 27]. No changes in body weight were found due to the CF or I diets.

### Colon gene expression profiles and commonly modulated genes

Three weeks of diet containing either 10 % cocoa, the equivalent amount of cocoa fiber or the equivalent amount of inulin as soluble fiber, produced changes in the gene expression of colon. The C10 group produced more changes than the CF and I groups, with most of these changes being down-regulated and only a few of them in common with the CF diet. Likewise, very few similarities were detected between the CF and I diets, the I group being the one with the fewest changes.

**Fig. 1:**
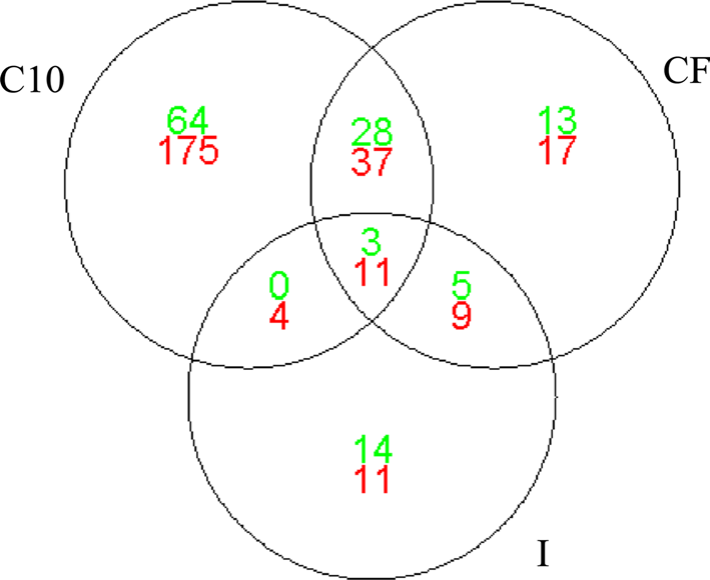
Differentially expressed genes among all three experimental groups versus REF group are visualized by means of a Venn diagram (*n* = 4/group). The *diagram* shows the number of genes that belongs to each of the individual lists, the genes in common between each pair of lists and the genes in common among all three lists (in the center of the representation) for each type of regulation (*Green* up-regulation and *Red* down-regulation) compared to the REF group

To show the number of genes affected in common, a Venn diagram representation was drawn comparing separately the list of significantly up- and down-modified genes (*P* < 0.05) obtained for the three experimental diet groups of animals compared to the REF group (Fig. 1). The C10 and CF groups shared a total of 79 modulated genes, 31 of which were up-regulated and 48 down-regulated. The CF and I groups were found to have a total of 28 modulated genes in common, of which 8 were up-regulated and 20 down-regulated. Finally, a total of 18 modulated genes—3 up-regulated and 15 down-regulated—were shared between the C10 and I groups. The expression of 14 genes was found to have been modified in common by the three experimental diets (3 up- and 11 down-regulated).

Focusing on the uncommon modulated genes (Fig. 1), the cocoa-fed animals showed the highest modulation for both up- and down-regulations (64 and 175, respectively), whereas both the cocoa fiber and inulin diets modulated a similar number of different genes, with the number of up- and down-regulated genes also being similar.

### Gene ontology (GO) annotation

We have considered the most significant GO terms belonging to the biological process (BP), cellular component (CC) and molecular function (MF) domains for up- and down-regulated genes. Criteria include both the *P* value together with the counts and the expected counts for each GO term in the C10 and the CF groups in comparison with the REF group (Tables S2 and S3, supplementary material). When those GO terms were also commonly enriched by the I diet, the number of the *P* value together with the counts and the expected counts were also included.

**Fig. 2:**
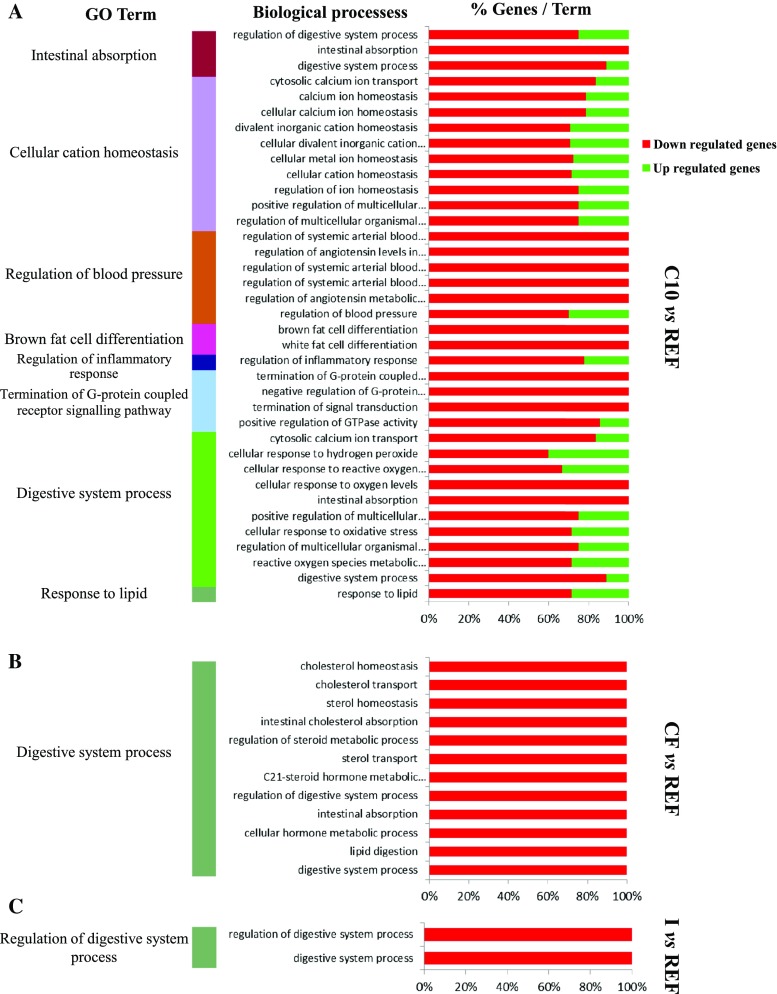
Common BP belonging to the most significantly overrepresented GO terms for the analyzed clusters. In *red* are shown the percentages of Cluster 1 (down-regulated) genes associated with the most significant (*P* < 0.05) overrepresented biological process GO terms and in *green* the percentages of Cluster 2 (up-regulated) genes associated with these most significant overrepresented biological process GO terms

A functional classification, according to the BP with the most significant (*P* < 0.05) terms, was determined for each pairwise comparison (C10 *vs* REF; CF *vs* REF; and I *vs* REF) together for both up- and down-regulated genes as detailed in material and method section (Fig. 2). The first one shows the overrepresented GO terms by the C10 diet compared to the REF one (Fig. 2a). It revealed 8 functional subgroups which mainly contain down-regulated genes. Among these subgroups, cellular cation homeostasis had the highest number of associated genes followed by digestive system process. All genes related to the regulation of blood pressure terms belonged to cluster 1 (down-regulated genes). The second comparison was between rats fed with the CF diet and those fed with the REF diet (Fig. 2b). When animals were fed the CF, only one functional subgroup (digestive system process) was obtained which only contains down-regulated genes. Similarly, a unique functional category (regulation of digestive system process) containing down-regulated genes appeared when the overrepresented in the up- and down-regulated genes by I diet were compared to the reference group (Fig. 2c).

### Analysis of biological processes

**Fig. 3:**
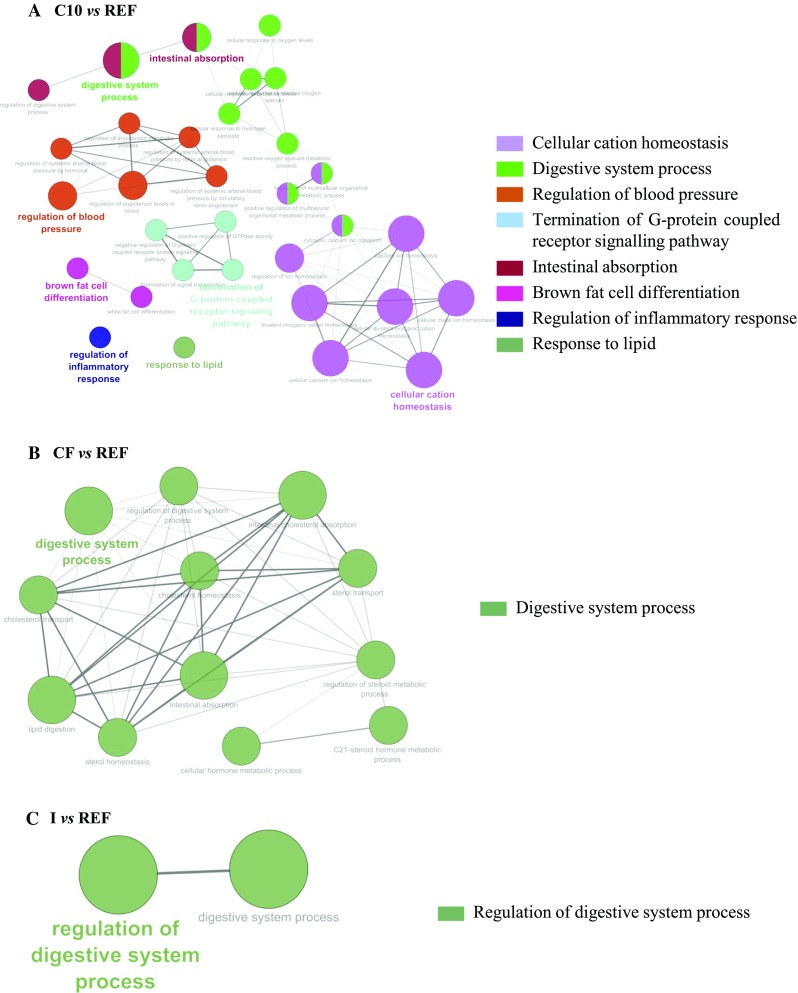
Biological processes network according to ClueGO (only overrepresented categories in down-regulated genes are found). Overrepresented BP GO terms are represented as nodes, and the nodes size represents the term enrichment significance by the Bonferroni step down method, linked by their Kappa score level (≥0.30). *Edges* represent connections between the nodes and the length of each edge reflects the relatedness of two processes. Only the labels of the most significant terms (*P* < 0.05) are shown

For each one of the comparison discussed above, ClueGO and CluePedia were used to create networks employing all the interactions found between them including the overrepresented BP GO terms in both up- and down-regulated genes (Fig. 3). The network obtained after comparing the C10 and the REF groups was the one with the highest complexity due to the interactions among the different BP. All these interactions were among overrepresented categories in down-regulated genes (Fig. 3a). The digestive system process had the most significant enrichment, followed by the cellular cation homeostasis term. Moreover, the digestive system process closely related to the intestinal absorption term.

When the CF and REF groups were compared (Fig. 3b), the obtained network revealed that the digestive system process category was the only significant GO biological process obtained. From the considered dataset, all biological processes belonging to this category presented similar significant enrichment. All the obtained interactions were among overrepresented categories in down-regulated genes.

The simplest network was obtained after comparing I and REF groups. It showed that the regulation of digestive system process was the only significant overrepresented category obtained in down-regulated genes.

No significant overrepresented GO terms for up-regulated genes were observed in any comparisons.

### Impact of cocoa diet on gene expression

An extensive number of genes were both up- and down-regulated after the C10 and CF diets (Table S1, supplementary material). However, given that the main aim of the present study was to elucidate the mechanism by means of which cocoa intake modulates the immune response and the lipid metabolism, the “Results” and “Discussion” sections of the present work are mainly focused in those modulated genes belonging to these two pathways.

The ≥twofold up- and down-regulated genes expression after cocoa diet intake (C10 group) compared to the REF group is shown in Tables 2 and 3, respectively. Likewise, the fold change expression of the same genes by the CF and I diets is included. The two genes with the highest fold up-regulation after C10 diet were *Scgb1a1* and *Scnn1* *g* (3.948 and 3.119 *log* fold change, respectively) (Table 2), the first one encoding a small epithelium-derived protein related to anti-inflammation, and the second one the gamma subunit of a membrane-bound ion channel which plays an essential role in electrolyte (basically sodium transport in kidney, colon, lung and sweat glands) and blood pressure homeostasis. If we consider GO terms, the most up-regulated genes in the C10 group (*Scgb1a1, Cyp2f4, Dpysl4, Fmo2, Hoxd10, Spink3, Angptl4, Padi3, Pde7a, Creg1 and Alas2*) (Table 2) belong to the metabolic process (including total, primary and cellular processes, GO:0008152, GO:0044238 and GO:0044237, respectively, in BP domain) and the binding pathways (GO:0005488 in MF domain) terms, respectively. Moreover, the most significantly enriched up-regulated GO terms are also represented here. Particularly, *Mt2A, Sftpd, Atp12a, Trpv1, Angptl4, Guca2b, Alox15 and Alas2* belong to the response to inorganic substance term (GO:0010035 in BP domain), whereas most of the significantly enriched up-regulated GO terms (GO:0019825, GO:0020037, GO:0046906, GO:0005506, GO:0022892) in the MF domain are represented by the *Cyp2f4*, *LOC287167*, *LOC689064*, *Alox15*, *Alas2, Scnn1* *g and Fxyd4*.

On the other hand, the most up-regulated genes after CF and I diet intake were *Fxyd4* (a protein-coding gene member of a family of small membrane proteins) and *Vom2r3* (a protein-coding gene involved in G protein-coupled receptor signaling pathway), respectively. The last gene did not undergo a twofold changed by 10 % cocoa diet (Table S1, supplementary material).

The gene with the highest down-regulation after the C10 group was *Tac4* (tachykinin 4), a member of the tachykinin family of neurotransmitter peptides involved in the immune system regulation, which decreased by 2.766 (Table 3). This gene and other genes down-regulated by 10 % cocoa diet are related to the immune system. Some others are the *Fcer1a* (*log*2 FC −1.497) encoding the expression of the high-affinity receptor I for IgE Fc fragment for alpha polypeptides, and *Ms4a2* (*log*2 FC −1.144) encoding the same receptor for beta polypeptides. Both genes, together with *Tac4*, are implicated in pathways related to the mast cell-mediated immunity (GO:0002448), its activation (GO:0045576, GO:0002279, GO:0033008, GO:0033005, GO:0033006, GO:0033003) and its degranulation (GO:0043303, GO:0043304, GO:0043306) in the BP domain. *Fcer1a* and *Ms4a2*, acting together with *Fcgr3a* (*log*2 FC −1.230), are also involved in the immunoglobulin binding pathway (GO:0019865 in the MF domain) and, together with *Adipoq* (*log*2 FC −1.826), are involved in cytokine production (GO:0001816 in the BP domain).

In addition, in C10 group a great number of down-regulated genes, such as *Adipoq*, *Fabp1*, *Fabp4*, *Fcer1a*, *Ptgis*, *Cyp4f1 and Npc1ǀ1*, affect the lipid and fatty acid metabolic process pathways (GO:0006629 and GO:0006631 in the BP domain, respectively). Many other genes down-regulated by 10 % cocoa diet are involved in the proteolysis pathway such as *Mcpt1, Mcpt2, Mcpt4, Mcpt8, Mcpt10 and Pga5* (GO:0006508 in the BP domain); response to stress such as *F2, Cd55, Nox1, Fcer1a, Tph1 and Tff1* (GO:0006950 in the BP domain); plasma membrane such as *Tmprss9, Tnfrsf12a, Casq1, Smpx and Fcgr3a*, (GO:0005886 in the CC domain); and protein binding such as *Retnlg, Fabp4, Npw, Myl2, and Bhlha15* (GO:0005515 in the MF domain).

Most of these genes highly modified by the cocoa diet were not or were only slightly modified in the CF and I groups with the exception of *Ros1* (an orphan receptor tyrosine kinase that plays a role in cell differentiation) and *Tac4*, which were also modified in the CF-fed animals, and *Atf3* (activating transcription factor 3) and *Retnlb* (a cysteine-rich cytokine expressed in the colon and implicated in immunity) in the I-fed animals.

The CF and I diets down-regulated more than twofold the expression of *Serpina10* (*log*2 FC −2.729 and −2.987, respectively), a gene codifying a serpin family member predominantly expressed in the liver and involved in the regulation of the coagulation factors’ activity, and *Apoa4* (*log*2 FC −1.955 and −1.273, respectively), a protein-coding gene that plays a role in lipoprotein metabolism (Table S1, supplementary material). On the contrary, these genes were not so affected by 10 % cocoa diet (*log*2 FC 0.475 and −0.645, respectively) (Table S1, supplementary material).

### Validation of microarray results by Real-Time PCR analysis

Eight genes representative of the affected pathways and with differential expression were selected for further analysis and validation of microarray results using quantitative RT-PCR. The studied genes included *Scgb1a1, Scnn1* *g* (highly up-regulated in the C10 group), *Tac4*, *Mcpt2, Fcer1a* and *Fabp1* (highly down-regulated in the C10 group). In addition, *Serpina10* (highly down-regulated in both the CF and I groups) and *Apoa4* (highly down-regulated in the CF group) were also studied.

**Fig. 4:**
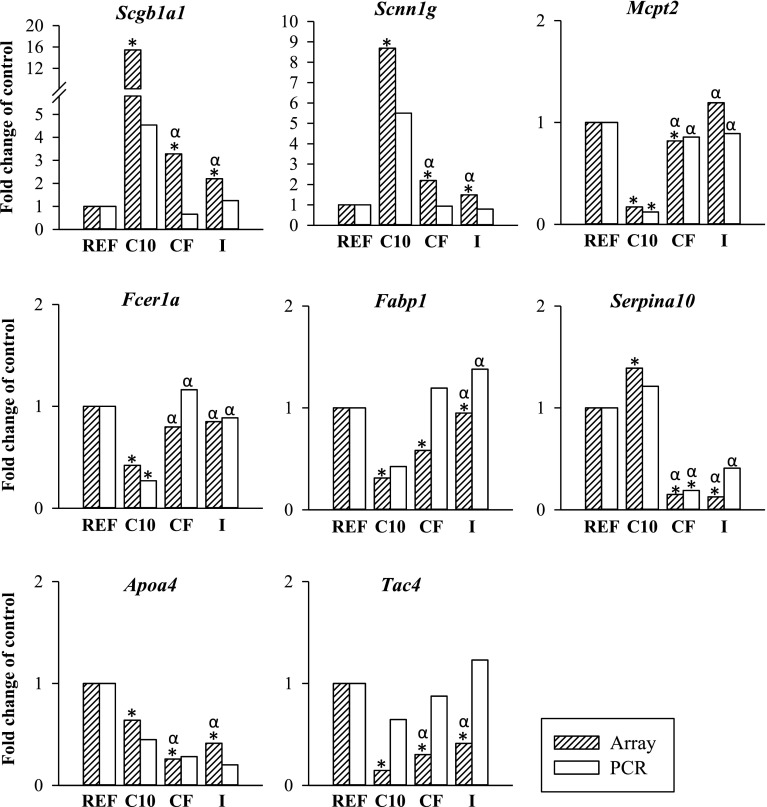
Expression of selected genes by real-time PCR. The values are presented as fold change in comparison with the REF group that is considered 1 (*n* = 10/group). *Filled bars* represent the expression for each specific gene as determined in the microarray experiments, and *empty bars* represent the expression for each specific gene as determined by PCR normalized by *Gusb*. Statistical significance: *P < 0.05 *vs* REF group; *α* *P* < 0.05 *vs* C10 group

Results are shown in Fig. 4, where the fold change expression determined both in the microarray and by PCR for each gene in each group is summarized. The quantitative PCR results are consistent with that of microarray. Although not all changes by PCR achieved statistical significance, a similar pattern of expression was determined using both techniques (Fig. 4). The statistical analysis of the real-time PCR results showed that only the expression of *Mcpt2* (*P* = 0.01) and *Fcer1a* (*P* = 0.01) was significantly down-regulated in the C10 group and that the cocoa fiber diet produced a significant down-regulation of *Serpina10* expression (*P* = 0.04).

## Discussion

We have previously reported that the intake of a 10 % cocoa diet in young rats induces changes in body weight and in the intestinal environment modulating the gut immune system and microbiota composition [13]. The involvement of cocoa fiber in such effects as well as its precise mechanism of action is unknown to date. In the present study, a microarray of the colon tissue RNA allowed the in-depth study of the modifications of gene expression induced in rats by either a 10 % cocoa diet or two other diets with the same proportion of soluble and insoluble fibers: one based on cocoa fiber and the other containing inulin.

Although the three experimental diets up- or down-regulated a large number of genes, the cocoa intake caused the most pronounced and numerous changes. The C10 diet was the one which resulted in a higher number of significantly overrepresented GO terms, with most of their genes being down-regulated. Moreover, there were much more interactions between the enriched GO terms after the C10 diet intake than those obtained after CF and I diets, with most of these interactions being among down-regulated genes.

A group of genes down-regulated by cocoa intake allowed us to study in-depth the mechanisms of this diet and its involvement in the immune function. In fact, the most down-regulated gene in the C10 group was that of tachykinin 4 (*Tac4*), which has been described as the promoter of B lineage cells [28]. As B lymphocytes are the immune cells responsible for antibody synthesis, these results could explain previous studies showing the cocoa-induced attenuation of immunoglobulin concentration in rats in both the systemic and intestinal compartments [15, 27, 29, 30]. This gene modification could also be responsible, at least partially, for the decrease in IgE synthesis in allergic animals induced by the 10 % cocoa diet [10] and even for the same effect reported by other polyphenols [31]. Focusing on allergy, it seems that cocoa intake could also be beneficial in aspects other than IgE synthesis because, as shown here, it down-regulates the expression of genes, such as *Fcer1a*, *Ms4a2* and *Tac4*, involved in several GO terms related to mast cell-mediated immunity, its activation and its degranulation, key events in allergic response. When considering the expression of these immune-related genes in the cocoa fiber and inulin groups, the effects were not so marked, and therefore, this may suggest that polyphenols or other cocoa compounds, but not cocoa fiber, are mainly responsible for the immunomodulatory properties of cocoa by interfering in the B cell ability to produce antibodies and also in the mast cell functionality.

A cocoa diet might also exert its immunomodulatory effects through the down-modulation of the expression of genes involved in cytokine production (*Fcer1a*, *Ms4a2* and *Adipoq*). In addition, cocoa diet in rats has demonstrated certain protective effects against chronic inflammation [32, 33] that could also be related to the down-regulation of the cytokine gene expression and also to the up-regulation of the expression of *Scgb1a1*, the gene that codifies a protein related to anti-inflammation [34]. In this context, both fiber diets—of cocoa and inulin—also increased the *Scgb1a1* gene expression, although in a lower intensity compared to that observed in the C10 group, suggesting that fiber is partially responsible for the anti-inflammatory effect of cocoa but that other cocoa compounds such as flavonoids must also be involved [35]. However, contrary to this suggestion, a significant down-regulation of *Scgb1a1* gene expression has been found in a lung tumor mice model treated for 20 weeks with green tea [36].

Another group of genes down-regulated by the 10 % cocoa diet was related to the lipid metabolism. In particular, the expression of *Adipoq,* an adipocyte-derived hormone involved in glucose and lipid metabolism, and *Fabp1*, an acid-binding protein found in liver, intestine and kidney involved in fatty acids uptake, transport and metabolism, was decreased by 1.826 and 1.682 FC logarithms. Moreover, the expression of *Angptl4,* a circulating lipoprotein lipase inhibitor, was up-regulated in the C10 group. Since these genes were less affected by the CF and I diets, we can suggest that the cocoa diet modifies lipid metabolism and this effect cannot be totally attributed to its fiber content and cocoa flavonoids are probably involved. The results agree with the reported hypolipidemic capacity of a cocoa diet [37–40] as well as in line with the results obtained, here and also in previous studies, on body weight [13, 27, 30, 41, 42]. In fact, the down-regulation of genes related to lipid metabolism has also been described by flavonoids, such as quercetin. The administration of quercetin in mice fed with a high-fat diet reduces the expression of hepatic genes related to lipid and fatty acid production, such as *Aldh1b1*, *Abcg5*, *Acaca*, *Fasn*, *Gpam* and *Apoa4* [38]. Although some other studies report body weight loss and a reduced fat deposition after polyphenol intake [38, 43, 44], previous studies carried out in our laboratory using three distinct cocoa materials with different proportions of polyphenols suggest that those effects on body weight cannot be exclusively attributed to cocoa polyphenols but also to other compounds present in cocoa [29]. As in the present study, we evidenced the lack of effect of the CF diet on body weight and on the same genes related to lipid metabolism as the entire cocoa diet, other bioactive compounds of cocoa, such as methylxanthines, could be proposed as being responsible for these effects on lipid metabolism gene expression proteins and body weight. In this context, methylxanthines are effective in stimulating resting energy expenditure per se [45] as well as regulating the lipid profile in obese–diabetic rats [46]. In addition, the synergistic interactions between methylxanthines and flavonoids in the thermogenesis have been suggested which could explain their effect on body weight [45].

Regarding cocoa fiber, in spite of the fact that it did not show the effects on the same lipid metabolism genes as cocoa, it is worth noting that the CF diet down-regulated the expression of another gene related to lipoprotein metabolism, the *Apoa4*. As also validated by RT-PCR, all three experimental diets down-regulated this gene, but the highest effect was observed after CF intake. These results could justify the hypolipidemic effect reported after consumption of either insoluble or soluble CF. In this context, the modulation of serum and liver lipid profile has been reported both in rats fed insoluble CF [18] and those fed soluble CF [19, 20]. *Apoa4* can also be associated with the response to superoxide and to oxygen radical pathways (GO:0000303 and GO:0000305, respectively); therefore, its down-regulation could elucidate the mechanism by which CF and cocoa flavanols are able to diminish the levels of certain lipid peroxidation biomarkers [39, 47]. From our results, we can also observe the cocoa effects on other genes associated with the antioxidant activity [39]. Thus, a significant increase in the expression of *Hba*-*a2* and *Hbb* genes, encoding alpha and beta betaglobin subunits, respectively, was found in both the C10 group and the CF group, although the influence was lower than a twofold change.

Another beneficial effect of cocoa intake concerns blood pressure. Cocoa flavanols have been described to possess antihypertensive properties [48], which have made them a subject matter of health claims by the European Food Safety Authority [49]. Possible feasible mechanisms whereby they decrease blood pressure could either be maintaining the endothelium-dependent vasodilatation [50], or inhibiting the angiotensin-converting enzyme (ACE) activity [51]. Partially in line with those mechanisms, in our study, a significant decrease in the expression of *Mcpt1* and *Mcpt4*—both genes involved in the regulation of systemic arterial blood pressure by renin–angiotensin (GO:0003081)—have been observed in 10 % cocoa-fed animals but not in the CF group. Nevertheless, the expression of the *Scnn1* *g* gene, involved in blood pressure homeostasis, was up-regulated in the C10 group and also in the CF animals, although with a <twofold change. In addition, both the CF diet and the inulin diet down-regulated by more than twofold the expression of *Serpina10*, a gene involved in the regulation of the coagulation factors’ activity, whose deficiency has been related to the risk of thrombosis [52]. Overall, our results show that cocoa intake modifies some colonic genes related to blood pressure regulation, and these effects cannot be attributed to its fiber content.

It remains to be elucidated which component from cocoa, but not cocoa fiber, is the main responsible for these changes in gene expression: both flavonoids and theobromine, present in high proportion in cocoa, are good candidates for such effects.

In summary, this study sheds light on the mechanisms involved in the reported effects of cocoa intake. As shown here, cocoa consumption down-regulates some genes related to the immune system (B cell and mast cell functionality), to lipid metabolism and also to blood pressure in the colon tissue. In addition, after comparing the effects of cocoa in its entirety and those produced by only cocoa fiber, it can be concluded that most of the beneficial effects attributed to cocoa are not only due to its fiber content but that polyphenols and other compounds could also be key factors.

## Electronic supplementary material

Below is the link to the electronic supplementary material.

Supplementary material 1 (PDF 418 kb)

Supplementary material 2 (DOC 107 kb)

Supplementary material 3 (DOC 133 kb)
