# Supplementary material for: Cocoa polyphenols and fiber modify colonic gene expression in rats
Source: Eur J Nutr. 2016 Jun 2;56(5):1871–85. doi: 10.1007/s00394-016-1230-0 (PMC5534200; doi:10.1007/s00394-016-1230-0)
Supplement: Supplementary file 1 — Supplementary material 1 (PDF 418 kb) [file 394_2016_1230_MOESM1_ESM.pdf]

		C10 group *vs* REF group		CF group *vs* REF group		I group *vs* REF group	
**Symbol**	**Genename**	**logFC**	***pvalue***	**logFC**	***pvalue***	**logFC**	***p value***
Scgb1a1	secretoglobin, family 1A, member 1 (uteroglobin)	3.948	0.000	1.716	0.000	1.139	0.001
Scnn1g	sodium channel, nonvoltage-gated 1, gamma	3.119	0.000	1.133	0.000	0.573	0.002
Fxyd4	FXYD domain-containing ion transport regulator 4	2.583	0.000	2.089	0.000	0.718	0.000
Slc15a1	solute carrier family 15 (oligopeptide transporter), member 1	2.332	0.000	1.388	0.000	0.475	0.010
Gbp1	guanylate binding protein 1, interferon-inducible	2.207	0.000	1.662	0.000	0.432	0.002
Cyp2f4	cytochrome P450, family 2, subfamily f, polypeptide 4	2.185	0.000	1.436	0.000	0.367	0.007
Dpysl4	dihydropyrimidinase-like 4	2.069	0.000	1.387	0.000	0.242	***0.951***
Fmo2	flavin containing monooxygenase 2	2.040	0.000	1.007	0.000	0.426	0.010
Pdyn	prodynorphin	1.873	0.000	0.194	***0.663***	0.175	***0.809***
Mt2A	metallothionein 2A	1.656	0.000	0.644	0.000	-0.147	0.000
Fmo2	flavin containing monooxygenase 2	1.637	0.000	0.826	0.000	0.272	0.011
RGD1561239	similar to guanylate binding protein 1, interferon-inducible, 67kDa	1.597	0.000	1.787	0.000	0.742	0.000
Sftpd	surfactant protein D	1.583	0.000	0.163	0.000	0.351	0.001
Mme	membrane metallo-endopeptidase	1.580	0.000	0.636	0.003	0.353	0.037
Hoxd13	homeo box D13	1.575	0.000	0.615	0.001	0.100	0.019
Chst5	carbohydrate (N-acetylglucosamine 6-O) sulfotransferase 5	1.533	0.000	0.591	0.000	0.082	0.003
Atp12a	ATPase, H+/K+ transporting, nongastric, alpha polypeptide	1.457	0.000	1.630	0.000	0.500	0.001
Thbs4	thrombospondin 4	1.450	0.000	0.908	0.000	0.302	0.023
Hoxd10	homeo box D10	1.403	0.000	0.668	0.001	0.164	***0.081***
RGD1561239	similar to guanylate binding protein 1, interferon-inducible, 67kDa	1.394	0.000	1.294	0.000	0.358	0.003
Trpv1	transient receptor potential cation channel, subfamily V, member 1	1.390	0.000	0.749	0.001	0.058	***0.147***
Hoxb13	homeo box B13	1.358	0.000	0.786	0.000	0.101	0.001
Spink3	serine peptidase inhibitor, Kazal type 3	1.338	0.000	1.162	0.000	0.463	0.003
LOC687303	similar to F35C5.3	1.327	0.000	0.734	0.000	0.755	0.000
Slc15a1	solute carrier family 15 (oligopeptide transporter), member 1	1.323	0.000	0.664	0.001	0.142	***0.827***
Abat	4-aminobutyrate aminotransferase	1.319	0.000	1.272	0.000	0.477	0.007
Angptl4	angiopoietin-like 4	1.316	0.000	0.914	0.000	0.670	0.000
Aox4	aldehyde oxidase 4	1.311	0.000	0.054	***0.083***	0.320	***0.051***
Mt2A	metallothionein 2A	1.308	0.000	0.522	0.001	-0.182	0.001
B3gnt7	UDP-GlcNAc:betaGal beta-1,3-N-acetylglucosaminyltransferase 7	1.265	0.000	0.403	0.001	0.019	0.012
Trpv1	transient receptor potential cation channel, subfamily V, member 1	1.260	0.000	0.595	0.002	-0.065	***0.169***
Pcsk9	proprotein convertase subtilisin/kexin type 9	1.253	0.001	0.048	0.035	0.063	0.023
RGD1560608	similar to novel protein	1.225	0.000	0.759	0.001	0.709	0.002
Guca2b	guanylate cyclase activator 2B	1.211	0.000	0.811	0.001	0.287	***0.051***
Padi3	peptidyl arginine deiminase, type III	1.190	0.000	0.832	0.001	0.462	0.012
Scnn1b	sodium channel, nonvoltage-gated 1, beta	1.181	0.001	0.499	0.010	0.057	***0.641***
LOC287167	globin, alpha	1.178	0.000	0.607	0.001	0.296	0.023
RGD1308274	similar to very large inducible GTPase-1	1.142	0.000	0.073	***0.531***	0.321	0.020
Smc1b	structural maintenance of chromosomes 1B	1.133	0.013	0.968	0.006	0.031	***0.548***
Pde7a	phosphodiesterase 7A	1.129	0.000	0.708	0.001	0.405	0.027
LOC100363350	rCG58102-like	1.126	0.000	0.621	0.001	0.446	0.004
Gsta2	glutathione S-transferase A2	1.111	0.001	0.537	0.004	0.157	***0.534***
Slc20a1	solute carrier family 20 (phosphate transporter), member 1	1.110	0.000	0.624	0.002	0.056	***0.403***
RGD1310110	similar to 3632451O06Rik protein	1.097	0.000	0.410	0.006	***0.231***	***0.053***
LOC689064	beta-globin	1.095	0.000	1.024	0.000	0.501	0.003
Rnase1	ribonuclease, RNase A family, 1 (pancreatic)	1.094	0.000	1.063	0.000	1.218	0.001
Slc15a1	solute carrier family 15 (oligopeptide transporter), member 1	1.092	0.000	0.665	0.001	***0.206***	***0.940***
Dnase1	deoxyribonuclease I	1.088	0.000	1.087	0.000	***0.124***	***0.483***
Pde7a	phosphodiesterase 7A	1.077	0.000	0.583	0.003	***0.217***	***0.718***
siat7D	alpha-2,6-sialyltransferase ST6GalNAc IV	1.058	0.000	0.363	0.020	***0.096***	***0.380***

Serpinc1	serpin peptidase inhibitor, clade C (antithrombin), member 1	1.053	0.000	0.873	0.000	0.469	0.013
LOC494499	LOC494499 protein	1.029	0.001	0.576	0.003	***0.276***	***0.051***
Fbxl13	F-box and leucine-rich repeat protein 13	1.028	0.000	1.009	0.000	***0.067***	***0.595***
Alox15	arachidonate 15-lipoxygenase	1.028	0.000	0.387	0.018	***0.191***	***0.951***
Creg1	cellular repressor of E1A-stimulated genes 1	1.021	0.000	0.406	0.019	***-0.008***	***0.515***
Rnase1l2	ribonuclease, RNase A family, 1-like 2 (pancreatic)	1.010	0.000	0.857	0.000	1.012	0.001
Alas2	aminolevulinate, delta-, synthase 2	1.007	0.000	0.997	0.000	0.452	0.005
LOC686412	similar to sal-like 4 isoform a	0.984	0.000	0.441	0.013	-0.128	0.036
Best2	bestrophin 2	0.984	0.000	1.018	0.000	0.320	0.006
Mt1a	metallothionein 1a	0.978	0.000	0.388	0.002	-0.372	0.002
S100a5	S100 calcium binding protein A5	0.973	0.001	0.431	***0.050***	0.328	0.045
Ces1c	carboxylesterase 1C	0.966	0.006	0.617	0.002	0.291	***0.929***
G6pc	glucose-6-phosphatase, catalytic subunit	0.963	0.010	0.129	***0.851***	-0.011	***0.537***
Tmem150c	transmembrane protein 150C	0.957	0.000	0.608	0.002	0.294	0.049
LOC498793	similar to inter-alpha-inhibitor H2 chain	0.952	0.000	0.822	0.001	0.308	0.033
Np4	defensin NP-4 precursor	0.943	0.045	-0.019	***0.502***	0.570	***0.110***
Sval1	seminal vesicle antigen-like 1	0.943	0.000	0.780	0.000	0.206	0.000
Dpp4	dipeptidylpeptidase 4	0.943	0.000	0.455	0.010	0.164	***0.496***
Aqp8	aquaporin 8	0.925	0.000	0.491	0.000	0.681	0.000
Tgm3	transglutaminase 3, E polypeptide	0.922	0.000	0.669	0.001	-0.016	0.017
Hba-a2	hemoglobin alpha, adult chain 2	0.919	0.000	0.992	0.000	0.483	0.004
Hbb	hemoglobin, beta	0.918	0.000	0.843	0.000	0.537	0.002
Fkbp5	FK506 binding protein 5	0.918	0.000	0.643	0.001	0.670	0.001
Dpp4	dipeptidylpeptidase 4	0.915	0.000	0.345	0.028	0.152	***0.084***
Tcf7	transcription factor 7, T-cell specific	0.904	0.004	0.405	0.016	1.006	0.002
Slc46a1	solute carrier family 46 (folate transporter), member 1	0.890	0.003	0.663	0.002	0.243	***0.690***
Pdzd3	PDZ domain containing 3	0.888	0.000	0.267	***0.938***	-0.124	***0.728***
Zyg11b	zyg-11 homolog B (C. elegans)	0.887	0.000	-0.628	0.018	0.076	***0.059***
Msh5	mutS homolog 5 (E. coli)	0.883	0.000	0.673	0.001	0.369	0.023
Hbe2	hemoglobin, epsilon 2	0.878	0.000	0.963	0.000	0.533	0.002
Dio1	deiodinase, iodothyronine, type I	0.872	0.001	0.218	0.039	-0.045	0.037
Papss2	3'-phosphoadenosine 5'-phosphosulfate synthase 2	0.867	0.000	0.765	0.001	0.459	0.011
Fam84a	family with sequence similarity 84, member A	0.859	0.000	0.535	0.004	0.348	0.023
Foxq1	forkhead box Q1	0.857	0.000	0.444	0.003	0.155	0.035
Ppat	phosphoribosyl pyrophosphate amidotransferase	0.852	0.000	0.614	0.002	0.349	0.018
Hbb-b1	hemoglobin, beta adult major chain	0.848	0.000	0.981	0.000	0.504	0.003
RGD1309350	similar to transthyretin (4L369)	0.844	0.000	0.324	***0.815***	-0.012	***0.395***
Mep1b	meprin 1 beta	0.843	0.000	0.342	0.029	0.060	***0.682***
Gadd45b	growth arrest and DNA-damage-inducible, beta	0.841	0.000	0.521	0.009	0.355	***0.783***
Papss2	3'-phosphoadenosine 5'-phosphosulfate synthase 2	0.839	0.000	0.681	0.001	0.408	0.012
Pdzk1ip1	PDZK1 interacting protein 1	0.836	0.001	0.110	0.042	-0.812	0.000
Ly6al	lymphocyte antigen 6 complex, locus A-like	0.833	0.000	0.240	***0.850***	-0.216	***0.930***
Hbb	hemoglobin, beta	0.831	0.000	0.994	0.000	0.613	0.001
Arg2	arginase type II	0.829	0.000	0.295	0.014	0.138	0.043
Sval1	seminal vesicle antigen-like 1	0.826	0.000	0.807	0.000	0.217	0.000
Fam55b	family with sequence similarity 55, member B	0.825	0.000	0.375	0.012	0.117	***0.073***
LOC691700	similar to heparan sulfate D-glucosaminyl 3-O-sulfotransferase 4	0.823	0.000	0.345	0.031	0.278	***0.055***
Hgfac	hepatocyte growth factor activator	0.822	0.000	0.217	***0.717***	0.106	***0.631***
Hbb-b1	hemoglobin, beta adult major chain	0.822	0.000	0.953	0.000	0.537	0.002
Tfcp2l1	transcription factor CP2-like 1	0.819	0.000	0.426	0.011	0.409	0.009
LOC499229	similar to very large inducible GTPase 1 isoform A	0.814	0.000	-0.126	***0.250***	0.371	0.004
Ceacam10	carcinoembryonic antigen-related cell adhesion molecule 10	0.812	0.000	0.268	0.010	0.337	0.017
Spink3	serine peptidase inhibitor, Kazal type 3	0.809	0.001	1.023	0.000	0.284	0.021
Krt75	keratin 75	0.801	0.001	0.424	0.015	0.083	***0.493***
Spata22	spermatogenesis associated 22	0.797	0.001	1.203	0.000	0.507	0.003
Cmah	cytidine monophosphate-N-acetylneuraminic acid hydroxylase	0.787	0.000	-0.230	0.004	-0.391	0.002
Ttc12	tetratricopeptide repeat domain 12	0.778	0.000	0.329	0.029	0.055	***0.503***
Hba-a2	hemoglobin alpha, adult chain 2	0.778	0.000	0.908	0.000	0.396	0.013
Hhex	hematopoietically expressed homeobox	0.775	0.002	0.461	0.019	0.285	***0.091***

Tef	thyrotrophic embryonic factor	0.774	0.000	0.370	0.017	0.079	***0.687***
Cd247	Cd247 molecule	0.773	0.002	0.856	0.000	1.010	0.000
Tmem71	transmembrane protein 71	0.763	0.023	0.222	0.046	0.066	***0.141***
Tm7sf2	transmembrane 7 superfamily member 2	0.759	0.001	0.287	0.022	0.020	***0.111***
Ckmt1	creatine kinase, mitochondrial 1	0.754	0.000	0.242	***0.731***	-0.056	***0.568***
Rnf125	ring finger protein 125	0.752	0.001	0.166	***0.850***	0.434	0.007
Trpv3	transient receptor potential cation channel, subfamily V, member 3	0.747	0.001	1.045	0.000	0.521	0.008
Unc119	UNC-119 homolog (C. elegans)	0.746	0.001	0.505	0.005	0.393	0.021
Cxcl13	chemokine (C-X-C motif) ligand 13	0.738	0.000	1.052	0.000	1.003	0.000
Arl4d	ADP-ribosylation factor-like 4D	0.725	0.001	0.068	***0.566***	-0.162	***0.816***
Prp2	proline rich protein 2	0.722	0.001	0.506	0.005	-0.120	***0.533***
Sftpc	surfactant protein C	0.722	0.008	0.399	***0.592***	-0.098	***0.841***
Epb41l3	erythrocyte membrane protein band 4.1-like 3	0.719	0.000	0.260	***0.892***	0.048	***0.701***
Zbtb16	zinc finger and BTB domain containing 16	0.719	0.002	0.376	0.026	0.510	0.003
Shisa8	shisa homolog 8 (Xenopus laevis)	0.716	0.000	0.297	***0.960***	0.218	***0.952***
Fam50b	family with sequence similarity 50, member B	0.713	0.024	0.567	0.045	-0.219	***0.938***
Tmem37	transmembrane protein 37	0.711	0.001	0.428	0.013	0.129	***0.820***
Slc26a2	solute carrier family 26 (sulfate transporter), member 2	0.706	0.002	0.086	***0.306***	0.226	***0.921***
Eno3	enolase 3, beta, muscle	0.706	0.001	1.202	0.000	0.342	0.006
Tnni1	troponin I type 1 (skeletal, slow)	0.704	0.005	0.715	0.004	0.226	***0.858***
Depdc7	DEP domain containing 7	0.702	0.000	0.244	***0.821***	0.272	0.040
Fam84a	family with sequence similarity 84, member A	0.701	0.001	0.589	0.001	0.408	0.013
Txnip	thioredoxin interacting protein	0.699	0.000	0.343	***0.725***	0.270	***0.915***
Banp	Btg3 associated nuclear protein	0.696	0.001	0.726	0.000	0.726	0.000
Ccdc153	coiled-coil domain containing 153	0.690	0.001	0.280	***0.895***	0.084	***0.414***
Il18	interleukin 18	0.690	0.001	0.154	***0.527***	0.267	0.033
LOC680692	similar to Golgi phosphoprotein 2 (Golgi membrane protein GP73)	0.689	0.001	0.138	***0.632***	-0.017	***0.480***
Fam111a	family with sequence similarity 111, member A	0.687	0.000	0.552	0.001	-1.022	0.000
RGD1562641	similar to hypothetical protein 4930474N05	0.687	0.002	0.390	0.034	-0.792	0.005
RGD1311575	hypothetical LOC289568	0.686	0.002	0.230	***0.692***	0.006	***0.547***
Unc13c	unc-13 homolog C (C. elegans)	0.686	0.041	0.721	0.018	0.096	***0.879***
Clec2e	C-type lectin domain family 2, member E	0.685	0.001	0.020	***0.316***	0.022	***0.462***
LOC305806	similar to glutaredoxin 1 (thioltransferase); glutaredoxin	0.685	0.028	0.117	***0.660***	0.476	0.007
Tpt1	tumor protein, translationally-controlled 1	0.685	0.001	0.330	0.035	0.004	***0.162***
Slc4a1	solute carrier family 4 (anion exchanger), member 1	0.685	0.000	0.261	***0.864***	0.027	***0.631***
Uncx	UNC homeobox	0.683	0.003	1.093	0.000	0.283	0.023
LOC300024	similar to Ly6-B antigen gene	0.680	0.001	0.173	***0.688***	-0.291	***0.962***
Cideb	cell death-inducing DFFA-like effector b	0.679	0.001	-0.169	***0.782***	-0.306	0.020
Foxa2	forkhead box A2	0.679	0.001	0.166	***0.614***	0.192	***0.813***
LOC680787	rCG56785-like	0.678	0.037	0.609	0.023	-0.400	0.013
Actc1	actin, alpha, cardiac muscle 1	0.675	0.002	0.152	***0.821***	-0.029	***0.517***
Rcbtb1	regulator of chromosome condensation (RCC1) and BTB (POZ) domain containing protein 1	0.674	0.001	0.406	0.029	0.282	***0.051***
Cyp3a18	cytochrome P450, family 3, subfamily a, polypeptide 18	0.674	0.002	0.224	***0.910***	-0.053	***0.561***
Cib2	calcium and integrin binding family member 2	0.672	0.002	0.174	***0.827***	-0.248	***0.923***
S100a5	S100 calcium binding protein A5	0.670	0.001	0.268	0.018	0.221	0.027
Tnn	tenascin N	0.669	0.002	0.716	0.005	1.033	0.002
Tcf7l2	transcription factor 7-like 2 (T-cell specific, HMG-box)	0.668	0.002	0.139	***0.376***	0.361	0.022
LOC100361909	eosinophil-associated ribonuclease 3-like	0.667	0.009	0.418	0.008	0.060	***0.573***
Epb41l3	erythrocyte membrane protein band 4.1-like 3	0.667	0.001	0.216	***0.852***	0.039	***0.628***
Cd177	CD177 molecule	0.666	0.001	-0.042	***0.417***	0.039	***0.438***
Pbld	phenazine biosynthesis-like protein domain containing	0.665	0.001	0.227	***0.887***	-0.147	***0.706***
Hist1h1b	histone cluster 1, H1b	0.665	0.001	0.114	***0.765***	0.174	***0.880***
Fam25a	family with sequence similarity 25, member A	0.665	0.018	0.248	***0.737***	0.270	0.041
Inhbb	inhibin beta-B	0.663	0.002	0.069	***0.153***	-0.058	***0.271***
LOC500300	similar to hypothetical protein MGC6835	0.663	0.002	1.284	0.000	0.857	0.000
Eif4ebp3	eukaryotic translation initiation factor 4E binding protein 3	0.662	0.001	0.250	***0.953***	0.094	***0.779***

Cyp4f17	cytochrome P450, family 4, subfamily f, polypeptide 17	0.659	0.000	0.309	0.005	0.268	0.003
Ccrl2	chemokine (C-C motif) receptor-like 2	0.658	0.002	0.383	0.019	-0.030	***0.422***
Gnat2	guanine nucleotide binding protein (G protein), alpha transducing activity polypeptide 2	0.657	0.005	0.493	0.020	0.114	***0.796***
Rab20	RAB20, member RAS oncogene family	0.655	0.001	0.389	0.015	0.235	***0.933***
Vwa5b2	von Willebrand factor A domain containing 5B2	0.652	0.001	0.127	***0.658***	0.116	***0.575***
Slc25a45	solute carrier family 25, member 45	0.650	0.003	0.665	0.003	0.281	***0.051***
P2ry6	pyrimidinergic receptor P2Y, G-protein coupled, 6	0.650	0.002	0.525	0.004	0.287	***0.053***
RT1-CE12	RT1 class I, locus CE12	0.648	0.002	0.174	***0.537***	-0.439	0.004
Pigzl1	phosphatidylinositol glycan, class Z-like 1	0.644	0.000	0.023	0.009	-0.459	0.001
Pm20d1	peptidase M20 domain containing 1	0.644	0.004	0.252	***0.841***	0.017	***0.493***
Hist1h1b	histone cluster 1, H1b	0.641	0.002	0.202	***0.889***	0.220	***0.922***
Anxa3	annexin A3	0.640	0.001	0.103	***0.427***	-0.087	***0.287***
Ndrg2	N-myc downstream regulated gene 2	0.639	0.001	0.283	***0.802***	0.128	***0.692***
LOC367436	similar to Y-linked testis-specific protein	0.634	0.008	0.177	***0.468***	0.501	0.002
Hist3h2ba	histone cluster 3, H2ba	0.634	0.003	0.173	***0.790***	0.118	***0.825***
Enpp2	ectonucleotide pyrophosphatase/phosphodiesterase 2	0.633	0.000	0.350	0.003	0.507	0.006
Bsnd	Bartter syndrome, infantile, with sensorineural deafness (Barttin)	0.633	0.001	0.497	0.006	0.040	***0.691***
Gpr150	G protein-coupled receptor 150	0.630	0.037	0.462	0.011	0.391	0.038
Pou2af1	POU class 2 associating factor 1	0.629	0.001	0.607	0.001	1.184	0.000
Cbln1	cerebellin 1 precursor	0.626	0.008	0.248	***0.063***	0.190	***0.092***
Lrrc8e	leucine rich repeat containing 8 family, member E	0.625	0.001	0.223	***0.743***	-0.101	***0.727***
Slc6a14	solute carrier family 6 (amino acid transporter), member 14	0.622	0.001	0.607	0.001	0.453	0.007
Slc25a34	solute carrier family 25, member 34	0.621	0.002	-0.074	***0.707***	-0.160	***0.716***
Taok3	TAO kinase 3	0.621	0.003	0.181	***0.334***	0.407	0.013
Sgk1	serum/glucocorticoid regulated kinase 1	0.619	0.001	0.063	***0.160***	-0.283	0.026
Tmem132c	transmembrane protein 132C	0.616	0.002	0.589	0.002	-0.048	***0.524***
Cyp4a8	cytochrome P450, family 4, subfamily a, polypeptide 8	0.616	0.009	0.506	0.014	0.743	0.000
Rt1.aa	MHC class I RT1.Aa alpha-chain	0.615	0.003	0.472	0.003	0.605	0.001
Wnt16	wingless-type MMTV integration site family, member 16	0.615	0.003	0.453	0.012	0.496	0.005
RT1-CE14	RT1 class I, locus CE14	0.614	0.008	0.426	0.009	0.520	0.003
Sh3d21	SH3 domain containing 21	0.612	0.006	0.346	***0.972***	0.389	0.020
Pck1	phosphoenolpyruvate carboxykinase 1 (soluble)	0.609	0.004	-0.424	0.010	-0.327	***0.972***
Ccr7	chemokine (C-C motif) receptor 7	0.608	0.008	0.523	0.020	0.727	0.004
Cabyr	calcium binding tyrosine-(Y)-phosphorylation regulated	0.607	0.004	0.433	0.008	0.168	***0.810***
Gpr160	G protein-coupled receptor 160	0.607	0.004	0.078	***0.821***	0.003	***0.457***
Ppp1r9a	protein phosphatase 1, regulatory subunit 9A	0.607	0.003	0.503	0.008	0.358	0.029
Bco2	beta-carotene oxygenase 2	0.607	0.005	0.196	***0.757***	0.037	***0.422***
Slc22a5	solute carrier family 22 (organic cation/carnitine transporter), member 5	0.605	0.005	0.004	***0.238***	-0.119	***0.495***
RT1-CE16	RT1 class I, locus CE16	0.605	0.001	0.378	0.005	0.436	0.006
Slc34a2	solute carrier family 34 (sodium phosphate), member 2	0.604	0.003	0.613	0.002	0.269	***0.062***
Grin2d	glutamate receptor, ionotropic, N-methyl D-aspartate 2D	0.601	0.002	0.301	***0.942***	0.185	***0.766***
Grap	GRB2-related adaptor protein	0.600	0.014	0.493	0.021	0.695	0.006
Cyp2d4	cytochrome P450, family 2, subfamily d, polypeptide 4	0.599	0.003	0.391	0.025	0.104	***0.805***
Lef1	lymphoid enhancer binding factor 1	0.599	0.012	0.308	***0.065***	0.575	0.007
Klf11	Kruppel-like factor 11	0.598	0.002	0.342	***0.984***	0.321	0.039
Ces2g	carboxylesterase 2G	0.598	0.002	0.270	***0.660***	0.007	***0.449***
Ttc36	tetratricopeptide repeat domain 36	0.596	0.005	1.113	0.000	0.281	0.016
LOC679149	similar to carboxylesterase 2 (intestine, liver)	0.596	0.001	0.129	***0.070***	-0.240	0.018
Tfcp2l1	transcription factor CP2-like 1	0.595	0.002	0.213	***0.738***	0.291	***0.965***
Tmem140	transmembrane protein 140	0.592	0.002	0.036	***0.340***	-0.036	***0.552***
Ms4a10	membrane-spanning 4-domains, subfamily A, member 10	0.591	0.007	0.293	***0.504***	-0.311	***0.980***
Ptprr	protein tyrosine phosphatase, receptor type, R	0.591	0.002	0.442	0.008	0.003	***0.325***
RGD1304644	similar to RIKEN cDNA 2310046K01	0.589	0.002	0.293	***0.722***	-0.058	***0.450***
Slc41a3	solute carrier family 41, member 3	0.589	0.002	0.200	***0.770***	-0.008	***0.411***

St6galnac2	ST6 (alpha-N-acetyl-neuraminyl-2,3-beta-galactosyl-1,3)-N-acetylgalactosaminide alpha-2,6-sialyltransferase 2	0.589	0.003	0.088	***0.799***	-0.201	***0.905***
Slc45a3	solute carrier family 45, member 3	0.586	0.008	0.180	***0.736***	0.062	***0.397***
Fam159b	family with sequence similarity 159, member B	0.584	0.005	0.861	0.000	0.345	0.010
Qrich2	glutamine rich 2	0.581	0.002	0.218	0.026	0.036	***0.083***
Peg12	paternally expressed 12	0.581	0.003	0.199	***0.854***	0.039	***0.614***
LOC681325	hypothetical protein LOC681325	0.580	0.015	0.494	0.010	0.620	0.013
B3gnt8	UDP-GlcNAc:betaGal beta-1,3-N-acetylglucosaminyltransferase 8	0.580	0.001	0.120	***0.394***	-0.039	***0.194***
RGD1310587	similar to hypothetical protein FLJ14146	0.576	0.002	-0.296	***0.853***	-0.385	0.021
Ccnd2	cyclin D2	0.575	0.002	0.605	0.001	0.483	0.005
Acat3	acetyl-Coenzyme A acetyltransferase 3	0.575	0.002	0.015	***0.299***	-0.128	0.044
Hoxd9	homeo box D9	0.574	0.011	0.455	0.007	0.157	***0.809***
Clcnkb	chloride channel Kb	0.573	0.002	0.064	***0.332***	-0.572	0.002
Fads3	fatty acid desaturase 3	0.573	0.004	0.439	0.009	0.177	***0.525***
Ctse	cathepsin E	0.573	0.002	0.202	***0.811***	-0.037	***0.386***
LOC686432	hypothetical protein LOC686432	0.570	0.003	-0.137	***0.771***	-0.279	***0.959***
Cyp2j3	cytochrome P450, family 2, subfamily j, polypeptide 3	0.568	0.004	0.030	***0.415***	0.128	***0.853***
Hist3h2bb	histone cluster 3, H2bb	0.568	0.005	0.198	***0.859***	0.027	***0.642***
Pdk4	pyruvate dehydrogenase kinase, isozyme 4	0.567	0.003	0.352	0.031	0.284	***0.054***
RGD1561465	similar to RIKEN cDNA 2900010J23	0.567	0.005	0.532	0.008	-0.099	***0.397***
LOC679861	similar to MIC2 like 1	0.565	0.002	0.396	0.010	0.239	0.030
Gfpt2	glutamine-fructose-6-phosphate transaminase 2	0.565	0.004	0.199	***0.790***	0.109	***0.744***
Kank3	KN motif and ankyrin repeat domains 3	0.564	0.005	0.396	0.018	0.115	***0.684***
Vom2r73	vomeronasal 2 receptor, 73	0.562	0.004	0.423	0.032	0.043	***0.379***
RGD1308305	similar to RIKEN cDNA 5430400H23	0.562	0.008	0.405	0.016	-0.208	***0.578***
Tle4	transducin-like enhancer of split 4 (E(sp1) homolog, Drosophila)	0.562	0.005	0.546	0.002	0.599	0.001
Upp1	uridine phosphorylase 1	0.562	0.002	-0.041	***0.621***	-0.008	***0.442***
RGD1306227	similar to 4833420G17Rik protein	0.561	0.003	0.070	***0.621***	-0.052	***0.625***
Slc25a30	solute carrier family 25, member 30	0.561	0.004	0.187	***0.606***	0.124	***0.490***
Fcrl1	Fc receptor-like 1	0.561	0.001	0.818	0.000	0.989	0.001
Cr2	complement receptor 2	0.561	0.001	0.678	0.001	0.951	0.000
Ly6d	lymphocyte antigen 6 complex, locus D	0.560	0.015	0.443	0.033	0.420	0.029
Fam111a	family with sequence similarity 111, member A	0.559	0.002	0.475	0.002	-0.930	0.000
RGD1563652	similar to novel MAM domain containing protein	0.558	0.006	-0.246	0.019	0.136	***0.171***
Acadsb	acyl-CoA dehydrogenase, short/branched chain	0.558	0.002	0.262	0.030	0.724	0.000
Cbx2	chromobox homolog 2	0.557	0.010	0.198	***0.881***	0.065	***0.289***
Stk39	serine threonine kinase 39	0.557	0.003	0.245	***0.847***	-0.037	***0.590***
Pcmtd2	protein-L-isoaspartate (D-aspartate) O-methyltransferase domain containing 2	0.556	0.003	0.138	***0.927***	0.228	***0.956***
Vpreb3	pre-B lymphocyte 3	0.554	0.004	0.506	0.005	0.858	0.001
A1i3	alpha-1-inhibitor III	0.554	0.017	0.128	***0.857***	-0.010	***0.517***
Erbb3	v-erb-b2 erythroblastic leukemia viral oncogene homolog 3 (avian)	0.553	0.003	-0.009	***0.618***	-0.065	***0.773***
Osgin1	oxidative stress induced growth inhibitor 1	0.552	0.006	0.236	***0.899***	0.010	***0.469***
Dlk1	delta-like 1 homolog (Drosophila)	0.552	0.004	1.109	0.000	0.311	0.008
Akr1cl1	aldo-keto reductase family 1, member C-like 1	0.551	0.003	0.868	0.000	0.425	0.004
Kcns2	potassium voltage-gated channel, delayed-rectifier, subfamily S, member 2	0.551	0.007	0.743	0.002	0.254	0.047
Atp2c2	ATPase, Ca++ transporting, type 2C, member 2	0.551	0.004	0.103	***0.506***	0.175	***0.801***
LOC690082	similar to melanoma ubiquitous mutated protein	0.551	0.003	0.291	0.033	0.210	***0.811***
Ltb	lymphotoxin beta (TNF superfamily, member 3)	0.549	0.003	0.513	0.003	0.696	0.001
Osbpl6	oxysterol binding protein-like 6	0.549	0.004	0.276	***0.750***	0.125	***0.820***
Fsip2	fibrous sheath-interacting protein 2	0.547	0.006	0.319	0.030	-0.064	***0.579***
Vom1r98	vomeronasal 1 receptor 98	0.546	0.006	0.744	0.001	0.352	0.025
Defa24	defensin, alpha, 24	0.545	0.008	-0.523	0.010	-0.528	0.004
RGD1304563	similar to RIKEN cDNA 4831426I19	0.545	0.008	0.352	0.024	0.137	***0.559***
Aqp5	aquaporin 5	0.545	0.018	0.370	***0.772***	-0.108	***0.751***
Tle3	transducin-like enhancer of split 3 (E(sp1) homolog, Drosophila)	0.545	0.005	0.312	***0.973***	0.241	***0.915***
Pgap1	post-GPI attachment to proteins 1	0.544	0.004	0.252	***0.454***	0.272	***0.813***
Fam126b	family with sequence similarity 126, member B	0.543	0.006	0.184	***0.638***	0.027	***0.448***
Prrc2c	proline-rich coiled-coil 2C	0.542	0.004	0.316	***0.899***	0.295	***0.052***

Acvr2b	activin A receptor, type IIB	0.541	0.004	0.275	***0.903***	0.108	***0.713***
Acvr2b	activin A receptor, type IIB	0.540	0.004	0.115	***0.721***	0.009	***0.602***
LOC100365551	hypothetical protein LOC100365551	0.539	0.009	0.075	***0.690***	0.057	***0.515***
Gimap1	GTPase, IMAP family member 1	0.536	0.001	0.276	0.014	0.489	0.002
Ets2	v-ets erythroblastosis virus E26 oncogene homolog 2 (avian)	0.536	0.003	0.167	***0.416***	0.186	***0.596***
Slc9a2	solute carrier family 9 (sodium/hydrogen exchanger), member 2	0.536	0.004	0.163	***0.532***	0.018	***0.339***
Fads2	fatty acid desaturase 2	0.536	0.006	0.386	0.020	0.140	***0.856***
Aspg	asparaginase homolog (S. cerevisiae)	0.536	0.021	-0.099	***0.441***	-0.234	0.016
Piga	phosphatidylinositol glycan anchor biosynthesis, class A	0.535	0.006	0.221	***0.703***	0.072	***0.739***
LOC501937	similar to PRAME family member 8	0.535	0.008	0.421	0.015	0.447	0.008
Prkacb	protein kinase, cAMP dependent, catalytic, beta	0.535	0.001	0.067	0.020	0.114	0.018
LOC690120	hypothetical protein LOC690120	0.534	0.002	0.058	***0.446***	-0.031	***0.170***
Tm4sf20	transmembrane 4 L six family member 20	0.534	0.003	0.597	0.001	0.447	0.004
Pkp1	plakophilin 1	0.534	0.005	0.264	0.037	0.225	***0.068***
Cmya5	cardiomyopathy associated 5	0.533	0.006	0.556	0.005	0.216	***0.681***
Irs2	insulin receptor substrate 2	0.532	0.004	0.255	***0.792***	0.268	***0.052***
Fmo4	flavin containing monooxygenase 4	0.531	0.006	0.035	***0.571***	-0.086	***0.600***
RGD1309873	similar to hypothetical protein BC010003	0.530	0.003	0.335	0.016	0.254	0.028
RGD1560691	similar to calcium/calmodulin-dependent protein kinase 1D	0.530	0.002	0.245	***0.668***	0.129	***0.658***
RGD1566265	similar to RIKEN cDNA 2610002M06	0.529	0.031	0.411	0.010	0.217	***0.790***
Slc22a3	solute carrier family 22 (extraneuronal monoamine transporter), member 3	0.528	0.012	0.152	***0.415***	0.281	***0.050***
RGD1563692	similar to hypothetical protein FLJ22671	0.527	0.005	0.211	***0.685***	0.244	***0.051***
Hnrnpa1	heterogeneous nuclear ribonucleoprotein A1	0.526	0.007	0.358	***0.954***	0.391	0.022
Bco2	beta-carotene oxygenase 2	0.526	0.013	0.303	***0.922***	0.122	***0.693***
Trpm6	transient receptor potential cation channel, subfamily M, member 6	0.526	0.005	0.367	0.029	0.138	***0.503***
Atp2b1	ATPase, Ca++ transporting, plasma membrane 1	0.525	0.005	0.321	0.024	0.052	***0.670***
Slc12a6	solute carrier family 12, member 6	0.524	0.005	0.039	***0.452***	0.050	***0.432***
Anxa13	annexin A13	0.524	0.001	0.352	0.007	0.507	0.003
Bche	butyrylcholinesterase	0.523	0.008	0.197	***0.739***	0.231	***0.904***
Pde6h	phosphodiesterase 6H, cGMP-specific, cone, gamma	0.523	0.003	0.611	0.004	0.743	0.003
Lrg1	leucine-rich alpha-2-glycoprotein 1	0.522	0.002	0.262	***0.684***	0.083	***0.382***
Ocln	occludin	0.521	0.006	-0.028	***0.621***	-0.019	***0.594***
Slc4a7	solute carrier family 4, sodium bicarbonate cotransporter, member 7	0.521	0.003	0.401	0.012	0.641	0.002
Fabp6	fatty acid binding protein 6, ileal	0.521	0.004	-0.705	0.006	-0.403	0.003
Trim25	tripartite motif-containing 25	0.520	0.004	0.195	***0.847***	0.219	***0.933***
Slc7a13	solute carrier family 7 (anionic amino acid transporter), member 13	0.519	0.004	0.422	0.010	0.250	0.048
Acrbp	acrosin binding protein	0.519	0.009	0.287	***0.893***	0.188	***0.772***
Fam189a2	family with sequence similarity 189, member A2	0.518	0.004	-0.149	***0.788***	-0.051	***0.526***
Arrdc3	arrestin domain containing 3	0.517	0.004	0.134	***0.484***	0.169	***0.819***
Lta	lymphotoxin alpha (TNF superfamily, member 1)	0.517	0.015	0.154	***0.073***	0.310	0.030
Wdr38	WD repeat domain 38	0.517	0.013	0.620	0.005	0.115	***0.539***
Vcam1	vascular cell adhesion molecule 1	0.517	0.007	0.084	***0.132***	0.141	***0.192***
Grm4	glutamate receptor, metabotropic 4	0.516	0.007	0.343	***0.916***	0.273	***0.957***
Bmp7	bone morphogenetic protein 7	0.513	0.008	0.061	***0.783***	-0.180	***0.956***
Chka	choline kinase alpha	0.513	0.007	0.270	***0.821***	0.342	0.037
Ccl19	chemokine (C-C motif) ligand 19	0.512	0.000	0.756	0.000	0.998	0.000
Sprr1b	small proline-rich protein 1B	0.512	0.004	0.363	0.014	-0.119	***0.445***
Ubd	ubiquitin D	0.511	0.000	0.577	0.000	0.500	0.000
LOC688155	similar to Beta-1,4 N-acetylgalactosaminyltransferase 2	0.511	0.023	0.661	0.002	-0.042	0.009
Spib	Spi-B transcription factor (Spi-1/PU.1 related)	0.510	0.001	0.687	0.000	0.577	0.002
Clcn3	chloride channel 3	0.510	0.008	0.072	***0.517***	-0.032	***0.690***
Ovol1	ovo-like 1(Drosophila)	0.510	0.008	0.193	***0.933***	-0.027	***0.601***
Wnt11	wingless-type MMTV integration site family, member 11	0.509	0.006	-0.091	***0.558***	-0.043	***0.482***
RGD1562534	similar to Insulin-like growth factor binding protein 4 precursor (IGFBP-4)	0.507	0.032	0.492	0.045	-0.267	***0.936***

Cyp27a1	cytochrome P450, family 27, subfamily a, polypeptide 1	0.507	0.007	0.068	***0.789***	-0.081	***0.678***
A2m	alpha-2-macroglobulin	0.506	0.004	0.458	0.006	0.376	0.033
Prrc2c	proline-rich coiled-coil 2C	0.505	0.004	0.172	***0.588***	0.139	***0.539***
Cyp26b1	cytochrome P450, family 26, subfamily b, polypeptide 1	0.504	0.010	0.582	0.005	0.488	0.004
Gk	glycerol kinase	0.503	0.011	0.129	***0.800***	-0.035	***0.514***
Ccl3	chemokine (C-C motif) ligand 3	0.502	0.008	1.193	0.000	0.805	0.000
Slc45a3	solute carrier family 45, member 3	0.502	0.007	0.044	***0.575***	-0.008	***0.561***
LOC100365604	neuroblastoma-amplified protein-like	0.502	0.007	-0.018	***0.604***	-0.123	***0.852***
Slc11a2	solute carrier family 11 (proton-coupled divalent metal ion transporters), member 2	0.502	0.008	0.326	***0.990***	-0.087	***0.820***
Olr1201	olfactory receptor 1201	0.502	0.049	0.341	0.035	0.146	***0.814***
LOC678918	similar to NADH dehydrogenase (ubiquinone) 1 beta subcomplex 3	0.501	0.015	0.440	0.017	-0.095	***0.685***
LOC686260	hypothetical protein LOC686260	0.501	0.017	0.164	***0.896***	0.192	***0.958***
RGD1559459	similar to Expressed sequence AI788959	0.501	0.006	0.120	***0.365***	0.025	***0.334***
Uts2d	urotensin 2 domain containing	0.501	0.011	0.032	***0.724***	0.117	***0.674***
Clybl	citrate lyase beta like	0.500	0.002	0.545	0.001	0.457	0.002
Slc5a3	solute carrier family 5 (sodium/myo-inositol cotransporter), member 3	0.500	0.004	0.321	0.020	0.245	0.033
B3galt2	UDP-Gal:betaGlcNAc beta 1,3-galactosyltransferase, polypeptide 2	0.500	0.005	-0.201	***0.789***	-0.027	***0.399***
Rhoh	ras homolog gene family, member H	0.500	0.006	0.747	0.002	0.913	0.001
Btnl2	butyrophilin-like 2	0.499	0.008	0.135	***0.652***	0.036	***0.671***
RGD1565690	similar to mKIAA2027 protein	0.499	0.009	0.111	***0.747***	0.012	***0.395***
Slc5a11	solute carrier family 5 (sodium/glucose cotransporter), member 11	0.499	0.005	0.411	0.010	0.424	0.008
Spa17	sperm autoantigenic protein 17	0.497	0.010	-0.054	***0.675***	-0.005	***0.596***
Irgq	immunity-related GTPase family, Q	0.497	0.005	0.104	***0.626***	-0.271	0.013
Psd	pleckstrin and Sec7 domain containing	0.495	0.012	0.052	***0.724***	0.120	***0.810***
Tgif1	TGFB-induced factor homeobox 1	0.495	0.007	0.218	***0.960***	0.285	***0.987***
Cd1d1	CD1d1 molecule	0.495	0.005	0.108	***0.481***	0.030	***0.433***
Lpar2	lysophosphatidic acid receptor 2	0.495	0.009	0.032	***0.568***	0.174	***0.902***
B3galt2	UDP-Gal:betaGlcNAc beta 1,3-galactosyltransferase, polypeptide 2	0.495	0.007	-0.212	***0.785***	-0.076	***0.366***
Slc2a13	solute carrier family 2 (facilitated glucose transporter), member 13	0.495	0.004	-0.137	***0.739***	0.014	***0.569***
Per3	period homolog 3 (Drosophila)	0.495	0.007	0.281	***0.957***	0.057	***0.693***
Med15	mediator complex subunit 15	0.494	0.010	0.224	***0.875***	0.251	***0.940***
RGD1311575	hypothetical LOC289568	0.494	0.009	0.191	***0.528***	0.217	***0.887***
Il21r	interleukin 21 receptor	0.494	0.003	0.536	0.005	0.888	0.000
Rnf186	ring finger protein 186	0.493	0.008	0.201	***0.845***	0.005	***0.516***
Olr1462	olfactory receptor 1462	0.493	0.008	0.910	0.000	0.462	0.003
Ces2e	carboxylesterase 2E	0.491	0.006	0.212	***0.740***	-0.148	***0.635***
LOC691670	similar to natural killer cell protease 7	0.488	0.037	0.821	0.000	0.566	0.002
Tinag	tubulointerstitial nephritis antigen	0.488	0.010	0.122	***0.805***	-0.115	***0.714***
Ccdc48	coiled-coil domain containing 48	0.487	0.008	0.128	***0.513***	0.109	***0.741***
C2	complement component 2	0.487	0.001	0.247	0.005	-0.027	***0.058***
Lpcat1	lysophosphatidylcholine acyltransferase 1	0.486	0.005	0.037	***0.517***	-0.204	***0.827***
Traf3ip3	TRAF3 interacting protein 3	0.486	0.015	0.417	0.024	0.583	0.009
Cyp2j3	cytochrome P450, family 2, subfamily j, polypeptide 3	0.486	0.008	-0.025	***0.506***	0.060	***0.661***
B3gnt5	UDP-GlcNAc:betaGal beta-1,3-N-acetylglucosaminyltransferase 5	0.485	0.001	0.230	0.011	0.172	0.021
Gimap5	GTPase, IMAP family member 5	0.485	0.022	0.498	0.016	0.631	0.013
Reln	reelin	0.484	0.010	0.293	***0.990***	0.121	***0.902***
Mocos	molybdenum cofactor sulfurase	0.484	0.008	0.098	***0.708***	0.007	***0.643***
Ulk4	unc-51-like kinase 4 (C. elegans)	0.483	0.009	0.127	***0.821***	0.036	***0.625***
RGD1562865	similar to BTB and CNC homology 1, basic leucine zipper transcription factor 2	0.483	0.006	0.541	0.006	0.571	0.015
Camsap2	calmodulin regulated spectrin-associated protein family, member 2	0.482	0.007	0.189	***0.786***	0.232	***0.903***
Clu	clusterin	0.482	0.001	0.325	0.004	0.449	0.005
Sash3	SAM and SH3 domain containing 3	0.482	0.029	0.551	0.026	0.738	0.005
Cyp3a9	cytochrome P450, family 3, subfamily a, polypeptide 9	0.481	0.010	0.167	***0.636***	0.149	***0.820***

Lgr4	leucine-rich repeat-containing G protein-coupled receptor 4	0.480	0.009	0.057	***0.355***	0.077	***0.766***
Fgd4	FYVE, RhoGEF and PH domain containing 4	0.479	0.009	0.056	***0.416***	0.014	***0.693***
RGD1565170	similar to 60S ribosomal protein L23a	0.479	0.008	0.168	***0.777***	0.125	***0.653***
Olr1694	olfactory receptor 1694	0.479	0.012	0.892	0.001	0.142	***0.829***
Tnfrsf13c	tumor necrosis factor receptor superfamily, member 13c	0.478	0.005	0.805	0.005	0.770	0.001
Myog	myogenin	0.475	0.009	0.259	***0.758***	0.288	0.038
Slc16a10	solute carrier family 16 (monocarboxylic acid transporters), member 10	0.475	0.009	0.139	***0.908***	-0.014	***0.604***
Hk1	hexokinase 1	0.475	0.010	0.316	***0.925***	0.187	***0.766***
Nr1i3	nuclear receptor subfamily 1, group I, member 3	0.475	0.007	0.386	0.015	0.031	***0.455***
RT1-Bb	RT1 class II, locus Bb	0.475	0.000	0.512	0.000	0.737	0.000
Serpina10	serine (or cysteine) peptidase inhibitor, clade A (alpha-1 antiproteinase, antitrypsin), member 10	0.475	0.000	-2.729	0.000	-2.987	0.000
C2	complement component 2	0.475	0.003	0.334	0.007	0.134	0.033
Grhl3	grainyhead-like 3 (Drosophila)	0.474	0.004	0.110	***0.518***	0.453	0.006
Sorcs1	sortilin-related VPS10 domain containing receptor 1	0.474	0.016	0.325	0.026	0.344	0.044
Hist2h2be	histone cluster 2, H2be	0.473	0.009	0.136	***0.554***	-0.147	***0.629***
Gga2	golgi associated, gamma adaptin ear containing, ARF binding protein 2	0.472	0.041	0.506	***0.583***	0.618	0.013
Sh2d1a	SH2 domain containing 1A	0.472	0.038	0.360	***0.104***	0.760	0.008
Kiaa0895l	hypothetical protein LOC688736	0.472	0.006	0.355	0.015	0.097	***0.706***
A2m	alpha-2-macroglobulin	0.471	0.009	0.608	0.002	0.469	0.033
RGD1310205	similar to RIKEN cDNA 4921508O11	0.469	0.007	0.112	***0.596***	0.200	***0.833***
Srsf11	serine/arginine-rich splicing factor 11	0.469	0.010	0.205	***0.367***	0.348	0.020
Tmprss7	transmembrane serine protease 7	0.469	0.010	-0.333	0.029	0.045	***0.346***
LOC686809	similar to protein 7 transactivated by hepatitis B virus X antigen	0.469	0.009	0.248	***0.869***	-0.287	***0.973***
Ace2	angiotensin I converting enzyme (peptidyl-dipeptidase A) 2	0.468	0.011	0.298	***0.988***	0.039	***0.612***
Homer2	homer homolog 2 (Drosophila)	0.468	0.005	0.005	***0.211***	0.290	0.030
Fam81a	family with sequence similarity 81, member A	0.468	0.017	0.171	***0.793***	0.034	***0.584***
RT1-T18	RT1 class Ib, locus T18	0.468	0.021	0.712	0.006	0.036	***0.557***
Fgd4	FYVE, RhoGEF and PH domain containing 4	0.467	0.008	0.091	***0.670***	0.120	***0.742***
LOC686388	similar to Prostatic steroid-binding protein C1 chain precursor (Prostatein peptide C1)	0.465	0.006	0.106	***0.419***	0.165	***0.701***
Rab20	RAB20, member RAS oncogene family	0.465	0.006	0.283	0.029	0.055	***0.514***
Dpy19l4	dpy-19-like 4 (C. elegans)	0.465	0.009	0.064	***0.766***	0.078	***0.735***
Vwf	von Willebrand factor	0.464	0.032	0.207	***0.852***	0.327	0.037
Dnaja4	DnaJ (Hsp40) homolog, subfamily A, member 4	0.463	0.009	0.220	***0.968***	0.339	0.027
RGD1566399	similar to MYST histone acetyltransferase monocytic leukemia 4	0.463	0.010	0.304	***0.883***	0.214	***0.943***
Bmp2k	BMP-2 inducible kinase	0.462	0.009	-0.001	***0.350***	0.359	0.020
LOC501437	similar to MIC2 like 1	0.461	0.009	0.008	***0.469***	0.202	***0.051***
Ahnak	AHNAK nucleoprotein	0.460	0.005	0.148	***0.523***	0.031	***0.390***
Bcl6	B-cell CLL/lymphoma 6	0.460	0.017	0.285	***0.053***	0.466	***0.050***
LOC100192205	heat shock factor binding protein 1-like	0.460	0.018	-0.137	***0.673***	-0.152	***0.884***
Siae	sialic acid acetylesterase	0.459	0.012	0.027	***0.656***	-0.095	***0.781***
RGD1560815	similar to acidic ribosomal phosphoprotein P1	0.459	0.011	0.156	***0.870***	0.046	***0.759***
Dll1	delta-like 1 (Drosophila)	0.459	0.012	0.070	***0.508***	-0.127	***0.826***
Kcnj1	potassium inwardly-rectifying channel, subfamily J, member 1	0.457	0.013	0.836	0.000	0.491	0.009
Stat4	signal transducer and activator of transcription 4	0.457	0.033	0.404	0.021	0.397	0.038
Plekhh1	pleckstrin homology domain containing, family H (with MyTH4 domain) member 1	0.456	0.008	0.662	0.000	-0.199	0.015
Creb1	cAMP responsive element binding protein 1	0.455	0.013	0.348	***0.984***	0.348	0.039
Nlrp5	NLR family, pyrin domain containing 5	0.454	0.012	0.077	***0.503***	0.116	***0.665***
St3gal2	ST3 beta-galactoside alpha-2,3-sialyltransferase 2	0.454	0.021	0.253	***0.808***	-0.026	***0.470***
Hgf	hepatocyte growth factor	0.453	0.005	0.466	0.005	0.259	0.029
Cited2	Cbp/p300-interacting transactivator, with Glu/Asp-rich carboxy-terminal domain, 2	0.452	0.012	0.094	***0.644***	0.143	***0.802***
Cpne8	copine VIII	0.452	0.016	0.093	***0.749***	0.101	***0.847***
Rnf145	ring finger protein 145	0.452	0.013	0.049	***0.610***	-0.026	***0.506***
Lax1	lymphocyte transmembrane adaptor 1	0.452	0.010	0.508	0.011	0.562	0.006
Map3k1	mitogen activated protein kinase kinase kinase 1	0.451	0.013	0.190	***0.532***	0.282	***0.055***

Ms4a1	membrane-spanning 4-domains, subfamily A, member	0.451	0.009	0.391	0.029	0.615	0.002
Minpp1	multiple inositol-polyphosphate phosphatase 1	0.450	0.010	0.372	0.019	0.027	***0.442***
Nr2f2	nuclear receptor subfamily 2, group F, member 2	0.450	0.007	0.299	***0.836***	0.130	***0.704***
Tmem194b	transmembrane protein 194B	0.450	0.015	0.462	0.009	0.380	0.039
Rgl3	ral guanine nucleotide dissociation stimulator-like 3	0.450	0.011	0.529	0.006	0.172	***0.873***
Agpat4	1-acylglycerol-3-phosphate O-acyltransferase 4 (lysophosphatidic acid acyltransferase, delta)	0.450	0.012	0.152	***0.743***	0.165	***0.899***
RT1-EC2	RT1 class Ib, locus EC2	0.450	0.017	0.300	0.025	0.362	0.016
Klhl24	kelch-like 24 (Drosophila)	0.449	0.010	0.336	***0.984***	0.487	0.004
Angpt2	angiopoietin 2	0.449	0.009	0.192	***0.900***	0.105	***0.594***
Sh3d19	SH3 domain containing 19	0.449	0.007	0.083	***0.477***	0.064	***0.462***
Nr5a2	nuclear receptor subfamily 5, group A, member 2	0.448	0.010	0.029	***0.579***	0.065	***0.611***
Hexim1	hexamethylene bis-acetamide inducible 1	0.447	0.011	0.176	***0.904***	0.109	***0.780***
LOC681325	hypothetical protein LOC681325	0.447	0.029	0.421	***0.065***	0.551	0.028
Itpkc	inositol-trisphosphate 3-kinase C	0.447	0.013	0.207	***0.931***	0.107	***0.854***
Clcn1	chloride channel 1	0.446	0.015	0.090	***0.688***	0.023	***0.650***
Ppcdc	phosphopantothenoylcysteine decarboxylase	0.446	0.025	0.362	***0.985***	0.159	***0.918***
Adam15	a disintegrin and metallopeptidase domain 15 (metargidin)	0.446	0.015	0.036	***0.541***	0.008	***0.588***
Ctnnal1	catenin (cadherin associated protein), alpha-like 1	0.446	0.012	0.101	***0.776***	0.192	***0.927***
Asb15	ankyrin repeat and SOCS box-containing protein 15	0.445	0.047	0.123	***0.864***	0.060	***0.797***
Iqgap1	IQ motif containing GTPase activating protein 1	0.445	0.002	0.106	0.042	0.108	0.033
Cdyl	chromodomain protein, Y-like	0.444	0.014	0.197	***0.951***	0.097	***0.822***
Fam129b	family with sequence similarity 129, member B	0.444	0.014	0.051	***0.613***	-0.077	***0.596***
Ccl9	chemokine (C-C motif) ligand 9	0.444	0.020	0.162	0.027	0.106	0.035
Ntsr2	neurotensin receptor 2	0.444	0.034	-0.032	***0.643***	-0.024	***0.544***
RGD1562136	similar to D1Ertd622e protein	0.444	0.012	-0.005	***0.492***	0.072	***0.743***
LOC691813	similar to phospholipase A2, group IVC (cytosolic, calcium-independent)	0.444	0.010	0.481	0.004	0.949	0.001
Sag	S-antigen; retina and pineal gland (arrestin)	0.443	0.033	0.379	***0.062***	0.384	0.028
Kctd11	potassium channel tetramerisation domain containing 11	0.442	0.012	0.136	***0.833***	0.216	***0.959***
Napepld	N-acyl phosphatidylethanolamine phospholipase D	0.442	0.012	0.030	***0.394***	-0.072	***0.391***
Sgk2	serum/glucocorticoid regulated kinase 2	0.442	0.002	-0.326	0.008	-0.029	0.031
Prdx5	peroxiredoxin 5	0.441	0.021	0.273	***0.972***	0.075	***0.745***
Snn	stannin	0.441	0.005	0.445	0.010	0.408	0.030
Tp53inp1	tumor protein p53 inducible nuclear protein 1	0.441	0.014	0.224	***0.920***	0.314	0.045
Tcp11	t-complex protein 11	0.440	0.011	0.443	0.015	-0.102	***0.360***
RT1-CE2	RT1 class I, locus CE2	0.440	0.013	0.400	0.011	0.407	0.011
Cytip	cytohesin 1 interacting protein	0.439	0.006	0.625	0.012	0.862	0.001
Gpr137b	G protein-coupled receptor 137B	0.439	0.019	-0.010	***0.476***	0.097	***0.661***
Nr3c2	nuclear receptor subfamily 3, group C, member 2	0.439	0.014	0.069	***0.533***	0.089	***0.780***
LOC681820	hypothetical protein LOC681820	0.439	0.017	0.172	***0.767***	-0.153	***0.866***
Itga6	integrin, alpha 6	0.439	0.012	-0.037	***0.336***	0.051	***0.407***
St6galnac1	ST6 (alpha-N-acetyl-neuraminyl-2,3-beta-galactosyl-1,3)-N-acetylgalactosaminide alpha-2,6-sialyltransferase 1	0.439	0.013	0.227	***0.938***	0.221	***0.794***
Ubxn7	UBX domain protein 7	0.439	0.016	0.288	***0.909***	0.401	0.020
Tns4	tensin 4	0.439	0.012	0.218	***0.907***	0.091	***0.497***
RGD1559532	RGD1559532	0.438	0.017	0.108	***0.738***	0.092	***0.645***
LOC684327	similar to inter-alpha (globulin) inhibitor H5	0.438	0.013	0.356	0.026	0.279	0.046
Itgb8	integrin, beta 8	0.438	0.012	0.116	***0.574***	0.105	***0.732***
Apln	apelin	0.437	0.021	-0.315	0.036	-0.708	0.000
Sh2b2	SH2B adaptor protein 2	0.437	0.015	0.656	0.006	0.604	0.007
Chst1	carbohydrate (keratan sulfate Gal-6) sulfotransferase 1	0.436	0.004	0.527	0.005	0.431	0.004
Tax1bp1	Tax1 (human T-cell leukemia virus type I) binding protein 1	0.436	0.015	0.389	0.022	0.406	0.016
LOC687346	similar to BRCA1-associated protein (BRAP2) (Impedes mitogenic signal propagation) (IMP)	0.436	0.013	0.204	***0.778***	-0.046	***0.620***
RT1-CE4	RT1 class I, locus CE4	0.436	0.027	0.367	0.026	0.334	0.034
Nr5a2	nuclear receptor subfamily 5, group A, member 2	0.436	0.012	-0.047	***0.657***	0.036	***0.560***
Pou2f1	POU class 2 homeobox 1	0.436	0.014	0.284	***0.922***	0.239	***0.674***
Sall2	sal-like 2 (Drosophila)	0.435	0.017	0.154	***0.840***	0.018	***0.719***
Phldb3	pleckstrin homology-like domain, family B, member 3	0.434	0.018	-0.069	***0.719***	0.007	***0.526***

Rhbg	Rh family, B glycoprotein	0.434	0.026	-0.005	***0.709***	-0.016	***0.667***
Neurod1	neurogenic differentiation 1	0.433	0.012	0.170	***0.808***	0.182	***0.873***
Exoc3l4	exocyst complex component 3-like 4	0.433	0.014	0.455	0.010	0.351	0.036
Gda	guanine deaminase	0.433	0.016	0.073	***0.418***	0.100	***0.490***
C1ql3	complement component 1, q subcomponent-like 3	0.433	0.006	0.467	0.009	0.324	***0.054***
Ccbl1	cysteine conjugate-beta lyase, cytoplasmic	0.433	0.018	0.270	***0.963***	0.186	***0.822***
Rnf186	ring finger protein 186	0.432	0.015	0.149	***0.774***	0.015	***0.585***
RGD1305704	similar to c-Mpl binding protein	0.431	0.033	-0.009	***0.470***	0.063	***0.645***
Lypd3	Ly6/Plaur domain containing 3	0.430	0.019	-0.048	***0.758***	-0.073	***0.808***
Dnajc5b	DnaJ (Hsp40) homolog, subfamily C, member 5 beta	0.430	0.046	0.227	***0.691***	-0.099	***0.763***
RT1-CE7	RT1 class I, locus CE7	0.429	0.012	0.162	***0.428***	0.067	***0.589***
Padi4	peptidyl arginine deiminase, type IV	0.428	0.020	0.295	***0.599***	-0.050	***0.470***
Phgdh	phosphoglycerate dehydrogenase	0.427	0.019	0.394	0.025	0.130	***0.685***
Sun2	Sad1 and UNC84 domain containing 2	0.427	0.016	0.436	0.011	0.429	0.010
Slc12a6	solute carrier family 12, member 6	0.427	0.013	0.168	***0.777***	0.126	***0.723***
RT1-A1	RT1 class Ia, locus A1	0.427	0.014	0.225	0.042	0.232	***0.902***
RGD1305899	similar to Protein C20orf158	0.427	0.018	-0.073	***0.685***	-0.234	***0.894***
Per3	period homolog 3 (Drosophila)	0.426	0.019	0.208	***0.958***	0.110	***0.874***
Irs1	insulin receptor substrate 1	0.426	0.020	0.194	***0.928***	-0.042	***0.701***
Pllp	plasmolipin	0.426	0.019	0.033	***0.735***	-0.057	***0.675***
Slc5a4a	solute carrier family 5 (low affinity glucose cotransporter), member 4a	0.426	0.011	0.073	***0.415***	0.243	0.033
Ankrd42	ankyrin repeat domain 42	0.425	0.020	0.033	***0.705***	0.007	***0.616***
Cyp2c6v1	cytochrome P450, family 2, subfamily C, polypeptide 6, variant 1	0.425	0.014	0.296	***0.794***	0.136	***0.751***
Myo1a	myosin IA	0.425	0.019	0.003	***0.635***	-0.047	***0.627***
Stfa2l1	stefin A2-like 1	0.424	0.024	0.457	0.021	-0.107	***0.654***
S1pr4	sphingosine-1-phosphate receptor 4	0.424	0.011	0.299	0.046	0.383	0.017
Pde9a	phosphodiesterase 9A	0.424	0.019	0.341	0.029	0.249	***0.921***
LOC680097	similar to germinal histone H4 gene	0.424	0.019	-0.209	***0.878***	-0.101	***0.652***
Cyp11b3	cytochrome P450, family 11, subfamily b, polypeptide 3	0.423	0.027	0.713	0.005	0.173	***0.878***
Ggh	gamma-glutamyl hydrolase (conjugase, folylpolygammaglutamyl hydrolase)	0.423	0.019	0.048	***0.686***	0.180	***0.858***
Med17	mediator complex subunit 17	0.423	0.009	0.227	0.038	0.125	***0.084***
Rmnd5a	required for meiotic nuclear division 5 homolog A (S. cerevisiae)	0.423	0.009	-0.139	***0.443***	0.096	***0.503***
Gpank1	G patch domain and ankyrin repeats 1	0.423	0.019	0.368	0.023	0.306	0.047
RGD1563562	similar to GTPase activating protein testicular GAP1	0.423	0.022	0.187	***0.592***	0.123	***0.579***
Ubxn2b	UBX domain protein 2B	0.423	0.034	0.111	***0.754***	-0.170	***0.945***
LOC683463	similar to paired-Ig-like receptor B	0.422	0.036	0.028	***0.584***	0.554	0.016
Wnt11	wingless-type MMTV integration site family, member 11	0.422	0.012	0.012	***0.450***	0.047	***0.487***
RGD1565164	similar to associated molecule with the SH3 domain of STAM	0.422	0.027	-0.016	***0.681***	0.018	***0.646***
Kitlg	KIT ligand	0.421	0.019	0.043	***0.692***	0.001	***0.569***
Igsf3	immunoglobulin superfamily, member 3	0.421	0.017	0.157	***0.797***	0.035	***0.723***
RGD1305679	similar to 9530008L14Rik protein	0.421	0.019	0.041	***0.706***	0.289	***0.053***
Casr	calcium-sensing receptor	0.420	0.017	0.246	***0.875***	0.106	***0.656***
Tm4sf20	transmembrane 4 L six family member 20	0.420	0.006	0.359	0.007	0.143	***0.051***
Vom1r22	vomeronasal 1 receptor 22	0.420	0.027	0.154	***0.711***	0.254	***0.958***
Trim36	tripartite motif-containing 36	0.418	0.018	0.195	***0.657***	0.098	***0.830***
Aldh1l1	aldehyde dehydrogenase 1 family, member L1	0.417	0.019	0.185	***0.810***	0.015	***0.601***
Tcp11l2	t-complex 11 (mouse) like 2	0.417	0.014	0.210	***0.887***	0.323	0.029
Ahnak	AHNAK nucleoprotein	0.417	0.023	0.219	***0.465***	0.019	***0.448***
Casc5	cancer susceptibility candidate 5	0.417	0.018	-0.086	***0.611***	-0.038	***0.448***
Clcn5	chloride channel 5	0.416	0.023	0.080	***0.368***	0.180	***0.843***
RT1-Db1	RT1 class II, locus Db1	0.416	0.007	0.479	0.001	0.682	0.000
Arap2	ArfGAP with RhoGAP domain, ankyrin repeat and PH domain 2	0.416	0.019	0.274	***0.919***	0.418	0.010
Mtfr1	mitochondrial fission regulator 1	0.414	0.035	0.055	***0.642***	-0.133	***0.818***
Nr1h4	nuclear receptor subfamily 1, group H, member 4	0.414	0.019	0.086	***0.530***	-0.067	***0.719***
Klf9	Kruppel-like factor 9	0.414	0.018	0.364	0.026	0.282	***0.053***
Rhov	ras homolog gene family, member V	0.414	0.011	-0.394	0.007	-0.177	***0.594***
Spint1	serine peptidase inhibitor, Kunitz type 1	0.414	0.019	0.037	***0.484***	0.178	***0.951***
Gimap7	GTPase, IMAP family member 7	0.413	0.027	0.418	0.018	0.815	0.002

Il1rl1	interleukin 1 receptor-like 1	0.413	0.013	0.339	0.016	0.378	0.011
Arhgap39	Rho GTPase activating protein 39	0.413	0.023	0.243	***0.948***	0.266	***0.983***
Clec14a	C-type lectin domain family 14, member A	0.413	0.020	0.269	***0.943***	0.073	***0.689***
Cbp	Csk binding protein	0.413	0.017	0.071	***0.685***	0.188	***0.873***
Ptprr	protein tyrosine phosphatase, receptor type, R	0.412	0.023	0.065	***0.561***	-0.264	***0.912***
Bmp2	bone morphogenetic protein 2	0.412	0.021	0.213	***0.908***	0.133	***0.876***
Slc5a1	solute carrier family 5 (sodium/glucose cotransporter), member 1	0.412	0.017	0.206	***0.835***	0.013	***0.607***
Aqp7	aquaporin 7	0.411	0.017	0.133	***0.104***	0.176	***0.065***
Cs	citrate synthase	0.411	0.020	0.278	***0.962***	0.180	***0.929***
Ahdc1	AT hook, DNA binding motif, containing 1	0.410	0.024	0.201	***0.720***	0.252	***0.795***
Prelp	proline/arginine-rich end leucine-rich repeat protein	0.410	0.019	0.205	***0.876***	-0.002	***0.514***
Il1r2	interleukin 1 receptor, type II	0.410	0.019	0.486	0.006	0.353	0.044
Hook3	hook homolog 3 (Drosophila)	0.410	0.018	0.282	0.036	0.226	0.043
Zfp608	zinc finger protein 608	0.410	0.020	0.081	***0.831***	0.222	***0.968***
Htra4	HtrA serine peptidase 4	0.410	0.035	0.172	***0.668***	-0.068	***0.657***
Siae	sialic acid acetylesterase	0.410	0.021	0.067	***0.694***	-0.019	***0.563***
RGD1561115	similar to Gene model 1568	0.409	0.026	0.182	***0.428***	0.008	***0.374***
Rreb1	ras responsive element binding protein 1	0.409	0.017	-0.095	***0.464***	-0.073	***0.466***
Ugt2b34	UDP glucuronosyltransferase 2 family, polypeptide B34	0.409	0.017	0.101	***0.468***	0.093	***0.419***
Klf10	Kruppel-like factor 10	0.409	0.015	0.113	***0.656***	0.020	***0.408***
Tnrc6b	trinucleotide repeat containing 6B	0.408	0.024	0.194	***0.807***	0.114	***0.850***
Sit1	signaling threshold regulating transmembrane adaptor 1	0.408	0.003	0.533	0.002	0.658	0.001
Cad	carbamoyl-phosphate synthetase 2, aspartate transcarbamylase, and dihydroorotase	0.408	0.021	0.330	***0.898***	0.119	***0.643***
Sema6c	sema domain, transmembrane domain (TM), and cytoplasmic domain, (semaphorin) 6C	0.408	0.017	0.081	***0.763***	0.141	***0.746***
Snx25	sorting nexin 25	0.408	0.019	0.133	***0.791***	0.153	***0.798***
Aph1b	anterior pharynx defective 1 homolog B (C. elegans)	0.408	0.022	0.218	***0.969***	0.080	***0.760***
Enpp4	ectonucleotide pyrophosphatase/phosphodiesterase 4	0.408	0.012	0.028	***0.232***	0.075	***0.527***
Zfp133	zinc finger protein 133	0.408	0.024	0.033	***0.625***	0.002	***0.507***
Met	met proto-oncogene	0.407	0.019	-0.092	***0.763***	-0.014	***0.587***
Sesn1	sestrin 1	0.407	0.022	0.220	***0.932***	0.453	0.007
Gan	gigaxonin	0.407	0.028	0.152	***0.727***	-0.008	***0.490***
Phf3	PHD finger protein 3	0.406	0.023	0.189	***0.947***	0.099	***0.893***
Gtlf3b	gene trap locus F3b	0.405	0.015	0.265	***0.907***	0.280	0.048
RGD1562351	similar to chromosome 7 open reading frame 23	0.405	0.033	0.116	***0.145***	0.375	0.044
Cobl	cordon-bleu homolog (mouse)	0.405	0.016	-0.043	***0.631***	-0.032	***0.508***
Pdcd6ip	programmed cell death 6 interacting protein	0.405	0.013	0.082	***0.546***	0.303	0.026
S100vp	S100 calcium-binding protein, ventral prostate	0.405	0.024	0.102	***0.810***	0.102	***0.543***
Bahd1	bromo adjacent homology domain containing 1	0.404	0.025	0.027	***0.678***	-0.016	***0.597***
Sptssa	serine palmitoyltransferase, small subunit A	0.404	0.030	0.055	***0.731***	-0.183	***0.948***
Olr791	olfactory receptor 791	0.404	0.031	0.069	***0.790***	-0.017	***0.613***
Gclc	glutamate-cysteine ligase, catalytic subunit	0.403	0.017	0.116	***0.657***	-0.007	***0.365***
Gtf3c4	general transcription factor IIIC, polypeptide 4	0.403	0.028	0.214	***0.604***	-0.023	***0.675***
Ehbp1	EH domain binding protein 1	0.403	0.021	0.194	***0.853***	0.290	***0.954***
Wasl	Wiskott-Aldrich syndrome-like	0.403	0.019	0.196	***0.901***	0.294	0.045
Trim16	tripartite motif-containing 16	0.402	0.021	-0.043	***0.535***	0.004	***0.543***
Ddit4l	DNA-damage-inducible transcript 4-like	0.402	0.012	0.147	***0.335***	0.311	0.031
Espnl	espin-like	0.402	0.029	0.251	***0.834***	0.006	***0.712***
Gpsm2	G-protein signaling modulator 2	0.402	0.023	-0.010	***0.610***	-0.144	***0.889***
Hist1h1a	histone cluster 1, H1a	0.402	0.009	0.118	***0.667***	0.011	***0.437***
Adrb2	adrenergic, beta-2-, receptor, surface	0.401	0.035	0.165	***0.695***	0.153	***0.342***
RGD1561149	similar to mKIAA1522 protein	0.401	0.023	0.073	***0.682***	-0.049	***0.644***
Marcks	myristoylated alanine rich protein kinase C substrate	0.401	0.020	0.120	***0.556***	-0.077	***0.660***
Cdcp2	CUB domain containing protein 2	0.400	0.025	0.682	0.008	0.096	***0.468***
Clcnkb	chloride channel Kb	0.400	0.015	0.359	0.018	-0.083	***0.545***
Pde10a	phosphodiesterase 10A	0.400	0.020	0.406	0.020	0.170	***0.910***
Tmem30b	transmembrane protein 30B	0.400	0.026	-0.130	***0.780***	-0.139	***0.822***
Ptprf	protein tyrosine phosphatase, receptor type, F	0.399	0.019	0.014	***0.329***	-0.034	***0.438***
Klhl25	kelch-like 25 (Drosophila)	0.399	0.024	0.065	***0.713***	-0.018	***0.573***

Cd3d	CD3 molecule, delta	0.398	0.018	0.460	0.014	0.731	0.004
Bmp6	bone morphogenetic protein 6	0.398	0.017	0.187	***0.605***	0.051	***0.687***
Apaf1	apoptotic peptidase activating factor 1	0.398	0.015	0.187	***0.528***	0.140	***0.544***
Pc	pyruvate carboxylase	0.398	0.023	0.193	***0.761***	0.078	***0.669***
Abcc3	ATP-binding cassette, subfamily C (CFTR/MRP), member 3	0.398	0.018	0.154	***0.813***	0.093	***0.591***
Arrdc2	arrestin domain containing 2	0.398	0.025	0.279	***0.949***	0.379	0.019
Hbp1	HMG-box transcription factor 1	0.397	0.014	0.151	***0.732***	0.087	***0.701***
Rabgap1	RAB GTPase activating protein 1	0.397	0.022	0.349	0.028	0.524	0.002
RGD1309058	similar to RIKEN cDNA 9830169C18	0.397	0.023	0.152	***0.862***	-0.032	***0.581***
Slc16a1	solute carrier family 16, member 1 (monocarboxylic acid transporter 1)	0.397	0.023	-0.002	***0.495***	0.019	***0.499***
Stradb	STE20-related kinase adaptor beta	0.396	0.026	0.089	***0.806***	0.043	***0.740***
LOC301725	similar to 60S ribosomal protein L35	0.395	0.027	0.297	***0.977***	0.158	***0.782***
LOC679811	similar to RIKEN cDNA D930015E06	0.395	0.026	0.292	***0.795***	0.158	***0.564***
Mtus1	microtubule associated tumor suppressor 1	0.395	0.021	0.187	***0.794***	0.128	***0.881***
Rab40b	Rab40b, member RAS oncogene family	0.395	0.034	0.083	***0.746***	-0.040	***0.685***
LOC678708	similar to histone 1, H2ai	0.395	0.025	0.023	***0.682***	0.028	***0.629***
Aph1b	anterior pharynx defective 1 homolog B (C. elegans)	0.395	0.021	0.192	***0.947***	0.089	***0.798***
LOC301816	similar to Spindlin-like protein 2 (SPIN-2)	0.394	0.035	0.066	***0.481***	-0.046	***0.200***
Gchfr	GTP cyclohydrolase I feedback regulator	0.394	0.019	0.138	***0.684***	-0.041	***0.359***
Idh1	isocitrate dehydrogenase 1 (NADP+), soluble	0.394	0.026	-0.026	***0.608***	0.079	***0.516***
Klhdc5	kelch domain containing 5	0.394	0.013	-0.022	***0.429***	0.066	***0.624***
Ap2b1	adaptor-related protein complex 2, beta 1 subunit	0.394	0.010	0.287	0.022	0.167	***0.055***
Neo1	neogenin 1	0.393	0.025	0.167	***0.898***	0.097	***0.851***
Phf13	PHD finger protein 13	0.393	0.028	0.145	***0.794***	-0.178	***0.846***
LOC690137	similar to transmembrane protein induced by tumor necrosis factor alpha	0.393	0.019	0.146	***0.581***	0.159	***0.588***
Cep68	centrosomal protein 68	0.393	0.024	0.347	0.029	0.203	***0.950***
Plcxd2	phosphatidylinositol-specific phospholipase C, X domain containing 2	0.393	0.018	-0.072	***0.485***	-0.100	***0.465***
Dsp	desmoplakin	0.392	0.019	0.291	***0.985***	0.352	0.019
Tcf7l2	transcription factor 7-like 2 (T-cell specific, HMG-box)	0.392	0.031	0.014	***0.396***	0.176	***0.910***
Kcna7	potassium voltage-gated channel, shaker-related subfamily, member 7	0.392	0.027	0.120	***0.814***	0.107	***0.768***
Chad	chondroadherin	0.392	0.019	0.309	0.033	0.163	***0.100***
Krtap13-2	keratin associated protein 13-2	0.392	0.009	-0.409	0.004	-0.010	***0.136***
Rfx5	regulatory factor X, 5 (influences HLA class II expression)	0.391	0.023	0.900	0.001	0.231	0.038
Dync1i1	dynein cytoplasmic 1 intermediate chain 1	0.391	0.010	0.145	0.029	-0.260	***0.452***
Amn	amnionless homolog (mouse)	0.391	0.011	-0.163	***0.470***	-0.315	0.017
Sorl1	sortilin-related receptor, LDLR class A repeats-containing	0.391	0.025	0.362	***0.895***	0.194	***0.828***
RGD1310262	hypothetical LOC304650	0.391	0.027	-0.202	***0.796***	-0.321	0.019
Pclo	piccolo (presynaptic cytomatrix protein)	0.391	0.028	0.184	***0.639***	-0.111	***0.778***
Zc3h13	zinc finger CCCH type containing 13	0.390	0.013	0.519	0.002	0.623	0.000
Trib3	tribbles homolog 3 (Drosophila)	0.390	0.013	-0.088	0.047	0.045	***0.522***
Pmaip1	phorbol-12-myristate-13-acetate-induced protein 1	0.389	0.018	0.197	***0.819***	0.150	***0.785***
Clrn2	clarin 2	0.389	0.036	0.072	***0.803***	0.108	***0.860***
Sft2d2	SFT2 domain containing 2	0.388	0.018	-0.074	***0.596***	0.005	***0.597***
Fam55c	family with sequence similarity 55, member C	0.388	0.022	0.362	0.031	0.429	0.015
Iqgap1	IQ motif containing GTPase activating protein 1	0.388	0.021	0.156	***0.716***	0.027	***0.606***
Tepp	testis, prostate and placenta expressed	0.388	0.025	0.661	0.001	0.252	***0.843***
Xiap	X-linked inhibitor of apoptosis	0.388	0.028	0.039	***0.450***	0.152	***0.798***
Ankib1	ankyrin repeat and IBR domain containing 1	0.388	0.024	0.055	***0.401***	0.023	***0.492***
Lpo	lactoperoxidase	0.388	0.022	0.089	***0.697***	0.015	***0.476***
LOC683498	similar to Actin, aortic smooth muscle (Alpha-actin-2)	0.388	0.030	0.003	***0.576***	-0.034	***0.620***
Scn4b	sodium channel, voltage-gated, type IV, beta	0.388	0.030	0.314	0.020	-0.178	***0.448***
Car4	carbonic anhydrase 4	0.388	0.015	0.345	0.015	0.293	0.015
Gadd45a	growth arrest and DNA-damage-inducible, alpha	0.387	0.034	0.106	***0.852***	0.133	***0.873***
Fam193a	family with sequence similarity 193, member A	0.387	0.029	0.438	0.014	0.354	0.035
Zcchc11	zinc finger, CCHC domain containing 11	0.387	0.031	0.123	***0.915***	-0.029	***0.695***
Plk1s1	polo-like kinase 1 substrate 1	0.386	0.026	0.321	***0.982***	0.307	0.045

Upf2	UPF2 regulator of nonsense transcripts homolog (yeast)	0.386	0.032	0.024	***0.679***	0.057	***0.793***
Exph5	exophilin 5	0.386	0.039	0.012	***0.516***	0.019	***0.588***
Lmo4	LIM domain only 4	0.386	0.024	0.189	***0.871***	0.220	***0.928***
RT1-S3	RT1 class Ib, locus S3	0.386	0.030	0.247	0.036	0.378	0.016
Zfp217	zinc finger protein 217	0.386	0.020	0.005	***0.397***	-0.092	***0.430***
Gpsm1	G-protein signaling modulator 1	0.385	0.022	0.002	***0.492***	0.133	***0.725***
RGD1563680	similar to CDNA sequence BC052040	0.385	0.030	0.273	***0.778***	0.143	***0.887***
RGD1308138	similar to hypothetical protein LOC131368	0.385	0.007	-0.199	0.046	-0.905	0.003
Sf3a1	splicing factor 3a, subunit 1	0.385	0.031	0.086	***0.820***	0.098	***0.685***
Aqp11	aquaporin 11	0.385	0.024	-0.001	***0.481***	-0.172	***0.791***
Synj2	synaptojanin 2	0.385	0.031	0.164	***0.705***	0.141	***0.906***
Vdr	vitamin D (1,25- dihydroxyvitamin D3) receptor	0.384	0.029	-0.074	***0.667***	-0.038	***0.503***
Cd79b	Cd79b molecule, immunoglobulin-associated beta	0.384	0.001	0.626	0.001	0.694	0.000
Cnnm4	cyclin M4	0.384	0.013	-0.122	***0.570***	0.107	***0.553***
Mpp5	membrane protein, palmitoylated 5 (MAGUK p55 subfamily member 5)	0.384	0.033	0.100	***0.589***	0.108	***0.836***
Pla2g2a	phospholipase A2, group IIA (platelets, synovial fluid)	0.383	0.004	0.213	0.014	-0.225	0.008
Prc1	protein regulator of cytokinesis 1	0.383	0.030	-0.010	***0.694***	0.016	***0.623***
Irs1	insulin receptor substrate 1	0.383	0.022	0.338	0.033	0.067	***0.624***
Hist1h3f	histone cluster 1, H3f	0.383	0.024	-0.089	***0.674***	-0.035	***0.416***
Mtus1	microtubule associated tumor suppressor 1	0.383	0.029	0.152	***0.847***	-0.039	***0.532***
Tob2	transducer of ERBB2, 2	0.383	0.023	0.154	***0.793***	-0.045	***0.455***
Mfap3l	microfibrillar-associated protein 3-like	0.382	0.033	-0.082	***0.822***	-0.017	***0.558***
Fgd4	FYVE, RhoGEF and PH domain containing 4	0.382	0.026	0.043	***0.375***	-0.018	***0.581***
Klf5	Kruppel-like factor 5	0.382	0.030	-0.035	***0.556***	0.030	***0.383***
Nr1d1	nuclear receptor subfamily 1, group D, member 1	0.382	0.020	0.285	0.023	1.336	0.000
Phlpp1	PH domain and leucine rich repeat protein phosphatase 1	0.381	0.031	0.220	***0.965***	0.137	***0.870***
Kcnq1	potassium voltage-gated channel, KQT-like subfamily, member 1	0.381	0.032	0.122	***0.818***	-0.013	***0.609***
Olr1261	olfactory receptor 1261	0.381	0.020	0.221	***0.752***	0.135	***0.824***
Fam117b	family with sequence similarity 117, member B	0.381	0.032	0.159	***0.926***	0.177	***0.940***
LOC500846	hypothetical protein LOC500846	0.381	0.023	0.475	0.006	0.445	0.005
Lifr	leukemia inhibitory factor receptor alpha	0.381	0.015	0.493	0.005	0.573	0.002
Mphosph9	M-phase phosphoprotein 9	0.381	0.031	0.147	***0.841***	0.199	***0.949***
Olr428	olfactory receptor 428	0.381	0.036	0.087	***0.591***	-0.176	***0.947***
Usp24	ubiquitin specific protease 24	0.380	0.020	0.247	***0.754***	0.316	0.039
Rsl1d1	ribosomal L1 domain containing 1	0.380	0.028	0.222	***0.697***	0.031	***0.562***
Plekhf2	pleckstrin homology domain containing, family F (with FYVE domain) member 2	0.380	0.013	0.093	***0.484***	0.015	***0.348***
Pdlim5	PDZ and LIM domain 5	0.379	0.023	0.104	***0.260***	0.215	***0.690***
Crebl2	cAMP responsive element binding protein-like 2	0.379	0.022	0.057	***0.459***	0.071	***0.625***
Klhl14	kelch-like 14 (Drosophila)	0.379	0.046	0.104	0.041	0.268	***0.068***
Ptbp3	polypyrimidine tract binding protein 3	0.378	0.031	0.007	***0.481***	0.147	***0.850***
Trove2	TROVE domain family, member 2	0.378	0.030	0.043	***0.735***	0.060	***0.763***
Olr1145	olfactory receptor 1145	0.378	0.032	0.059	***0.611***	-0.069	***0.757***
Mfhas1	malignant fibrous histiocytoma amplified sequence 1	0.377	0.034	0.384	0.019	0.247	***0.958***
Plekhf2	pleckstrin homology domain containing, family F (with FYVE domain) member 2	0.377	0.031	0.084	***0.640***	-0.006	***0.509***
Fam100b	family with sequence similarity 100, member B	0.377	0.034	0.123	***0.889***	0.048	***0.780***
LOC687808	similar to solute carrier family 16 (monocarboxylic acid transporters), member 9	0.376	0.017	0.188	***0.840***	0.122	***0.686***
C1galt1	core 1 synthase, glycoprotein-N-acetylgalactosamine 3-beta-galactosyltransferase, 1	0.376	0.031	-0.145	***0.831***	0.037	***0.640***
LOC683422	similar to Plasma kallikrein precursor (Plasma prekallikrein) (Kininogenin) (Fletcher factor)	0.376	0.034	0.516	0.005	0.457	0.011
Sort1	sortilin 1	0.376	0.031	0.082	***0.774***	-0.078	***0.762***
Camk4	calcium/calmodulin-dependent protein kinase IV	0.376	0.042	0.326	***0.484***	0.600	0.011
Ezh1	enhancer of zeste homolog 1 (Drosophila)	0.375	0.031	0.162	***0.933***	0.096	***0.833***
Alms1	Alstrom syndrome 1 homolog (human)	0.375	0.039	0.200	***0.851***	0.241	***0.949***
Ggt7	gamma-glutamyltransferase 7	0.375	0.032	0.197	***0.892***	0.060	***0.704***
Paqr5	progestin and adipoQ receptor family member V	0.375	0.027	-0.069	***0.580***	-0.143	***0.662***

Cyp2d4	cytochrome P450, family 2, subfamily d, polypeptide 4	0.375	0.035	0.093	***0.846***	-0.138	***0.882***
Tnrc6b	trinucleotide repeat containing 6B	0.375	0.020	0.097	***0.481***	0.172	***0.749***
Lrrc8a	leucine rich repeat containing 8 family, member A	0.374	0.031	0.003	***0.416***	-0.099	***0.557***
Olr546	olfactory receptor 546	0.374	0.025	0.118	***0.754***	0.039	***0.549***
Tmem164	transmembrane protein 164	0.374	0.025	0.086	***0.517***	0.068	***0.557***
LOC498236	LRRGT00186	0.373	0.032	0.414	0.020	0.237	***0.928***
RGD1310788	similar to RIKEN cDNA 0610039P13	0.373	0.006	0.298	0.011	0.406	0.004
Crlf1	cytokine receptor-like factor 1	0.373	0.039	-0.195	***0.838***	-0.321	***0.944***
RGD1561416	similar to novel protein (HT036)	0.373	0.028	-0.047	***0.506***	-0.104	***0.627***
Rap2a	RAS related protein 2a	0.373	0.025	0.051	***0.695***	0.081	***0.754***
RGD1564712	RGD1564712	0.372	0.024	0.269	0.031	0.230	0.040
Atoh8	atonal homolog 8 (Drosophila)	0.372	0.039	0.168	***0.906***	-0.034	***0.671***
Cck	cholecystokinin	0.372	0.030	0.005	***0.427***	0.118	***0.771***
Abhd2	abhydrolase domain containing 2	0.371	0.029	0.001	***0.395***	0.201	***0.846***
Slc15a4	solute carrier family 15, member 4	0.371	0.024	0.297	***0.916***	0.239	***0.864***
Tesk2	testis-specific kinase 2	0.371	0.028	0.009	***0.595***	-0.056	***0.643***
Abhd1	abhydrolase domain containing 1	0.371	0.033	0.134	***0.852***	-0.103	***0.841***
Insr	insulin receptor	0.371	0.034	-0.105	***0.685***	-0.188	***0.933***
E2f1	E2F transcription factor 1	0.370	0.027	0.192	***0.906***	-0.049	***0.603***
RT1-CE3	RT1 class I, locus CE3	0.370	0.047	0.333	***0.942***	0.295	***0.991***
Nfrkb	nuclear factor related to kappa B binding protein	0.370	0.040	0.308	***0.952***	0.328	0.050
LOC312502	similar to RAB11 family interacting protein 5 (class I) isoform 1	0.370	0.020	0.134	***0.608***	-0.112	***0.465***
Sp4	Sp4 transcription factor	0.370	0.040	0.079	***0.659***	0.012	***0.436***
Cttn	cortactin	0.369	0.044	0.033	***0.344***	-0.027	***0.462***
Lamp3	lysosomal-associated membrane protein 3	0.369	0.020	0.307	***0.696***	0.371	***0.058***
Tmem102	transmembrane protein 102	0.369	0.026	0.015	***0.387***	-0.132	***0.714***
Ripk4	receptor-interacting serine-threonine kinase 4	0.369	0.047	-0.070	***0.683***	-0.283	***0.990***
Pex11a	peroxisomal biogenesis factor 11 alpha	0.369	0.019	0.078	***0.619***	-0.067	***0.542***
Foxk1	forkhead box K1	0.368	0.043	0.297	***0.979***	0.337	0.040
Per3	period homolog 3 (Drosophila)	0.368	0.035	0.077	***0.766***	-0.043	***0.653***
Slk	STE20-like kinase	0.368	0.034	0.189	***0.902***	0.166	***0.869***
Tnik	TRAF2 and NCK interacting kinase	0.368	0.029	0.300	***0.927***	0.310	0.035
Stard13	StAR-related lipid transfer (START) domain containing 13	0.367	0.042	0.241	***0.897***	0.106	***0.872***
Inadl2	InaD-like 2 (Drosophila)	0.367	0.037	0.003	***0.652***	-0.017	***0.630***
Kdm1b	lysine (K)-specific demethylase 1B	0.367	0.037	0.091	***0.786***	0.171	***0.845***
Sectm1b	secreted and transmembrane 1B	0.367	0.010	-0.473	0.007	-0.406	0.010
RGD1563169	similar to hypothetical protein 4930474N05	0.367	0.025	0.071	***0.677***	0.063	***0.568***
Cd27	CD27 molecule	0.366	0.005	0.924	0.001	1.006	0.000
Sfxn5	sideroflexin 5	0.366	0.036	0.100	***0.710***	0.022	***0.695***
Spred2	sprouty-related, EVH1 domain containing 2	0.365	0.038	0.152	***0.894***	0.115	***0.833***
Vill	villin-like	0.365	0.035	0.220	***0.866***	0.098	***0.658***
Pdcd4	programmed cell death 4	0.365	0.034	0.068	***0.709***	0.027	***0.560***
Pdp1	pyruvate dehyrogenase phosphatase catalytic subunit 1	0.365	0.036	0.403	0.027	0.194	***0.939***
Adra1b	adrenergic, alpha-1B-, receptor	0.365	0.028	0.189	***0.800***	-0.091	***0.720***
Tas2r120	taste receptor, type 2, member 120	0.365	0.046	0.286	***0.837***	-0.066	***0.656***
Tmco4	transmembrane and coiled-coil domains 4	0.365	0.037	-0.032	***0.623***	-0.106	***0.756***
Ctdspl2	CTD (carboxy-terminal domain, RNA polymerase II, polypeptide A) small phosphatase like 2	0.364	0.021	0.053	***0.561***	0.047	***0.610***
Grb7	growth factor receptor bound protein 7	0.364	0.035	0.154	***0.432***	0.080	***0.835***
Olr1421	olfactory receptor 1421	0.364	0.004	-0.361	***0.055***	-0.534	0.015
LOC501110	similar to Glutathione S-transferase A1 (GTH1) (HA subunit 1) (GST-epsilon) (GSTA1-1) (GST class-alpha)	0.364	0.007	0.505	0.004	0.438	0.004
Neo1	neogenin 1	0.364	0.033	0.149	***0.870***	0.060	***0.741***
RGD1564961	similar to retinoblastoma-binding protein 1 isoform I	0.364	0.041	-0.001	***0.558***	0.127	***0.787***
Fam211a	family with sequence similarity 211, member A	0.363	0.041	0.084	***0.751***	-0.013	***0.533***
Rnf216	ring finger protein 216	0.363	0.042	0.267	***0.933***	0.153	***0.933***
Tmem231	transmembrane protein 231	0.363	0.031	0.160	***0.706***	0.079	***0.450***
Capza1	capping protein (actin filament) muscle Z-line, alpha 1	0.363	0.021	0.108	***0.450***	0.101	***0.410***
Lamc2	laminin, gamma 2	0.362	0.033	0.149	***0.727***	0.154	***0.866***
Prf1	perforin 1 (pore forming protein)	0.362	0.028	0.532	0.011	0.717	0.002

RGD1308878	similar to arylacetamide deacetylase	0.362	0.018	0.269	***0.782***	0.281	0.026
Clip1	CAP-GLY domain containing linker protein 1	0.362	0.039	0.085	***0.809***	0.078	***0.831***
Nrgn	neurogranin	0.362	0.028	0.152	***0.596***	-0.037	***0.459***
RGD1562037	similar to OTTHUMP00000046255	0.362	0.044	0.275	***0.935***	-0.088	***0.876***
Atp7a	ATPase, Cu++ transporting, alpha polypeptide	0.362	0.035	0.093	***0.768***	0.121	***0.861***
Fbxl8	F-box and leucine-rich repeat protein 8	0.361	0.043	0.026	***0.658***	0.038	***0.500***
Esm1	endothelial cell-specific molecule 1	0.361	0.019	-0.266	***0.733***	-0.578	0.001
Maml1	mastermind like 1 (Drosophila)	0.361	0.038	0.093	***0.801***	0.134	***0.756***
Ednrb	endothelin receptor type B	0.361	0.023	0.136	***0.772***	0.024	***0.392***
Rtf1	Rtf1, Paf1/RNA polymerase II complex component, homolog (S. cerevisiae)	0.360	0.034	0.275	***0.975***	0.327	0.038
Ccrn4l	CCR4 carbon catabolite repression 4-like (S. cerevisiae)	0.360	0.042	0.098	***0.715***	-0.065	***0.674***
Arhgap35	Rho GTPase activating protein 35	0.360	0.042	0.214	***0.840***	0.193	***0.654***
Wfdc3	WAP four-disulfide core domain 3	0.360	0.019	-0.128	***0.492***	0.013	***0.181***
Lrp4	low density lipoprotein receptor-related protein 4	0.360	0.035	0.224	***0.847***	0.047	***0.634***
Ppapdc2	phosphatidic acid phosphatase type 2 domain containing 2	0.360	0.049	0.040	***0.769***	0.003	***0.668***
Ficd	FIC domain containing	0.360	0.028	0.110	***0.621***	0.027	***0.497***
Atoh8	atonal homolog 8 (Drosophila)	0.359	0.041	0.165	***0.882***	-0.101	***0.762***
Pfas	phosphoribosylformylglycinamidine synthase	0.359	0.034	0.126	***0.863***	0.101	***0.873***
Grid2ip	glutamate receptor, ionotropic, delta 2 (Grid2) interacting protein	0.359	0.016	-0.107	***0.389***	-0.234	***0.648***
RGD1564964	similar to WD repeat domain 11 protein	0.359	0.022	0.181	***0.483***	0.286	0.033
Tspan33	tetraspanin 33	0.358	0.018	0.147	***0.564***	0.046	***0.388***
Arx	aristaless related homeobox	0.358	0.043	0.218	***0.952***	0.129	***0.897***
Sp4	Sp4 transcription factor	0.358	0.046	-0.020	***0.588***	-0.045	***0.584***
Clic5	chloride intracellular channel 5	0.358	0.028	0.103	***0.709***	0.029	***0.297***
Trim23	tripartite motif-containing 23	0.358	0.030	0.186	***0.889***	0.304	0.038
aicda	activation-induced cytidine deaminase	0.357	0.046	0.446	0.020	0.733	0.007
Inpp4b	inositol polyphosphate-4-phosphatase, type II	0.357	0.028	0.260	***0.471***	0.382	0.021
Slco6d1	solute carrier organic anion transporter family, member 6d1	0.357	0.029	-0.008	***0.447***	-0.105	***0.514***
Cables1	Cdk5 and Abl enzyme substrate 1	0.356	0.041	0.008	***0.586***	-0.179	***0.960***
Pigv	phosphatidylinositol glycan anchor biosynthesis, class V	0.356	0.041	0.106	***0.773***	0.117	***0.867***
Akap13	A kinase (PRKA) anchor protein 13	0.356	0.042	0.344	***0.980***	0.235	***0.813***
Syt12	synaptotagmin XII	0.356	0.040	0.317	***0.991***	0.123	***0.893***
Rab32	RAB32, member RAS oncogene family	0.355	0.041	0.113	***0.672***	0.192	***0.873***
Cbl	Cas-Br-M (murine) ecotropic retroviral transforming sequence	0.355	0.037	0.228	***0.939***	0.301	***0.055***
LOC100359930	Cyp2s1 protein-like	0.355	0.040	0.206	***0.873***	0.064	***0.651***
Odf3l1	outer dense fiber of sperm tails 3-like 1	0.355	0.034	0.163	***0.893***	-0.173	***0.916***
Tmco4	transmembrane and coiled-coil domains 4	0.355	0.041	0.095	***0.781***	0.010	***0.658***
LOC100363229	hypothetical protein LOC100363229	0.355	0.032	0.485	0.006	0.452	0.006
Atp1b1	ATPase, Na+/K+ transporting, beta 1 polypeptide	0.354	0.029	-0.002	***0.404***	-0.097	***0.589***
Rchy1	ring finger and CHY zinc finger domain containing 1	0.354	0.035	0.001	***0.484***	-0.043	***0.624***
Avpr1b	arginine vasopressin receptor 1B	0.354	0.044	0.106	***0.807***	0.016	***0.649***
Spns2	spinster homolog 2	0.354	0.041	0.084	***0.731***	-0.197	***0.842***
Gna14	guanine nucleotide binding protein, alpha 14	0.353	0.025	0.046	***0.565***	0.169	***0.752***
Cables1	Cdk5 and Abl enzyme substrate 1	0.353	0.039	-0.110	***0.815***	-0.211	***0.939***
Marveld3	MARVEL domain containing 3	0.352	0.039	0.077	***0.732***	0.119	***0.833***
Fam134b	family with sequence similarity 134, member B	0.352	0.042	0.081	***0.780***	0.200	***0.876***
Zcchc2	zinc finger, CCHC domain containing 2	0.352	0.031	0.167	***0.799***	0.255	***0.056***
Olr715	olfactory receptor 715	0.352	0.028	0.297	***0.948***	0.293	0.040
Duox2	dual oxidase 2	0.352	0.037	0.148	***0.701***	-0.041	***0.363***
Luzp1	leucine zipper protein 1	0.351	0.049	0.041	***0.672***	0.064	***0.768***
Vgll1	vestigial like 1 (Drosophila)	0.350	0.045	0.211	***0.963***	0.281	***0.059***
Sry	sex determining region Y	0.350	0.030	0.079	***0.337***	-0.211	***0.854***
Foxo3	forkhead box O3	0.350	0.026	0.104	***0.664***	0.145	***0.764***
LOC684444	similar to HIStone family member (his-41)	0.350	0.035	0.356	0.021	0.232	***0.876***
Tmem45b	transmembrane protein 45b	0.350	0.034	-0.104	***0.833***	-0.185	***0.905***
Tp53bp2	tumor protein p53 binding protein, 2	0.350	0.043	-0.031	***0.682***	0.081	***0.734***
Slc16a5	solute carrier family 16, member 5 (monocarboxylic acid transporter 6)	0.350	0.046	0.467	0.011	0.288	***0.984***
Man2a1	mannosidase, alpha, class 2A, member 1	0.350	0.047	-0.007	***0.676***	-0.014	***0.633***

Abtb2	ankyrin repeat and BTB (POZ) domain containing 2	0.349	0.045	0.094	***0.757***	0.271	***0.972***
Fam115c	family with sequence similarity 115, member C	0.349	0.027	0.175	***0.840***	0.272	0.037
Kdm2a	lysine (K)-specific demethylase 2A	0.349	0.044	0.233	***0.898***	0.285	***0.057***
Arhgap24	Rho GTPase activating protein 24	0.349	0.038	0.374	0.023	-0.223	***0.942***
Sdr42e1	short chain dehydrogenase/reductase family 42E, member 1	0.349	0.040	-0.068	***0.651***	-0.127	***0.718***
LOC689963	hypothetical protein LOC689963	0.348	0.043	-0.084	***0.756***	0.042	***0.681***
Napepld	N-acyl phosphatidylethanolamine phospholipase D	0.348	0.032	-0.155	***0.654***	-0.139	***0.549***
Vsig8	V-set and immunoglobulin domain containing 8	0.348	0.018	0.092	***0.477***	0.054	***0.427***
Baiap2l1	BAI1-associated protein 2-like 1	0.348	0.042	-0.007	***0.532***	-0.024	***0.576***
Pnp	purine nucleoside phosphorylase	0.348	0.043	0.131	***0.772***	0.022	***0.515***
Lbp	lipopolysaccharide binding protein	0.348	0.004	0.398	0.006	0.202	0.015
LOC680448	similar to protocadherin gamma subfamily B, 5	0.348	0.043	0.247	***0.888***	0.015	***0.548***
Prl8a9	prolactin family 8, subfamily a, member 9	0.348	0.029	-0.026	***0.282***	0.155	***0.067***
Fam120a	family with sequence similarity 120A	0.347	0.044	0.061	***0.360***	0.098	***0.824***
Tmem184b	transmembrane protein 184B	0.347	0.042	0.005	***0.517***	-0.082	***0.732***
Baz2a	bromodomain adjacent to zinc finger domain, 2A	0.347	0.036	0.286	***0.861***	0.252	***0.820***
Entpd2	ectonucleoside triphosphate diphosphohydrolase 2	0.347	0.046	0.125	***0.820***	0.017	***0.632***
Eml2	echinoderm microtubule associated protein like 2	0.347	0.011	0.157	0.033	0.198	0.026
Man1a2	mannosidase, alpha, class 1A, member 2	0.347	0.049	-0.019	***0.640***	0.138	***0.882***
S100a13	S100 calcium binding protein A13	0.347	0.037	0.076	***0.539***	0.102	***0.655***
LOC683313	similar to keratin complex 2, basic, gene 6a	0.347	0.030	0.222	***0.912***	0.202	***0.830***
Slc35a3	solute carrier family 35 (UDP-N-acetylglucosamine (UDP-GlcNAc) transporter), member A3	0.347	0.041	-0.557	0.001	-0.413	0.006
Rdx	radixin	0.346	0.042	0.185	***0.830***	0.081	***0.799***
A1i3	alpha-1-inhibitor III	0.346	0.030	0.030	***0.489***	-0.155	***0.669***
Lmo1	LIM domain only 1	0.346	0.041	-0.046	***0.685***	-0.251	***0.962***
Serpinb6b	serine (or cysteine) peptidase inhibitor, clade B, member 6b	0.346	0.020	0.466	0.024	0.692	0.006
LOC498972	similar to copine II	0.346	0.027	0.150	***0.869***	-0.061	***0.610***
Usp51	ubiquitin specific peptidase 51	0.345	0.032	0.021	***0.248***	0.059	***0.571***
Gss	glutathione synthetase	0.345	0.049	0.085	***0.788***	0.023	***0.737***
RGD1308019	similar to hypothetical protein FLJ20245	0.344	0.014	0.128	***0.544***	0.047	***0.450***
Ugt1a6	UDP glucuronosyltransferase 1 family, polypeptide A6	0.344	0.044	0.019	***0.514***	-0.016	***0.509***
Ctnnd1	catenin (cadherin associated protein), delta 1	0.344	0.016	0.020	***0.303***	0.344	0.011
Rhobtb3	Rho-related BTB domain containing 3	0.344	0.033	0.102	***0.623***	0.076	***0.703***
Cenpq	centromere protein Q	0.343	0.038	-0.012	***0.514***	-0.114	***0.729***
Med28	mediator complex subunit 28	0.343	0.043	0.021	***0.565***	-0.007	***0.429***
Olr155	olfactory receptor 155	0.343	0.049	0.165	***0.939***	0.253	***0.987***
Clec2dl1	C-type lectin domain family 2 member D-like 1	0.342	0.020	0.110	***0.666***	0.162	***0.770***
Disp1	dispatched homolog 1 (Drosophila)	0.342	0.048	0.231	***0.910***	0.015	***0.607***
Crebbp	CREB binding protein	0.342	0.045	0.088	***0.664***	0.238	***0.917***
Kif18b	kinesin family member 18B	0.342	0.041	0.097	***0.475***	-0.020	***0.522***
Msc	musculin	0.341	0.046	0.416	0.015	0.200	***0.868***
LOC684745	similar to germinal histone H4 gene	0.341	0.045	-0.101	***0.800***	0.034	***0.704***
Cxxc4	CXXC finger protein 4	0.341	0.032	-0.124	***0.545***	-0.210	***0.825***
LOC679800	similar to MIC2 like 1	0.341	0.042	0.131	***0.750***	0.127	***0.780***
Gpr19	G protein-coupled receptor 19	0.341	0.047	0.240	***0.944***	0.001	***0.649***
RGD1308907	similar to FLJ20689	0.341	0.049	0.055	***0.632***	0.194	***0.927***
LOC683410	similar to ankyrin repeat domain 33	0.340	0.034	0.015	***0.383***	0.121	***0.550***
Dgkd	diacylglycerol kinase, delta	0.339	0.026	0.070	***0.515***	0.271	0.027
Herpud1	homocysteine-inducible, endoplasmic reticulum stress-inducible, ubiquitin-like domain member 1	0.339	0.035	0.407	0.015	0.519	0.002
Sh3rf2	SH3 domain containing ring finger 2	0.339	0.048	0.050	***0.787***	-0.084	***0.790***
Dgcr8	DiGeorge syndrome critical region gene 8	0.338	0.046	0.283	***0.932***	0.064	***0.543***
Upp2	uridine phosphorylase 2	0.338	0.047	0.093	***0.723***	0.237	***0.849***
Osbp2	oxysterol binding protein 2	0.338	0.045	0.045	***0.553***	-0.087	***0.757***
Car15	carbonic anhydrase 15	0.338	0.046	0.137	***0.846***	-0.064	***0.680***
Prkab2	protein kinase, AMP-activated, beta 2 non-catalytic subunit	0.338	0.039	0.722	0.000	0.344	0.019
Atp10b	ATPase, class V, type 10B	0.337	0.037	0.051	***0.584***	-0.054	***0.589***
Tmem132c	transmembrane protein 132C	0.337	0.047	0.391	0.015	-0.128	***0.837***
Trim31	tripartite motif-containing 31	0.337	0.046	-0.038	***0.650***	0.037	***0.352***
Foxd2	forkhead box D2	0.337	0.043	0.041	***0.692***	0.137	***0.854***

Gpa33	glycoprotein A33 (transmembrane)	0.337	0.047	0.091	***0.766***	0.074	***0.667***
Ptprk	protein tyrosine phosphatase, receptor type, K, extracellular region	0.337	0.046	0.142	***0.849***	0.138	***0.870***
Serinc5	serine incorporator 5	0.337	0.048	0.041	***0.705***	0.018	***0.738***
Wdr11	WD repeat domain 11	0.336	0.047	0.066	***0.735***	0.072	***0.727***
Slc10a5	solute carrier family 10 (sodium/bile acid cotransporter family), member 5	0.336	0.029	-0.055	***0.583***	0.265	0.044
Timp4	tissue inhibitor of metalloproteinase 4	0.336	0.040	0.062	***0.642***	-0.185	***0.853***
Gpr21	G protein-coupled receptor 21	0.336	0.042	0.383	0.021	0.557	0.003
Xpr1	xenotropic and polytropic retrovirus receptor 1	0.335	0.016	-0.103	***0.515***	-0.005	***0.467***
Olr1057	olfactory receptor 1057	0.335	0.047	0.479	0.007	0.359	0.018
Setx	senataxin	0.335	0.045	0.204	***0.884***	0.348	0.034
Hsd17b2	hydroxysteroid (17-beta) dehydrogenase 2	0.335	0.041	0.023	***0.427***	0.081	***0.157***
Clca2	chloride channel calcium activated 2	0.334	0.041	0.303	***0.971***	0.441	0.006
RGD1565975	RGD1565975	0.334	0.030	0.195	***0.800***	0.044	***0.345***
Hist2h3c	histone cluster 2, H3c	0.333	0.023	-0.106	***0.593***	0.090	***0.503***
Rnf34	ring finger protein 34	0.333	0.030	0.085	***0.730***	0.172	***0.890***
LOC361914	similar to solute carrier family 7 (cationic amino acid transporter, y+ system), member 12	0.333	0.046	0.494	0.007	0.575	0.001
Ephb6	Eph receptor B6	0.333	0.033	-0.050	***0.497***	-0.129	***0.641***
Tnks	tankyrase, TRF1-interacting ankyrin-related ADP-ribose polymerase	0.333	0.050	0.230	***0.942***	0.257	***0.924***
Cep57l1	centrosomal protein 57-like 1	0.332	0.034	0.042	***0.577***	0.192	***0.912***
Pdzd8	PDZ domain containing 8	0.332	0.031	-0.074	***0.419***	0.094	***0.465***
LOC499331	similar to hypothetical protein D030056L22	0.332	0.040	0.142	***0.794***	-0.143	***0.624***
Ctdspl	CTD (carboxy-terminal domain, RNA polymerase II, polypeptide A) small phosphatase-like	0.332	0.047	-0.051	***0.707***	-0.051	***0.747***
Stap1	signal transducing adaptor family member 1	0.332	0.048	0.117	***0.125***	0.295	0.033
RGD1561819	similar to Natural killer cell protease 1 precursor (RNKP-1) (Granzyme B)	0.332	0.021	0.842	0.000	0.496	0.002
Ptpre	protein tyrosine phosphatase, receptor type, E	0.331	0.047	0.305	***0.809***	0.359	0.029
Cblb	Cas-Br-M (murine) ecotropic retroviral transforming sequence b	0.331	0.041	0.312	***0.847***	0.367	0.032
Fmr1	fragile X mental retardation 1	0.331	0.033	0.313	***0.845***	0.111	***0.699***
Clec14a	C-type lectin domain family 14, member A	0.330	0.025	0.210	***0.637***	0.042	***0.498***
Slco2a1	solute carrier organic anion transporter family, member 2a1	0.329	0.049	0.085	***0.693***	0.049	***0.540***
Accn2	amiloride-sensitive cation channel 2, neuronal	0.328	0.019	0.193	***0.810***	0.109	***0.775***
Marveld2	MARVEL domain containing 2	0.328	0.043	0.013	***0.551***	-0.026	***0.526***
Pik3ca	phosphoinositide-3-kinase, catalytic, alpha polypeptide	0.328	0.043	0.238	***0.907***	0.247	***0.921***
Prg2	proteoglycan 2, bone marrow	0.328	0.000	0.484	0.001	0.276	0.003
Add2	adducin 2 (beta)	0.328	0.037	0.384	0.019	0.255	***0.058***
Prorsd1	prolyl-tRNA synthetase associated domain containing 1	0.327	0.029	0.145	***0.733***	0.320	0.016
Adamts12	ADAM metallopeptidase with thrombospondin type 1 motif, 12	0.327	0.049	0.251	***0.903***	0.051	***0.595***
Nr1d2	nuclear receptor subfamily 1, group D, member 2	0.327	0.031	0.188	***0.783***	0.443	0.004
Pld3	phospholipase D family, member 3	0.327	0.044	0.116	***0.847***	0.096	***0.778***
LOC100125361	zinc finger protein LOC100125361	0.327	0.049	-0.007	***0.513***	0.191	***0.891***
Klb-ps1	klotho beta, pseudogene 1	0.327	0.045	0.251	***0.496***	0.118	***0.753***
Kdm6a	lysine (K)-specific demethylase 6A	0.326	0.040	0.087	***0.707***	-0.019	***0.472***
Cd300lg	Cd300 molecule-like family member G	0.326	0.040	0.395	0.020	-0.113	***0.397***
LOC689919	similar to 40S ribosomal protein S26	0.326	0.045	-0.027	***0.518***	-0.059	***0.604***
Fam118b	family with sequence similarity 118, member B	0.325	0.039	0.150	***0.774***	0.010	***0.513***
Gna14	guanine nucleotide binding protein, alpha 14	0.325	0.037	0.087	***0.696***	0.180	***0.819***
Rars	arginyl-tRNA synthetase	0.325	0.045	0.150	***0.821***	0.081	***0.608***
Nlrp9	NLR family, pyrin domain containing 9	0.325	0.036	0.407	0.009	0.332	0.022
Tln2	talin 2	0.324	0.046	0.073	***0.477***	0.030	***0.628***
Arl5c	ADP-ribosylation factor-like 5C	0.324	0.020	0.580	0.005	0.767	0.003
Efcab4b	EF-hand calcium binding domain 4B	0.324	0.046	0.136	***0.792***	0.226	***0.900***
Fcgr2b	Fc fragment of IgG, low affinity IIb, receptor (CD32)	0.324	0.033	0.293	0.042	0.582	0.015
Cdc14a	CDC14 cell division cycle 14 homolog A (S. cerevisiae)	0.323	0.019	-0.125	***0.495***	-0.014	***0.410***
Ralbp1	ralA binding protein 1	0.323	0.043	0.019	***0.483***	0.082	***0.721***
Kcnd2	potassium voltage-gated channel, Shal-related subfamily, member 2	0.323	0.033	0.229	***0.886***	0.230	***0.717***

Gcom1	GRINL1A complex locus 1	0.322	0.048	-0.034	***0.470***	-0.070	***0.706***
Fbxo32	F-box protein 32	0.322	0.034	0.404	0.008	0.683	0.000
Mtmr4	myotubularin related protein 4	0.322	0.048	0.293	***0.987***	0.403	0.010
Prtfdc1	phosphoribosyl transferase domain containing 1	0.322	0.048	0.079	***0.568***	0.011	***0.627***
Arhgap15	Rho GTPase activating protein 15	0.322	0.032	0.475	0.014	0.695	0.002
RGD1561149	similar to mKIAA1522 protein	0.322	0.028	-0.089	***0.481***	-0.166	***0.641***
Hes1	hairy and enhancer of split 1 (Drosophila)	0.321	0.020	-0.064	0.049	-0.046	***0.201***
Prrg4	proline rich Gla (G-carboxyglutamic acid) 4 (transmembrane)	0.321	0.050	0.059	***0.699***	0.019	***0.670***
Ifnar1	interferon (alpha, beta and omega) receptor 1	0.321	0.042	0.270	***0.947***	0.349	0.025
Fcrlb	Fc receptor-like B	0.321	0.029	0.353	0.023	-0.008	***0.430***
Atf6	activating transcription factor 6	0.320	0.015	-0.111	***0.316***	0.006	***0.370***
LOC691895	similar to ferritin, heavy polypeptide-like 17	0.320	0.045	0.283	***0.973***	0.295	0.044
LOC685994	similar to Spetex-2C protein	0.320	0.031	0.378	0.012	0.336	0.018
Shpk	sedoheptulokinase	0.320	0.040	-0.020	***0.507***	-0.169	***0.804***
Nr1h4	nuclear receptor subfamily 1, group H, member 4	0.319	0.039	0.163	***0.781***	0.166	***0.683***
Akna	AT-hook transcription factor	0.318	0.048	0.695	0.009	0.869	0.003
Agpat5	1-acylglycerol-3-phosphate O-acyltransferase 5 (lysophosphatidic acid acyltransferase, epsilon)	0.318	0.032	0.154	***0.764***	0.478	0.003
LOC690463	hypothetical protein LOC690463	0.317	0.024	0.119	***0.560***	0.232	0.030
Mboat2	membrane bound O-acyltransferase domain containing 2	0.317	0.040	-0.022	***0.462***	0.043	***0.561***
Irf1	interferon regulatory factor 1	0.317	0.049	-0.082	***0.666***	-0.063	***0.692***
Krt80	keratin 80	0.317	0.044	0.059	***0.647***	0.246	***0.923***
Sesn3	sestrin 3	0.316	0.047	0.263	***0.586***	0.255	***0.874***
Stk4	serine/threonine kinase 4	0.316	0.047	0.166	***0.405***	0.365	0.028
Hist2h4	histone cluster 2, H4	0.316	0.036	-0.121	***0.682***	0.115	***0.725***
Abcc1	ATP-binding cassette, subfamily C (CFTR/MRP), member 1	0.316	0.019	0.343	0.020	0.356	0.014
Whsc1	Wolf-Hirschhorn syndrome candidate 1 (human)	0.316	0.045	0.159	***0.694***	0.095	***0.613***
Hnf1a	HNF1 homeobox A	0.316	0.030	-0.037	***0.383***	-0.132	***0.561***
Ptprc	protein tyrosine phosphatase, receptor type, C	0.316	0.023	0.759	0.003	1.034	0.000
LOC497934	similar to hypothetical protein FLJ20014	0.315	0.047	-0.002	***0.449***	-0.046	***0.622***
Kdm1b	lysine (K)-specific demethylase 1B	0.315	0.034	0.161	***0.554***	0.249	***0.054***
Hist2h4	histone cluster 2, H4	0.313	0.028	-0.031	***0.349***	0.008	***0.355***
RT1-T24-1	RT1 class I, locus T24, gene 1	0.313	0.045	0.118	***0.470***	0.143	***0.818***
Upf2	UPF2 regulator of nonsense transcripts homolog (yeast)	0.313	0.035	0.214	***0.836***	0.326	0.030
Osbpl3	oxysterol binding protein-like 3	0.313	0.041	-0.339	0.012	-0.091	***0.480***
Bard1	BRCA1 associated RING domain 1	0.312	0.047	0.152	***0.760***	-0.153	***0.759***
RGD1309049	similar to RIKEN cDNA 4933415F23	0.312	0.043	-0.060	***0.415***	-0.223	***0.836***
Iqcf2	IQ motif containing F2	0.312	0.039	0.119	***0.272***	0.122	***0.714***
S100a1	S100 calcium binding protein A1	0.312	0.026	0.400	0.012	0.203	***0.770***
Pola1	polymerase (DNA directed), alpha 1	0.311	0.044	0.210	***0.921***	0.000	***0.471***
Epas1	endothelial PAS domain protein 1	0.309	0.004	-0.122	***0.115***	-0.111	***0.118***
Abcd3	ATP-binding cassette, subfamily D (ALD), member 3	0.307	0.039	0.044	***0.409***	0.112	***0.489***
Cpa5	carboxypeptidase A5	0.306	0.037	0.195	***0.845***	0.326	0.026
RGD1562339	RGD1562339	0.305	0.026	0.064	***0.621***	0.120	***0.659***
Jsrp1	junctional sarcoplasmic reticulum protein 1	0.305	0.011	0.662	0.005	0.984	0.001
Psrc1	proline/serine-rich coiled-coil 1	0.304	0.048	-0.034	***0.646***	-0.055	***0.627***
Shprh	SNF2 histone linker PHD RING helicase	0.303	0.029	0.388	0.008	0.282	0.035
Gpr35	G protein-coupled receptor 35	0.302	0.036	-0.190	***0.752***	-0.344	***0.944***
Eya3	eyes absent homolog 3 (Drosophila)	0.302	0.049	-0.017	***0.397***	0.083	***0.609***
Ugt2a3	UDP glucuronosyltransferase 2 family, polypeptide A3	0.301	0.049	0.375	0.021	0.162	***0.783***
LOC499407	LRRGT00097	0.301	0.047	0.330	0.030	0.305	0.035
Pla2g2f	phospholipase A2, group IIF	0.300	0.046	-0.016	***0.232***	-0.003	***0.225***
Cnr2	cannabinoid receptor 2 (macrophage)	0.299	0.007	0.372	0.008	0.588	0.005
Fmr1	fragile X mental retardation 1	0.299	0.038	0.155	***0.545***	0.183	***0.686***
Hk2	hexokinase 2	0.299	0.048	0.318	0.028	0.644	0.001
Spsb4	splA/ryanodine receptor domain and SOCS box containing 4	0.299	0.044	-0.233	***0.706***	-0.299	***0.925***
Ripk1	receptor (TNFRSF)-interacting serine-threonine kinase 1	0.298	0.035	-0.010	***0.497***	0.043	***0.541***
RGD1311558	similar to 4930506M07Rik protein	0.298	0.035	0.060	***0.416***	-0.093	***0.340***
Alkbh4	alkB, alkylation repair homolog 4 (E. coli)	0.297	0.044	-0.111	***0.611***	-0.289	***0.902***

LOC688708	similar to ATP-binding cassette, sub-family C (CFTR/MRP), member 1	0.296	0.028	0.292	0.033	0.329	0.020
RGD1561065	similar to mKIAA1111 protein	0.296	0.035	0.176	***0.369***	0.306	0.018
Taf15	TAF15 RNA polymerase II, TATA box binding protein (TBP)-associated factor	0.296	0.031	0.126	***0.668***	0.177	***0.711***
Hmg1l1	high-mobility group (nonhistone chromosomal) protein 1-like 1	0.295	0.047	0.188	***0.396***	0.041	***0.579***
Slc6a8	solute carrier family 6 (neurotransmitter transporter, creatine), member 8	0.294	0.037	0.045	***0.490***	-0.051	***0.380***
LOC688276	similar to epidermodysplasia verruciformis 2	0.294	0.016	0.694	0.003	0.835	0.001
March3	membrane-associated ring finger (C3HC4) 3	0.294	0.017	0.144	0.048	0.302	0.013
Sorl1	sortilin-related receptor, LDLR class A repeats-containing	0.293	0.017	0.397	0.017	0.372	0.019
Irf2bpl	interferon regulatory factor 2 binding protein-like	0.293	0.034	0.037	***0.500***	-0.073	***0.466***
Ifi203-ps1	interferon activated gene 203, pseudogene 1	0.293	0.003	0.010	0.005	0.432	0.002
Ganc	glucosidase, alpha; neutral C	0.292	0.033	-0.020	***0.332***	0.048	***0.307***
Plekha5	pleckstrin homology domain containing, family A member 5	0.292	0.049	0.074	***0.592***	0.100	***0.718***
Sema4c	sema domain, immunoglobulin domain (Ig), transmembrane domain (TM) and short cytoplasmic domain, (semaphorin) 4C	0.291	0.036	0.415	0.008	0.374	0.011
LOC100364624	hypothetical protein LOC100364624	0.291	0.029	-0.086	***0.188***	-0.268	0.028
Mical1	microtubule associated monoxygenase, calponin and LIM domain containing 1	0.290	0.038	0.027	***0.506***	-0.019	***0.382***
Ppargc1a	peroxisome proliferator-activated receptor gamma, coactivator 1 alpha	0.289	0.028	-0.032	***0.221***	0.362	0.009
Scara5	scavenger receptor class A, member 5 (putative)	0.289	0.046	0.208	***0.831***	0.110	***0.709***
Cys1	cystin 1	0.289	0.049	0.244	***0.914***	0.085	***0.609***
Magebl1	melanoma antigen, family B-like 1	0.288	0.033	-0.098	***0.419***	-0.134	***0.328***
Ptprcap	protein tyrosine phosphatase, receptor type, C-associated protein	0.287	0.007	0.566	0.005	0.827	0.000
Cd79a	Cd79a molecule, immunoglobulin-associated alpha	0.287	0.017	0.458	0.008	0.613	0.004
Tpo	thyroid peroxidase	0.287	0.033	0.036	***0.403***	-0.332	***0.815***
Tnks2	tankyrase, TRF1-interacting ankyrin-related ADP-ribose polymerase 2	0.286	0.041	0.045	***0.511***	0.074	***0.538***
Cd300lg	Cd300 molecule-like family member G	0.285	0.035	0.135	***0.671***	-0.505	0.001
Grem2	gremlin 2	0.282	0.042	0.563	0.002	0.137	***0.785***
Rasal3	RAS protein activator like 3	0.282	0.042	0.583	0.006	0.743	0.002
Arhgdib	Rho, GDP dissociation inhibitor (GDI) beta	0.282	0.046	0.518	0.007	0.563	0.002
LOC296778	similar to Integrin alpha-6 precursor (VLA-6) (CD49f antigen)	0.281	0.012	-0.484	0.004	-0.732	0.000
Cd5	Cd5 molecule	0.281	0.043	0.415	0.050	0.713	0.009
Gpr18	G protein-coupled receptor 18	0.281	0.005	0.537	0.002	0.759	0.000
LOC689656	hypothetical protein LOC689656	0.281	0.024	0.052	***0.104***	-0.100	***0.227***
Lamc1	laminin, gamma 1	0.280	0.039	0.252	***0.574***	0.161	***0.717***
Etnk1	ethanolamine kinase 1	0.278	0.022	0.025	***0.105***	0.068	***0.069***
Fbxw8	F-box and WD repeat domain containing 8	0.278	0.047	0.018	***0.453***	-0.007	***0.397***
Ssh2	slingshot homolog 2 (Drosophila)	0.278	0.046	0.013	***0.268***	0.083	***0.517***
RGD1559622	similar to hypothetical protein C130079G13	0.277	0.028	0.425	0.005	0.334	0.010
LOC683844	similar to RING finger protein 11 (NEDD4 WW domain-binding protein 2) (Sid 1669)	0.275	0.044	0.000	***0.179***	-0.485	0.003
Kcnh3	potassium voltage-gated channel, subfamily H (eag-related), member 3	0.274	0.035	0.381	0.013	0.159	***0.683***
Jak3	Janus kinase 3	0.274	0.019	0.597	0.004	0.575	0.003
Tbc1d5	TBC1 domain family, member 5	0.274	0.030	-0.020	***0.422***	0.064	***0.480***
Inpp4a	inositol polyphosphate-4-phosphatase, type 1	0.273	0.049	0.199	***0.852***	0.155	***0.799***
Med14	mediator complex subunit 14	0.273	0.031	-0.019	***0.246***	0.093	***0.354***
Ins1	insulin 1	0.273	0.027	0.427	0.004	0.458	0.003
Atp13a3	ATPase type 13A3	0.273	0.030	0.037	***0.153***	-0.027	***0.274***
RGD1310935	similar to Dermal papilla derived protein 7	0.271	0.016	0.332	0.012	0.484	0.003
Tgfbr2	transforming growth factor, beta receptor II	0.271	0.044	0.146	***0.749***	0.174	***0.757***
Pvrl4	poliovirus receptor-related 4	0.270	0.024	0.088	***0.447***	-0.145	***0.418***
Etv4	ets variant 4	0.269	0.038	0.014	***0.353***	0.018	***0.539***
Ghr	growth hormone receptor	0.269	0.035	0.085	***0.183***	0.181	***0.638***
RGD1562552	similar to hypothetical protein LOC340061	0.269	0.026	0.458	0.012	0.485	0.008
Coro1a	coronin, actin binding protein 1A	0.268	0.041	0.632	0.003	0.749	0.000

Wdr91	WD repeat domain 91	0.268	0.039	0.021	***0.339***	0.177	***0.068***
Il22ra2	interleukin 22 receptor, alpha 2	0.268	0.013	0.702	0.000	0.520	0.004
LOC690347	hypothetical protein LOC690347	0.267	0.032	0.145	***0.646***	-0.192	***0.539***
RGD1310427	similar to KIAA0090 protein	0.267	0.039	0.018	***0.063***	-0.014	***0.087***
Col4a5	collagen, type IV, alpha 5	0.266	0.046	0.474	0.009	0.218	***0.881***
Sgsm1	small G protein signaling modulator 1	0.266	0.048	0.680	0.002	0.719	0.000
Arl4c-ps1	ADP-ribosylation factor-like 4C, pseudogene 1	0.263	0.033	0.342	0.016	0.325	0.039
Scara5	scavenger receptor class A, member 5 (putative)	0.261	0.035	0.170	***0.704***	0.001	***0.185***
Pnpo	pyridoxine 5'-phosphate oxidase	0.260	0.038	0.374	0.008	0.330	0.014
Limd2	LIM domain containing 2	0.258	0.011	0.608	0.002	0.456	0.001
Zap70	zeta-chain (TCR) associated protein kinase	0.258	0.048	0.599	0.011	0.718	0.006
Prkd3	protein kinase D3	0.256	0.030	0.041	***0.371***	0.221	0.028
Dusp15	dual specificity phosphatase 15	0.255	0.043	0.364	0.017	0.102	***0.486***
Il7r	interleukin 7 receptor	0.254	0.028	0.206	0.017	0.484	0.007
RGD1564400	similar to Eukaryotic translation initiation factor 5 (eIF-5)	0.253	0.045	0.502	0.002	-0.016	***0.417***
Evc2	Ellis van Creveld syndrome 2 homolog (human)	0.252	0.042	-0.145	***0.487***	-0.069	***0.273***
LOC681167	hypothetical protein LOC681167	0.250	0.037	0.138	***0.596***	0.279	0.021
Pam	peptidylglycine alpha-amidating monooxygenase	0.249	0.042	0.200	***0.698***	0.244	0.035
LOC691259	hypothetical protein LOC691259	0.249	0.039	0.097	***0.302***	0.084	***0.090***
Pigz	phosphatidylinositol glycan anchor biosynthesis, class Z	0.248	0.041	-0.099	***0.084***	-0.169	***0.059***
RGD1565591	similar to Ski protein	0.247	0.025	0.100	***0.598***	0.092	***0.520***
Lrmp	lymphoid-restricted membrane protein	0.246	0.035	0.506	0.005	0.425	0.017
Far2	fatty acyl CoA reductase 2	0.246	0.033	0.755	0.000	0.722	0.000
LOC500700	similar to chromosome 14 open reading frame 145	0.246	0.046	0.175	***0.609***	-0.002	***0.234***
LOC298138	similar to RAS and EF hand domain containing	0.246	0.033	0.385	0.008	0.147	***0.053***
Cd8a	CD8a molecule	0.245	0.034	0.348	0.030	0.499	0.016
Tnf	tumor necrosis factor	0.245	0.035	0.385	0.010	0.530	0.003
Hist1h4b	histone cluster 1, H4b	0.245	0.048	-0.143	***0.634***	0.109	***0.656***
Sep-01	septin 1	0.244	0.038	0.434	0.012	0.694	0.003
Adamts9	a disintegrin-like and metalloprotease (reprolysin type) with thrombospondin type 1 motif, 9	0.244	0.046	0.398	0.008	0.118	***0.064***
Gpr18	G protein-coupled receptor 18	0.243	0.010	0.457	0.005	0.611	0.002
Slamf6	SLAM family member 6	0.242	0.015	0.028	0.045	0.563	0.002
Hist2h3c2	histone cluster 2, H3c2	0.242	0.047	0.016	***0.475***	0.000	***0.427***
Cd19	CD19 molecule	0.242	0.001	0.726	0.000	0.852	0.000
Ccng2	cyclin G2	0.241	0.021	-0.042	***0.371***	0.100	***0.054***
Tcrb	T-cell receptor beta chain	0.239	0.038	0.316	0.025	0.309	0.029
Ahnak	AHNAK nucleoprotein	0.239	0.028	0.012	***0.348***	-0.022	***0.316***
Btla	B and T lymphocyte associated	0.237	0.009	0.469	0.010	0.751	0.002
Prap1	proline-rich acidic protein 1	0.237	0.049	0.032	***0.064***	0.007	***0.054***
Ankle2	ankyrin repeat and LEM domain containing 2	0.235	0.048	0.181	***0.745***	0.335	0.012
Rep15	RAB15 effector protein	0.233	0.040	-0.198	***0.430***	-0.064	***0.226***
Sin3a	SIN3 homolog A, transcription regulator (yeast)	0.233	0.028	0.035	***0.085***	0.038	***0.080***
Lrrc48	leucine rich repeat containing 48	0.232	0.032	-0.232	***0.499***	-0.195	0.029
Ccdc169	coiled-coil domain containing 169	0.231	0.045	-0.018	***0.270***	-0.167	***0.554***
Btla	B and T lymphocyte associated	0.229	0.026	0.201	0.039	0.499	0.012
Col6a3	procollagen, type VI, alpha 3	0.229	0.040	0.191	***0.592***	0.103	***0.088***
Znrf4	zinc and ring finger 4	0.226	0.037	-0.223	***0.493***	0.019	***0.377***
Cdh23	cadherin-related 23	0.225	0.048	0.116	***0.511***	-0.206	***0.540***
Olr1570	olfactory receptor 1570	0.224	0.021	-0.267	0.037	-0.680	0.002
Gpr68	G protein-coupled receptor 68	0.223	0.018	0.413	0.016	0.679	0.006
Olr415	olfactory receptor 415	0.220	0.040	-0.164	***0.473***	-0.354	***0.839***
Stmn2	stathmin-like 2	0.220	0.046	0.136	***0.688***	0.150	***0.665***
Il16	interleukin 16	0.219	0.021	0.448	0.009	0.523	0.001
Olr390	olfactory receptor 390	0.218	0.036	0.389	0.015	0.550	0.004
Fam168a	family with sequence similarity 168, member A	0.218	0.038	0.239	0.028	0.402	0.005
Cd37	CD37 molecule	0.218	0.040	0.366	0.039	0.542	0.014
RGD1306739	similar to RIKEN cDNA 1700040L02	0.217	0.046	0.063	***0.285***	0.230	***0.157***
LOC100364854	similar to RIKEN cDNA A430107P09 gene-like	0.217	0.031	0.326	***0.075***	0.535	0.018
Slfn5	schlafen family member 5	0.215	0.009	-0.088	***0.271***	-0.022	***0.086***
Abcg1	ATP-binding cassette, subfamily G (WHITE), member 1	0.211	0.032	0.372	0.010	0.107	***0.108***
Cxcr4	chemokine (C-X-C motif) receptor 4	0.210	0.027	0.491	0.014	0.464	0.004
Cyp4f5	cytochrome P450, family 4, subfamily f, polypeptide 5	0.210	0.034	0.187	0.034	-0.107	***0.362***

Rcan3	RCAN family member 3	0.208	0.021	-0.424	***0.622***	-0.171	***0.202***
Chi3l1	chitinase 3-like 1	0.208	0.044	0.841	0.002	0.662	0.002
Esyt3	extended synaptotagmin-like protein 3	0.208	0.026	-0.129	***0.257***	-0.212	***0.415***
Cybasc3	cytochrome b, ascorbate dependent 3	0.206	0.017	0.163	***0.065***	0.300	0.027
Mfng	MFNG O-fucosylpeptide 3-beta-N-acetylglucosaminyltransferase	0.204	0.023	0.220	0.041	0.249	0.028
Spn	sialophorin	0.204	0.023	0.203	***0.094***	0.660	0.008
Ing3	inhibitor of growth family, member 3	0.202	0.042	0.336	0.010	0.338	0.008
Slc39a2	solute carrier family 39 (zinc transporter), member 2	0.202	0.041	0.252	0.027	-0.022	***0.194***
Itk	IL2-inducible T-cell kinase	0.201	0.036	0.345	0.015	0.656	0.004
Epha1	Eph receptor A1	0.199	0.046	-0.060	***0.194***	-0.022	***0.158***
Megf8	multiple EGF-like-domains 8	0.198	0.050	-0.231	***0.536***	-0.533	0.007
Acsf3	acyl-CoA synthetase family member 3	0.196	0.012	0.170	0.012	-0.032	0.030
Tulp1	tubby like protein 1	0.195	0.041	-0.069	***0.199***	0.107	***0.057***
Gpr174	G protein-coupled receptor 174	0.193	0.021	0.159	0.029	0.337	0.010
Abcg2	ATP-binding cassette, subfamily G (WHITE), member 2	0.192	0.014	0.141	0.013	0.163	0.013
Olr1337	olfactory receptor 1337	0.190	0.040	-0.183	***0.330***	-0.337	***0.627***
Sell	selectin L	0.186	0.010	-0.052	0.020	0.358	0.006
Pou2af1	POU class 2 associating factor 1	0.184	0.023	0.076	***0.065***	0.455	0.005
LOC691952	hypothetical protein LOC691952	0.181	0.005	-0.279	***0.082***	-0.760	0.008
Zc3h11a	zinc finger CCCH-type containing 11A	0.180	0.030	0.334	0.015	0.426	0.005
Gast	gastrin	0.180	0.047	0.341	0.027	0.177	0.046
Skil	SKI-like oncogene	0.177	0.043	-0.051	***0.312***	0.079	***0.084***
Acsm3	acyl-CoA synthetase medium-chain family member 3	0.168	0.008	-0.179	0.045	0.758	0.000
Lphn2	latrophilin 2	0.164	0.031	0.170	0.026	-0.149	***0.169***
Faim3	Fas apoptotic inhibitory molecule 3	0.163	0.012	0.581	0.005	0.563	0.005
Ncor1	nuclear receptor co-repressor 1	0.162	0.013	-0.041	***0.068***	0.197	0.007
Tbc1d10c	TBC1 domain family, member 10C	0.162	0.022	0.585	0.003	0.550	0.002
Vwc2l	von Willebrand factor C domain-containing protein 2-like	0.161	0.022	-0.177	***0.238***	-0.264	***0.346***
Cd3e	CD3 molecule, epsilon	0.160	0.022	0.062	***0.093***	0.394	0.012
Zfp608	zinc finger protein 608	0.160	0.033	-0.177	***0.239***	-0.229	***0.277***
Retn	resistin	0.159	0.014	0.765	0.000	-0.125	0.020
Sertad3	SERTA domain containing 3	0.158	0.039	-0.244	***0.056***	-0.383	***0.675***
LOC680810	hypothetical protein LOC680810	0.157	0.033	-0.202	***0.064***	-0.296	0.030
Bank1	B-cell scaffold protein with ankyrin repeats 1	0.153	0.001	0.127	0.002	0.549	0.001
Fcrla	Fc receptor-like A	0.149	0.011	0.821	0.003	0.711	0.002
Myo1g	myosin IG	0.147	0.046	0.501	0.006	0.564	0.007
Pou2af1	POU class 2 associating factor 1	0.147	0.026	0.212	0.013	0.575	0.001
Map4k1	mitogen activated protein kinase kinase kinase kinase 1	0.145	0.036	0.430	0.029	0.585	0.011
Pabpc2	poly(A) binding protein, cytoplasmic 2	0.142	0.030	-0.206	***0.205***	-0.500	0.024
RGD1561381	similar to microsomal glutathione S-transferase 3	0.140	0.040	-0.016	***0.052***	-0.117	0.030
Adh6a	alcohol dehydrogenase 6A (class V)	0.139	0.021	0.197	0.041	0.141	***0.683***
RT1-DOb	RT1 class II, locus DOb	0.134	0.036	0.555	0.007	0.763	0.001
Actn3	actinin alpha 3	0.130	0.027	0.191	0.024	0.251	***0.058***
Fcrla	Fc receptor-like A	0.128	0.001	0.881	0.001	0.467	0.001
Sbk1	SH3-binding domain kinase 1	0.128	0.023	0.033	0.040	0.165	0.035
Siglec10	sialic acid binding Ig-like lectin 10	0.126	0.003	0.844	0.001	0.818	0.001
Mfge8	milk fat globule-EGF factor 8 protein	0.124	0.015	0.270	0.016	0.162	0.014
Madcam1	mucosal vascular addressin cell adhesion molecule 1	0.118	0.027	0.359	0.017	0.454	0.023
Fam46b	family with sequence similarity 46, member B	0.116	0.030	0.122	0.028	-0.248	***0.336***
Usp6nl	USP6 N-terminal like	0.113	0.046	-0.220	***0.355***	-0.044	***0.132***
Lcp1	lymphocyte cytosolic protein 1	0.105	0.039	0.327	0.035	0.584	0.006
Ppm1l	protein phosphatase, Mg2+/Mn2+ dependent, 1L	0.094	0.037	0.175	0.011	0.173	0.011
Sipa1	signal-induced proliferation-associated 1	0.093	0.037	0.351	0.011	0.397	0.012
Ahnak	AHNAK nucleoprotein	0.090	0.005	-0.097	0.026	-0.014	0.013
Itsn2	intersectin 2	0.078	0.023	0.514	0.002	0.483	0.003
Il13ra1	interleukin 13 receptor, alpha 1	0.075	0.046	-0.191	***0.313***	0.116	0.017
Clca4	chloride channel accessory 4	0.067	0.015	0.127	0.033	0.867	0.000
S100g	S100 calcium binding protein G	0.066	0.024	1.050	0.000	-0.213	0.006
Arpp21	cAMP-regulated phosphoprotein 21	0.063	0.025	0.383	***0.058***	0.719	0.005
Rnf126	ring finger protein 126	0.055	0.037	-0.178	0.032	-0.162	0.033

LOC691047	similar to RIKEN cDNA 1700001F22	0.055	0.046	-0.509	0.043	-0.705	0.019
Otud7b	OTU domain containing 7B	0.051	0.039	0.286	0.007	0.489	0.001
Snx20	sorting nexin 20	0.045	0.029	0.528	0.004	0.516	0.001
V1rf4	vomeronasal 1 receptor F4	0.045	0.023	-0.320	***0.091***	-0.587	0.029
Ptprc	protein tyrosine phosphatase, receptor type, C	0.035	0.040	0.648	0.003	0.768	0.000
Lima1	LIM domain and actin binding 1	0.018	0.036	-0.128	***0.078***	0.192	0.003
Ncf1	neutrophil cytosolic factor 1	0.009	0.028	0.448	0.022	0.549	0.010
Ccr6	chemokine (C-C motif) receptor 6	-0.004	0.010	0.087	0.006	0.213	0.003
B4galnt1	beta-1,4-N-acetyl-galactosaminyl transferase 1	-0.014	0.016	0.333	0.027	0.580	0.007
Clvs1	clavesin 1	-0.017	0.040	-0.654	0.005	-0.446	***0.736***
Lcn2	lipocalin 2	-0.028	0.029	0.004	0.007	-0.387	0.001
Wfdc16	WAP four-disulfide core domain 16	-0.036	0.024	-0.399	0.037	-0.923	0.000
LOC499544	LRRGT00154	-0.039	0.024	-0.419	***0.257***	-0.682	0.008
Gal3st2	galactose-3-O-sulfotransferase 2	-0.042	0.035	0.032	0.030	0.114	0.018
Hmha1	histocompatibility (minor) HA-1	-0.044	0.018	0.315	0.008	0.482	0.001
Ghrl	ghrelin/obestatin prepropeptide	-0.052	0.015	-0.870	0.000	-0.352	0.001
Reep6	receptor accessory protein 6	-0.052	0.039	0.092	***0.285***	-0.158	***0.844***
Cd6	Cd6 molecule	-0.056	0.003	0.343	0.025	0.549	0.002
LOC363326	hypothetical LOC363326	-0.056	0.023	0.560	0.007	0.548	0.004
Cd69	Cd69 molecule	-0.058	0.008	0.172	***0.073***	0.263	***0.067***
Cd40	CD40 molecule, TNF receptor superfamily member 5	-0.059	0.038	0.381	0.006	0.452	0.003
St3gal6	ST3 beta-galactoside alpha-2,3-sialyltransferase 6	-0.063	0.036	0.229	***0.162***	0.344	***0.584***
Fibin	fin bud initiation factor homolog (zebrafish)	-0.064	0.009	0.116	***0.659***	0.019	***0.340***
LOC100364769	LRRG00136-like	-0.080	0.024	-0.977	0.000	-0.958	0.001
Hspa1b	heat shock 70kD protein 1B (mapped)	-0.081	0.018	0.118	0.003	-0.428	0.034
LOC308990	hypothetical protein LOC308990	-0.087	0.022	0.403	0.020	0.542	0.004
Podnl1	podocan-like 1	-0.101	0.041	0.171	***0.075***	0.327	0.017
Ostalpha	organic solute transporter alpha	-0.110	0.045	-0.598	0.001	-0.903	0.000
Cd40	CD40 molecule, TNF receptor superfamily member 5	-0.112	0.032	0.214	0.025	0.426	0.008
Itga5	integrin, alpha 5 (fibronectin receptor, alpha polypeptide)	-0.118	0.034	-0.033	***0.294***	-0.481	0.007
Cartpt	CART prepropeptide	-0.129	0.045	0.027	***0.298***	-0.131	***0.513***
Fibin	fin bud initiation factor homolog (zebrafish)	-0.132	0.006	0.081	***0.567***	-0.103	***0.511***
LOC305806	similar to glutaredoxin 1 (thioltransferase); glutaredoxin	-0.148	0.035	-0.150	***0.508***	0.043	***0.173***
Ripk3	receptor-interacting serine-threonine kinase 3	-0.154	0.040	-0.053	***0.426***	-0.138	***0.534***
Fcgbp	Fc fragment of IgG binding protein	-0.166	0.039	0.056	***0.405***	0.398	0.016
Ggt1	gamma-glutamyltransferase 1	-0.167	0.011	-0.416	0.010	-0.259	***0.841***
Ptpn22	protein tyrosine phosphatase, non-receptor type 22 (lymphoid)	-0.172	0.019	0.342	***0.076***	0.587	0.009
RGD1563888	similar to DNA segment, Chr 16, ERATO Doi 472, expressed	-0.173	0.044	0.040	***0.352***	0.207	***0.799***
Lyz2	lysozyme 2	-0.177	0.039	0.331	0.004	0.220	0.010
Gstt3	glutathione S-transferase, theta 3	-0.177	0.040	-0.334	0.030	-0.052	***0.318***
Synm	synemin, intermediate filament protein	-0.179	0.023	-0.005	***0.646***	-0.160	***0.799***
Lmod1	leiomodin 1 (smooth muscle)	-0.180	0.047	0.056	***0.713***	-0.113	***0.668***
Steap4	STEAP family member 4	-0.180	0.045	0.104	***0.738***	0.157	***0.824***
Lat2	linker for activation of T cells family, member 2	-0.181	0.045	0.084	***0.244***	0.195	***0.437***
Acap1	ArfGAP with coiled-coil, ankyrin repeat and PH domains 1	-0.182	0.014	0.405	0.006	0.705	0.003
Serpina3n	serine (or cysteine) peptidase inhibitor, clade A, member 3N	-0.185	0.002	-0.092	0.002	-1.808	0.000
Cldn4	claudin 4	-0.185	0.024	0.001	***0.442***	0.476	0.006
Myocd	myocardin	-0.188	0.044	-0.088	***0.502***	-0.076	***0.464***
Ly6c	Ly6-C antigen	-0.189	0.011	0.272	***0.077***	0.484	0.015
Slc7a14	solute carrier family 7 (cationic amino acid transporter, y+ system), member 14	-0.193	0.041	0.230	***0.951***	-0.042	***0.682***
Thoc4	THO complex 4	-0.194	0.050	-0.036	***0.343***	-0.335	0.025
Col3a1	collagen, type III, alpha 1	-0.196	0.039	0.528	0.003	0.218	***0.054***
Ly6g6e	lymphocyte antigen 6 complex, locus G6E	-0.196	0.049	0.107	***0.289***	0.063	***0.152***
Evi2a	ecotropic viral integration site 2A	-0.198	0.022	0.170	***0.091***	0.515	0.007
Serpine1	serpin peptidase inhibitor, clade E (nexin, plasminogen activator inhibitor type 1), member 1	-0.198	0.011	-0.608	0.001	-0.707	0.000
Htr3a	5-hydroxytryptamine (serotonin) receptor 3a	-0.200	0.044	-0.045	***0.751***	-0.063	***0.610***
Cd3g	CD3 molecule, gamma	-0.201	0.003	0.089	0.003	0.272	0.030

Ceacam19	carcinoembryonic antigen-related cell adhesion molecule 19	-0.203	0.034	0.233	***0.098***	0.398	0.036
Gsdmc	gasdermin C	-0.210	0.018	-0.214	***0.415***	-0.119	***0.284***
Pla2g2d	phospholipase A2, group IID	-0.210	0.009	0.430	0.002	0.631	0.001
Htra3	HtrA serine peptidase 3	-0.211	0.030	0.146	***0.753***	-0.010	***0.468***
Atp1a2	ATPase, Na+/K+ transporting, alpha 2 polypeptide	-0.211	0.018	0.426	0.007	0.096	***0.708***
Plek	pleckstrin	-0.214	0.032	0.302	***0.324***	0.563	0.010
Csf1r	colony stimulating factor 1 receptor	-0.214	0.036	0.290	***0.950***	0.146	***0.836***
Phf11	PHD finger protein 11	-0.214	0.018	0.357	0.017	0.619	0.002
Tmsb4x	thymosin beta 4, X-linked	-0.216	0.040	0.010	***0.458***	0.219	***0.750***
Mmp15	matrix metallopeptidase 15	-0.218	0.041	-0.241	***0.971***	-0.129	***0.781***
Spon2	spondin 2, extracellular matrix protein	-0.222	0.040	-0.012	***0.451***	-0.238	***0.924***
Fez1	fasciculation and elongation protein zeta 1 (zygin I)	-0.224	0.046	0.092	***0.544***	-0.159	***0.810***
Igfbp2	insulin-like growth factor binding protein 2	-0.226	0.015	0.254	***0.928***	0.065	***0.602***
Cfh	complement factor H	-0.226	0.027	0.198	***0.902***	0.056	***0.624***
Syp	synaptophysin	-0.227	0.048	0.018	***0.559***	0.009	***0.634***
Efemp1	EGF-containing fibulin-like extracellular matrix protein 1	-0.231	0.046	0.023	***0.485***	-0.221	***0.872***
Ly86	lymphocyte antigen 86	-0.236	0.006	0.287	***0.076***	0.468	0.028
Rbp1	retinol binding protein 1, cellular	-0.237	0.037	-0.043	***0.208***	0.209	0.038
Ccl17	chemokine (C-C motif) ligand 17	-0.239	0.037	-0.019	0.024	0.504	0.040
Rmrp	RNA component of mitochondrial RNA processing endoribonuclease	-0.239	0.045	-0.184	***0.249***	0.089	0.018
Dok3	docking protein 3	-0.241	0.041	0.340	0.018	0.485	0.006
LOC501224	similar to RIKEN cDNA 2610042L04	-0.244	0.042	0.110	***0.680***	0.091	***0.588***
Cebpd	CCAAT/enhancer binding protein (C/EBP), delta	-0.244	0.028	-0.224	***0.690***	-0.709	0.000
Ascl2	achaete-scute complex homolog 2 (Drosophila)	-0.246	0.043	-0.095	***0.626***	-0.315	0.022
Mylk	myosin light chain kinase	-0.246	0.012	-0.058	***0.129***	0.125	0.038
Ly86	lymphocyte antigen 86	-0.247	0.006	0.278	***0.065***	0.454	0.011
Ccl20	chemokine (C-C motif) ligand 20	-0.250	0.036	0.465	0.018	0.526	0.001
Gng13	guanine nucleotide binding protein (G protein), gamma 13	-0.251	0.024	-0.039	***0.238***	-0.111	***0.478***
Fcgbpl1	Fc fragment of IgG binding protein-like 1	-0.255	0.045	0.014	***0.274***	0.290	0.032
Clca1	chloride channel accessory 1	-0.255	0.027	0.012	***0.247***	0.172	***0.060***
Speg	SPEG complex locus	-0.255	0.037	0.182	***0.917***	0.114	***0.589***
Olr991	olfactory receptor 991	-0.256	0.039	0.088	***0.520***	0.134	***0.696***
Myh11	myosin, heavy chain 11, smooth muscle	-0.256	0.033	-0.032	***0.662***	-0.246	***0.869***
Itga11	integrin, alpha 11	-0.256	0.045	0.084	***0.649***	-0.101	***0.606***
Pdlim7	PDZ and LIM domain 7	-0.256	0.042	0.032	***0.579***	-0.118	***0.745***
Adamts2	ADAM metallopeptidase with thrombospondin type 1 motif, 2	-0.257	0.038	0.186	***0.753***	0.083	***0.489***
Gsg1	germ cell associated 1	-0.257	0.018	-0.110	***0.664***	0.254	***0.527***
LOC363337	similar to RIKEN cDNA 1700081O22	-0.257	0.030	-0.194	***0.487***	-0.375	0.015
Igfbp2	insulin-like growth factor binding protein 2	-0.258	0.010	0.276	***0.954***	0.128	***0.779***
Kbtbd8	kelch repeat and BTB (POZ) domain containing 8	-0.258	0.049	-0.224	***0.909***	-0.349	0.017
LOC100302465	hypothetical LOC100302465	-0.259	0.048	-0.188	***0.661***	-0.308	0.025
Lss	lanosterol synthase (2,3-oxidosqualene-lanosterol cyclase)	-0.260	0.014	0.066	***0.705***	0.056	***0.597***
Slc15a3	solute carrier family 15, member 3	-0.262	0.021	0.338	***0.693***	0.417	0.025
Grb14	growth factor receptor bound protein 14	-0.262	0.050	-0.165	***0.764***	-0.103	***0.711***
Jph2	junctophilin 2	-0.263	0.041	-0.073	***0.778***	-0.170	***0.895***
Calb2	calbindin 2	-0.263	0.029	0.217	***0.915***	-0.059	***0.634***
LOC100363170	rCG53134-like	-0.263	0.036	-0.186	***0.346***	-0.462	0.005
Nat2	N-acetyltransferase 2	-0.264	0.026	-0.419	0.001	0.071	0.015
LOC689755	hypothetical protein LOC689755	-0.264	0.039	-0.497	0.005	-0.409	0.008
Scin	scinderin	-0.264	0.045	-0.188	***0.833***	0.166	***0.911***
Ifitm3	interferon induced transmembrane protein 3	-0.267	0.050	0.136	***0.882***	-0.061	***0.719***
Gal	galanin prepropeptide	-0.267	0.024	-0.018	***0.501***	-0.105	***0.736***
Napsa	napsin A aspartic peptidase	-0.269	0.024	0.363	0.004	0.394	0.001
Higd1a	HIG1 hypoxia inducible domain family, member 1A	-0.273	0.040	-0.021	0.044	0.026	***0.126***
Lat	linker for activation of T cells	-0.274	0.004	0.315	***0.074***	0.735	0.006
MGC114520	similar to hypothetical protein E230019M04	-0.274	0.044	-0.353	0.025	-0.130	***0.864***
Cmtm3	CKLF-like MARVEL transmembrane domain containing 3	-0.275	0.043	0.098	***0.809***	0.085	***0.863***
Cd99l2	CD99 molecule-like 2	-0.276	0.042	-0.215	***0.817***	0.011	***0.370***

Dact3	dapper, antagonist of beta-catenin, homolog 3 (Xenopus laevis)	-0.277	0.049	-0.058	***0.512***	-0.098	***0.404***
Pgcp	plasma glutamate carboxypeptidase	-0.278	0.047	0.136	***0.755***	0.010	***0.631***
Reep1	receptor accessory protein 1	-0.279	0.021	-0.001	***0.562***	-0.113	***0.852***
Nefm	neurofilament, medium polypeptide	-0.280	0.028	0.186	***0.743***	0.141	***0.783***
Abcg3l3	ATP-binding cassette, subfamily G (WHITE), member 3-like 3	-0.280	0.024	-0.090	***0.532***	0.299	0.031
Trim30	tripartite motif-containing 30	-0.281	0.004	-0.097	***0.323***	0.164	***0.099***
RGD1306208	hypothetical LOC296483	-0.282	0.049	-0.195	***0.784***	-0.123	***0.868***
Polr1a	polymerase (RNA) I polypeptide A	-0.284	0.039	-0.107	***0.451***	0.140	***0.090***
Bdkrb1	bradykinin receptor B1	-0.285	0.049	-0.203	***0.897***	-0.381	0.016
Gbp5	guanylate binding protein 5	-0.285	0.038	0.003	***0.423***	0.033	***0.334***
Dnajb5	DnaJ (Hsp40) homolog, subfamily B, member 5	-0.286	0.040	-0.169	***0.455***	-0.469	0.015
Olfml3	olfactomedin-like 3	-0.286	0.027	0.200	***0.909***	0.170	***0.912***
Lst1	leukocyte specific transcript 1	-0.287	0.006	0.221	***0.065***	0.540	0.008
Pkib	protein kinase (cAMP-dependent, catalytic) inhibitor beta	-0.287	0.011	-0.229	***0.877***	0.135	***0.240***
RGD1310166	similar to Chromodomain-helicase-DNA-binding protein 1 (CHD-1)	-0.287	0.020	-0.175	***0.309***	0.288	0.011
LOC100362200	olfactory receptor 10-like	-0.288	0.047	-0.223	***0.956***	-0.209	***0.941***
Rit2	Ras-like without CAAX 2	-0.288	0.034	0.095	***0.564***	0.121	***0.689***
Fhl1	four and a half LIM domains 1	-0.288	0.031	-0.081	***0.457***	-0.439	0.009
Wbp5	WW domain binding protein 5	-0.288	0.048	-0.193	***0.833***	-0.128	***0.700***
Rab6b	RAB6B, member RAS oncogene family	-0.290	0.041	0.144	***0.815***	-0.034	***0.449***
Mmp3	matrix metallopeptidase 3	-0.291	0.045	-0.357	0.027	-0.236	***0.793***
Cd300lf	Cd300 molecule-like family member F	-0.291	0.036	-0.185	***0.865***	-0.134	***0.822***
Myl12b	myosin, light chain 12B, regulatory	-0.291	0.038	0.025	***0.595***	-0.142	***0.735***
Gfpt1	glutamine fructose-6-phosphate transaminase 1	-0.292	0.045	-0.354	0.023	-0.148	***0.804***
Igfbp6	insulin-like growth factor binding protein 6	-0.293	0.019	-0.251	***0.731***	-0.311	***0.918***
Ctsk	cathepsin K	-0.294	0.046	0.003	***0.618***	-0.150	***0.938***
Eaf2	ELL associated factor 2	-0.295	0.024	0.282	***0.440***	0.823	0.004
LOC684280	similar to Class I histocompatibility antigen, Non-RT1.A alpha-1 chain precursor	-0.295	0.037	-0.243	0.012	-0.250	***0.746***
Cacna1c	calcium channel, voltage-dependent, L type, alpha 1C subunit	-0.295	0.046	0.002	***0.641***	-0.085	***0.657***
Chchd6	coiled-coil-helix-coiled-coil-helix domain containing 6	-0.296	0.047	-0.185	***0.921***	-0.196	***0.911***
Cplx1	complexin 1	-0.296	0.048	0.045	***0.657***	-0.039	***0.583***
Myoc	myocilin	-0.296	0.040	0.084	***0.520***	0.136	***0.604***
Glrx1	glutaredoxin 1	-0.297	0.049	-0.094	***0.450***	0.029	***0.359***
Rassf6	Ras association (RalGDS/AF-6) domain family member 6	-0.297	0.048	-0.325	0.010	-0.175	***0.571***
Sparc	secreted protein, acidic, cysteine-rich (osteonectin)	-0.298	0.038	0.147	***0.737***	-0.059	***0.568***
Bri3bp	Bri3 binding protein	-0.298	0.027	-0.252	***0.925***	-0.127	***0.820***
LOC690626	hypothetical protein LOC690626	-0.298	0.047	-0.003	***0.203***	-0.139	***0.389***
Rem1	RAS (RAD and GEM)-like GTP-binding 1	-0.298	0.036	-0.139	***0.894***	-0.259	***0.966***
Chrna6	cholinergic receptor, nicotinic, alpha 6	-0.298	0.049	-0.066	***0.662***	0.014	***0.389***
Socs3	suppressor of cytokine signaling 3	-0.298	0.035	-0.441	0.013	-0.443	0.010
Btnl7	butyrophilin-like 7	-0.299	0.047	0.196	***0.435***	0.487	0.003
Pcolce	procollagen C-endopeptidase enhancer	-0.299	0.042	0.161	***0.748***	-0.027	***0.517***
Alox5	arachidonate 5-lipoxygenase	-0.300	0.042	0.065	***0.611***	0.107	***0.747***
Slc3a1	solute carrier family 3, member 1	-0.300	0.046	-0.021	***0.266***	-0.212	***0.053***
Sstr2	somatostatin receptor 2	-0.300	0.036	-0.619	0.001	-0.463	0.006
Ass1	argininosuccinate synthase 1	-0.300	0.018	-0.375	0.018	-0.342	0.016
Sstr3	somatostatin receptor 3	-0.301	0.008	0.359	0.016	0.623	0.007
Csrp1	cysteine and glycine-rich protein 1	-0.302	0.046	-0.035	***0.689***	-0.159	***0.875***
Gprc5a	G protein-coupled receptor, family C, group 5, member A	-0.302	0.032	0.006	***0.521***	0.199	***0.708***
Sgca	sarcoglycan, alpha (dystrophin-associated glycoprotein)	-0.302	0.009	0.518	0.011	0.346	***0.068***
Gpr65	G-protein coupled receptor 65	-0.303	0.036	0.077	***0.355***	0.322	0.021
Plk3	polo-like kinase 3	-0.305	0.045	-0.234	***0.899***	-0.431	0.007
LOC680590	hypothetical protein LOC680590	-0.305	0.049	0.021	***0.665***	-0.245	***0.949***
Cyp7b1	cytochrome P450, family 7, subfamily b, polypeptide 1	-0.305	0.039	0.071	***0.819***	-0.053	***0.701***
Pth	parathyroid hormone	-0.306	0.048	-0.215	***0.607***	-0.335	0.025

Als2cr8	amyotrophic lateral sclerosis 2 (juvenile) chromosome region, candidate 8	-0.306	0.045	-0.122	***0.888***	-0.002	***0.585***
Gnat3	guanine nucleotide binding protein, alpha transducing 3	-0.307	0.024	0.118	***0.147***	-0.063	***0.441***
Rerg	RAS-like, estrogen-regulated, growth-inhibitor	-0.307	0.045	-0.325	0.025	-0.274	***0.964***
Krt23	keratin 23 (histone deacetylase inducible)	-0.307	0.030	0.067	***0.453***	0.004	***0.545***
Zfp503	zinc finger protein 503	-0.307	0.037	0.004	***0.507***	-0.060	***0.570***
Sdf2l1	stromal cell-derived factor 2-like 1	-0.308	0.047	-0.074	***0.725***	0.041	***0.560***
Gucy1b3	guanylate cyclase 1, soluble, beta 3	-0.308	0.035	-0.171	***0.599***	-0.154	***0.419***
LOC502684	hypothetical protein LOC502684	-0.308	0.050	-0.064	***0.712***	-0.004	***0.517***
Rnf152	ring finger protein 152	-0.308	0.045	-0.591	0.001	-0.161	***0.466***
LOC688452	hypothetical protein LOC688452	-0.308	0.032	0.169	***0.751***	0.077	***0.497***
Gfra3	GDNF family receptor alpha 3	-0.309	0.031	-0.006	***0.507***	-0.037	***0.545***
Gnpnat1	glucosamine-phosphate N-acetyltransferase 1	-0.310	0.049	-0.285	0.034	-0.317	0.027
Cenpt	centromere protein T	-0.310	0.003	-0.553	0.001	-0.688	0.000
Nat5	N-acetyltransferase 5	-0.310	0.047	-0.218	***0.795***	-0.131	***0.645***
Cald1	caldesmon 1	-0.310	0.044	-0.103	***0.821***	-0.032	***0.567***
Dbil5	diazepam binding inhibitor-like 5	-0.311	0.049	-0.190	***0.957***	0.000	***0.495***
Lum	lumican	-0.311	0.014	0.084	***0.622***	-0.100	***0.698***
Abcc6	ATP-binding cassette, subfamily C (CFTR/MRP), member 6	-0.312	0.041	-0.169	***0.884***	-0.055	***0.580***
C1s	complement component 1, s subcomponent	-0.312	0.049	0.053	***0.611***	0.230	***0.962***
Fermt3	fermitin family member 3	-0.313	0.043	-0.098	***0.488***	0.206	***0.620***
Ppyr1	pancreatic polypeptide receptor 1	-0.314	0.028	-0.081	***0.624***	0.013	***0.467***
RGD1306565	similar to apoptosis signal-regulating kinase 1	-0.314	0.043	-0.339	0.030	-0.034	***0.415***
Sgcd	sarcoglycan, delta (dystrophin-associated glycoprotein)	-0.314	0.039	0.012	***0.226***	0.078	***0.374***
Tmem17	transmembrane protein 17	-0.315	0.037	-0.169	***0.891***	-0.143	***0.868***
Slc1a2	solute carrier family 1 (glial high affinity glutamate transporter), member 2	-0.315	0.030	-0.349	0.034	-0.243	***0.790***
Ccl20	chemokine (C-C motif) ligand 20	-0.316	0.025	0.441	0.020	0.526	0.001
Vom2r38	vomeronasal 2 receptor, 38	-0.317	0.039	1.642	0.021	2.350	0.007
Psmb8	proteasome (prosome, macropain) subunit, beta type 8 (large multifunctional peptidase 7)	-0.317	0.037	0.175	***0.574***	0.245	***0.396***
Rnase6	ribonuclease, RNase A family, 6	-0.318	0.046	0.109	***0.319***	0.326	***0.815***
Olr813	olfactory receptor 813	-0.318	0.025	-0.468	0.006	-0.207	***0.758***
Dcun1d2	DCN1, defective in cullin neddylation 1, domain containing 2 (S. cerevisiae)	-0.319	0.045	-0.224	***0.861***	-0.060	***0.473***
Krt33a	keratin 33A	-0.319	0.048	-0.263	***0.614***	-0.308	***0.665***
Shbg	sex hormone binding globulin	-0.320	0.044	0.129	***0.623***	0.235	***0.754***
Guca1b	guanylate cyclase activator 1B	-0.320	0.039	-0.084	***0.440***	-0.124	***0.548***
Pgf	placental growth factor	-0.321	0.040	0.055	***0.496***	0.168	***0.768***
Bex2	brain expressed X-linked 2	-0.321	0.044	-0.011	***0.653***	-0.077	***0.765***
Gal	galanin prepropeptide	-0.321	0.014	-0.089	***0.537***	-0.105	***0.587***
Itgad	integrin, alpha D	-0.322	0.047	0.177	***0.411***	0.421	0.018
Siah2	seven in absentia 2	-0.323	0.044	-0.175	***0.751***	-0.292	***0.908***
Mrps10	mitochondrial ribosomal protein S10	-0.323	0.026	-0.103	0.003	-0.160	0.016
Oasl2	2'-5' oligoadenylate synthetase-like 2	-0.323	0.048	0.040	***0.639***	0.162	***0.856***
Scly	selenocysteine lyase	-0.323	0.049	-0.284	***0.939***	-0.235	***0.962***
LOC362795	immunoglobulin G heavy chain	-0.323	0.003	1.130	0.000	1.016	0.000
Cryl1	crystallin, lambda 1	-0.324	0.039	-0.397	0.015	-0.303	***0.986***
Kmo	kynurenine 3-monooxygenase (kynurenine 3-hydroxylase)	-0.324	0.039	-0.029	***0.587***	0.100	***0.607***
Ptprz1	protein tyrosine phosphatase, receptor-type, Z polypeptide 1	-0.324	0.040	0.215	***0.870***	0.064	***0.708***
Fbln5	fibulin 5	-0.324	0.040	0.062	***0.512***	0.038	***0.546***
Col1a2	collagen, type I, alpha 2	-0.325	0.019	0.203	***0.511***	-0.091	***0.419***
Mrps21	mitochondrial ribosomal protein S21	-0.325	0.043	0.034	***0.592***	-0.081	***0.780***
LOC685617	similar to spermatogenesis associated glutamate (E)-rich protein 4b	-0.325	0.040	0.129	***0.478***	-0.131	***0.524***
Gpr171	G protein-coupled receptor 171	-0.325	0.015	0.177	***0.664***	0.202	***0.555***
LOC171573	spleen protein 1 precursor	-0.325	0.045	-0.050	***0.474***	-0.173	***0.695***
Wnt5b	wingless-type MMTV integration site family, member 5B	-0.325	0.040	-0.010	***0.611***	-0.061	***0.689***
Gng8	guanine nucleotide binding protein (G protein), gamma 8	-0.326	0.038	0.146	***0.868***	0.001	***0.629***

Rb1	retinoblastoma 1	-0.326	0.042	-0.050	***0.384***	-0.098	***0.418***
Nol3	nucleolar protein 3 (apoptosis repressor with CARD domain)	-0.326	0.032	0.004	***0.554***	-0.158	***0.876***
LOC685636	hypothetical protein LOC685636	-0.327	0.048	0.107	***0.545***	0.100	***0.815***
Ctps	CTP synthase	-0.327	0.046	-0.027	***0.586***	-0.097	***0.654***
Gyltl1b	glycosyltransferase-like 1B	-0.328	0.044	-0.185	***0.829***	-0.151	***0.795***
Ppy	pancreatic polypeptide	-0.328	0.050	-0.640	0.001	-0.131	***0.744***
Hebp1	heme binding protein 1	-0.328	***0.065***	-0.091	***0.693***	-0.100	***0.687***
Akr1c19	aldo-keto reductase family 1, member C19	-0.328	0.039	-0.210	***0.473***	-0.074	***0.225***
Pira2	paired-Ig-like receptor A2	-0.328	0.041	0.167	***0.830***	0.073	***0.616***
Vip	vasoactive intestinal peptide	-0.329	0.038	-0.342	0.018	-0.191	***0.639***
Batf3	basic leucine zipper transcription factor, ATF-like 3	-0.329	0.018	0.019	***0.487***	0.242	***0.635***
Postn	periostin, osteoblast specific factor	-0.329	0.043	0.015	***0.311***	0.133	***0.635***
Olfml1	olfactomedin-like 1	-0.329	0.044	0.051	***0.700***	-0.092	***0.805***
Vom1r38	vomeronasal 1 receptor 38	-0.329	0.047	-0.455	0.003	-0.288	***0.925***
LOC690576	similar to Discs large homolog 5 (Placenta and prostate DLG) (Discs large protein P-dlg)	-0.329	0.042	0.074	***0.646***	-0.123	***0.586***
Catsperg1	cation channel, sperm-associated, gamma 1	-0.330	0.047	0.193	***0.785***	0.287	***0.051***
Sh3bgr	SH3 domain binding glutamic acid-rich protein	-0.330	0.036	-0.088	***0.438***	-0.124	***0.494***
Cadps	Ca++-dependent secretion activator	-0.330	0.050	-0.007	***0.574***	-0.024	***0.499***
Cspg4	chondroitin sulfate proteoglycan 4	-0.331	0.047	0.205	***0.884***	0.009	***0.331***
Cdc42ep1	CDC42 effector protein (Rho GTPase binding) 1	-0.331	0.034	-0.225	***0.922***	-0.500	0.003
Ces1e	carboxylesterase 1E	-0.332	0.050	0.005	***0.341***	-0.471	0.005
Apobr	apolipoprotein B receptor	-0.332	0.050	0.154	***0.556***	0.235	0.045
Rgs7bp	regulator of G-protein signaling 7 binding protein	-0.332	0.036	-0.006	***0.531***	-0.033	***0.551***
RGD1564776	similar to ornithine decarboxylase-like protein	-0.332	0.041	-0.072	***0.650***	-0.233	***0.915***
Hcst	hematopoietic cell signal transducer	-0.332	0.007	0.248	***0.056***	0.511	0.003
RGD1565203	similar to ESO3 protein	-0.332	0.043	-0.072	***0.825***	0.013	***0.681***
Sh2d7	SH2 domain containing 7	-0.332	0.035	0.139	***0.521***	0.031	***0.540***
Apob	apolipoprotein B	-0.333	0.026	-0.593	0.002	-0.390	0.011
Fads6	fatty acid desaturase domain family, member 6	-0.334	0.050	-0.007	***0.691***	0.001	***0.638***
Ndufs4	NADH dehydrogenase (ubiquinone) Fe-S protein 4	-0.334	0.044	-0.146	***0.757***	-0.226	***0.873***
Afap1	actin filament associated protein 1	-0.334	0.049	-0.015	***0.493***	-0.027	***0.389***
Olr59	olfactory receptor 59	-0.334	0.050	0.385	0.019	0.626	0.001
Slc14a2	solute carrier family 14 (urea transporter), member 2	-0.334	0.042	-0.228	0.038	0.132	0.003
Tnfrsf14	tumor necrosis factor receptor superfamily, member 14	-0.334	0.040	0.102	***0.680***	0.176	***0.939***
Oasl	2'-5'-oligoadenylate synthetase-like	-0.335	0.010	-0.564	0.001	-0.321	***0.912***
Nup210l	nucleoporin 210-like	-0.335	0.042	-0.298	0.030	0.007	***0.561***
RGD1565785	similar to chromosome X open reading frame 21	-0.335	0.020	-0.055	***0.549***	0.176	***0.471***
Try10	trypsin 10	-0.335	0.010	0.202	0.001	0.207	0.003
Mpdz	multiple PDZ domain protein	-0.335	0.043	-0.055	***0.546***	-0.048	***0.456***
Ppap2a	phosphatidic acid phosphatase type 2A	-0.335	0.044	-0.039	***0.736***	-0.145	***0.892***
Trps1	trichorhinophalangeal syndrome I	-0.336	0.048	-0.028	***0.577***	-0.115	***0.733***
Frzb	frizzled-related protein	-0.336	0.030	-0.064	***0.699***	-0.020	***0.594***
Prph	peripherin	-0.336	0.019	0.065	***0.621***	0.024	***0.421***
RGD1311084	similar to 1700113K14Rik protein	-0.336	0.039	0.166	***0.301***	-0.004	***0.556***
Hip1	huntingtin interacting protein 1	-0.336	0.047	0.110	***0.744***	0.056	***0.583***
Tnfaip6	tumor necrosis factor alpha induced protein 6	-0.336	0.035	-0.275	***0.910***	-0.499	0.003
Xcr1	chemokine (C motif) receptor 1	-0.337	0.033	-0.246	***0.886***	-0.029	***0.529***
Cel	carboxyl ester lipase	-0.337	0.047	-0.135	***0.824***	-0.079	***0.665***
Mgp	matrix Gla protein	-0.337	0.023	0.298	***0.829***	-0.052	***0.486***
Cmpk1	cytidine monophosphate (UMP-CMP) kinase 1	-0.338	0.035	-0.335	0.017	-0.134	***0.566***
Gpr33	G protein-coupled receptor 33	-0.338	0.050	-0.048	***0.733***	-0.041	***0.681***
LOC685226	similar to spermatogenesis associated glutamate (E)-rich protein 4d	-0.338	0.033	0.109	***0.301***	-0.204	***0.514***
Prkar1b	protein kinase, cAMP dependent regulatory, type I, beta	-0.338	0.039	0.035	***0.671***	-0.038	***0.609***
Sntg2	syntrophin, gamma 2	-0.338	0.024	-0.060	***0.650***	-0.211	***0.875***
Tex9	testis expressed 9	-0.338	0.037	-0.333	0.027	-0.102	***0.812***
Calr	calreticulin	-0.338	***0.060***	-0.178	***0.684***	-0.156	***0.809***
Ces2i	carboxylesterase 2I	-0.338	0.045	-0.245	0.030	-0.173	***0.847***
LOC100362690	rCG64164-like	-0.338	0.046	-0.234	***0.903***	-0.196	***0.864***
Slc12a8	solute carrier family 12 (potassium/chloride transporters), member 8	-0.339	0.039	-0.485	0.002	-0.252	0.027

Dpep2	dipeptidase 2	-0.339	0.039	-0.141	***0.898***	-0.039	***0.752***
Dnajb11	DnaJ (Hsp40) homolog, subfamily B, member 11	-0.339	0.048	-0.070	***0.651***	-0.096	***0.733***
Olr832	olfactory receptor 832	-0.339	0.026	-0.103	***0.703***	-0.160	***0.665***
Spcs3	signal peptidase complex subunit 3 homolog (S. cerevisiae)	-0.339	0.043	-0.240	***0.795***	-0.220	***0.734***
Gpr20	G protein-coupled receptor 20	-0.339	0.049	-0.242	***0.837***	-0.313	***0.941***
RGD1565166	similar to MGC45438 protein	-0.339	0.038	-0.697	0.001	-0.501	0.006
Casp12	caspase 12	-0.340	0.042	0.018	***0.519***	-0.220	***0.960***
Abcg4	ATP-binding cassette, subfamily G (WHITE), member 4	-0.340	0.028	0.224	***0.905***	0.041	***0.411***
Tmem14a	transmembrane protein 14A	-0.341	0.048	0.058	***0.292***	0.055	***0.585***
Fdxacb1	ferredoxin-fold anticodon binding domain containing 1	-0.341	0.039	-0.361	0.023	-0.226	***0.751***
Slc7a3	solute carrier family 7 (cationic amino acid transporter, y+ system), member 3	-0.341	0.043	0.146	***0.825***	0.373	0.037
Lrp5	low density lipoprotein receptor-related protein 5	-0.342	0.037	-0.375	0.024	-0.360	0.027
RGD1307537	similar to RIKEN cDNA 4933417A18	-0.342	0.037	-0.347	0.027	-0.239	***0.969***
Vom1r45	vomeronasal 1 receptor 45	-0.342	0.036	-0.435	0.011	-0.370	0.008
Cxcr3	chemokine (C-X-C motif) receptor 3	-0.342	0.036	0.000	***0.496***	0.258	***0.923***
Slc18a2	solute carrier family 18 (vesicular monoamine), member 2	-0.342	0.044	-0.071	***0.399***	-0.204	***0.680***
Thsd7a	thrombospondin, type I, domain containing 7A	-0.342	0.043	-0.052	***0.516***	0.003	***0.559***
Ffar2	free fatty acid receptor 2	-0.343	0.020	-0.440	0.006	-0.043	***0.468***
Tpst1	tyrosylprotein sulfotransferase 1	-0.343	0.046	0.159	***0.828***	0.005	***0.453***
Slc23a3	solute carrier family 23 (nucleobase transporters), member 3	-0.343	0.050	-0.393	0.020	-0.083	***0.514***
Flna	filamin A, alpha	-0.343	0.032	0.111	***0.878***	-0.084	***0.661***
LOC688302	similar to spermatogenesis associated glutamate (E)-rich protein 4d	-0.344	0.019	-0.322	0.021	-0.462	0.005
Ces1e	carboxylesterase 1E	-0.344	0.038	0.099	***0.606***	-0.473	0.004
Fam180a	family with sequence similarity 180, member A	-0.344	0.049	0.277	***0.873***	0.378	0.015
Zfp703	zinc finger protein 703	-0.344	0.038	-0.334	0.014	-0.307	***0.984***
Hes6	hairy and enhancer of split 6 (Drosophila)	-0.344	0.042	-0.262	***0.985***	-0.153	***0.901***
Lrrn4cl	LRRN4 C-terminal like	-0.344	0.029	-0.003	***0.454***	-0.144	***0.680***
Adh4	alcohol dehydrogenase 4 (class II), pi polypeptide	-0.345	0.020	-0.389	0.006	0.031	***0.139***
Cyp4f18	cytochrome P450, family 4, subfamily f, polypeptide 18	-0.345	0.046	0.227	***0.949***	0.329	0.039
Gls	glutaminase	-0.345	0.041	-0.312	0.016	-0.385	0.015
Bgn	biglycan	-0.345	0.024	0.246	***0.894***	0.128	***0.827***
Card9	caspase recruitment domain family, member 9	-0.345	0.036	0.098	***0.613***	0.132	***0.637***
Olr1326	olfactory receptor 1326	-0.345	0.039	0.105	***0.765***	0.016	***0.465***
Ptgs1	prostaglandin-endoperoxide synthase 1	-0.345	0.040	0.103	***0.624***	0.141	***0.632***
Sec16b	SEC16 homolog B (S. cerevisiae)	-0.345	0.027	-0.584	0.001	-0.594	0.001
Fermt2	fermitin family member 2	-0.346	0.036	-0.029	***0.366***	-0.251	***0.740***
Gpx4	glutathione peroxidase 4	-0.346	0.039	-0.123	***0.863***	-0.145	***0.902***
Msra	methionine sulfoxide reductase A	-0.346	0.033	-0.002	***0.298***	-0.149	***0.773***
Igf1	insulin-like growth factor 1	-0.347	0.034	0.429	0.012	0.153	***0.916***
Tnfsf14	tumor necrosis factor (ligand) superfamily, member 14	-0.347	0.019	0.205	***0.482***	0.274	***0.683***
Sigmar1	sigma non-opioid intracellular receptor 1	-0.348	0.049	-0.190	***0.847***	-0.210	***0.922***
Lrrn4cl	LRRN4 C-terminal like	-0.348	0.040	-0.115	***0.804***	-0.093	***0.743***
Fbxo23	F-box only protein 23	-0.348	0.044	-0.357	0.024	-0.261	***0.965***
Tuba1a	tubulin, alpha 1A	-0.348	0.045	-0.025	***0.502***	-0.191	***0.827***
Vmp1	vacuole membrane protein 1	-0.348	0.048	0.020	***0.452***	-0.025	***0.539***
Wisp1	WNT1 inducible signaling pathway protein 1	-0.348	0.027	0.108	***0.677***	-0.028	***0.489***
RGD1311874	hypothetical LOC300751	-0.349	0.001	-1.537	0.000	-2.249	0.000
Pde4b	phosphodiesterase 4B, cAMP specific	-0.349	0.045	-0.333	***0.958***	-0.465	0.004
Txlnb	taxilin beta	-0.349	0.037	-0.461	0.009	-0.370	0.019
Xcl1	chemokine (C motif) ligand 1	-0.349	0.032	0.130	***0.688***	0.257	***0.056***
Tmsb10	thymosin, beta 10	-0.349	0.025	-0.208	***0.850***	-0.113	***0.799***
Ifit2	interferon-induced protein with tetratricopeptide repeats 2	-0.349	0.045	-0.185	***0.933***	-0.229	***0.960***
S100z	S100 calcium binding protein Z	-0.349	0.029	-0.322	***0.929***	-0.332	0.023
Pcbp3	poly(rC) binding protein 3	-0.349	0.038	0.036	***0.610***	-0.122	***0.706***
Galnt3	UDP-N-acetyl-alpha-D-galactosamine:polypeptide N-acetylgalactosaminyltransferase 3 (GalNAc-T3)	-0.349	0.038	-0.581	0.001	-0.305	***0.957***
RGD1564257	similar to hypothetical protein FLJ32825	-0.350	0.038	0.102	***0.697***	0.018	***0.687***

Prodh	proline dehydrogenase	-0.350	0.020	-0.034	***0.325***	-0.381	0.012
Kynu	kynureninase	-0.350	0.005	-0.482	0.001	-0.062	***0.056***
Mvd	mevalonate (diphospho) decarboxylase	-0.350	0.020	-0.195	***0.858***	0.015	***0.454***
Tspan12	tetraspanin 12	-0.350	0.043	-0.302	0.034	-0.427	0.010
Sim2	single-minded homolog 2 (Drosophila)	-0.351	0.048	-0.242	***0.833***	-0.176	***0.681***
Echdc2	enoyl CoA hydratase domain containing 2	-0.351	0.043	-0.037	***0.643***	-0.270	***0.933***
Nedd4	neural precursor cell expressed, developmentally down-regulated 4	-0.351	0.038	0.025	***0.452***	-0.057	***0.331***
Cdhr4	cadherin-related family member 4	-0.352	0.026	0.411	0.040	0.325	***0.054***
Cpne7	copine VII	-0.352	0.031	0.295	***0.940***	0.197	***0.835***
Spink5	serine peptidase inhibitor, Kazal type 5	-0.352	0.035	0.042	***0.323***	-0.082	***0.426***
Tnc	tenascin C	-0.352	0.022	-0.275	***0.744***	-0.427	0.010
Glt8d2	glycosyltransferase 8 domain containing 2	-0.353	0.034	-0.014	***0.536***	-0.313	0.016
Slc4a4	solute carrier family 4, sodium bicarbonate cotransporter, member 4	-0.353	0.025	-0.464	0.001	0.015	0.024
Mitf	microphthalmia-associated transcription factor	-0.353	0.040	-0.119	***0.770***	-0.157	***0.791***
Il11ra1	interleukin 11 receptor, alpha chain 1	-0.354	0.031	0.040	***0.677***	-0.070	***0.749***
Mxra8	matrix-remodelling associated 8	-0.354	0.035	0.082	***0.768***	0.052	***0.667***
Qsox2	quiescin Q6 sulfhydryl oxidase 2	-0.354	0.039	-0.247	***0.905***	-0.187	***0.848***
Cntnap1	contactin associated protein 1	-0.354	0.039	0.166	***0.895***	0.090	***0.816***
LOC689796	hypothetical protein LOC689796	-0.355	0.032	-0.394	0.020	-0.363	0.025
Maged1	melanoma antigen, family D, 1	-0.355	0.041	-0.320	0.024	-0.248	***0.682***
Rpp25	ribonuclease P 25 subunit (human)	-0.355	0.034	-0.166	***0.838***	-0.218	***0.871***
Hrasls	HRAS-like suppressor	-0.355	0.036	-0.146	***0.515***	-0.157	***0.667***
Ccz1	CCZ1 vacuolar protein trafficking and biogenesis associated homolog (S. cerevisiae)	-0.356	0.045	-0.330	0.034	-0.068	***0.634***
Nit2	nitrilase family, member 2	-0.356	0.009	-0.042	***0.293***	-0.087	***0.294***
Psbpc2	prostatic steroid-binding protein C2	-0.357	0.037	-0.233	***0.903***	-0.002	***0.518***
Vamp1	vesicle-associated membrane protein 1	-0.357	0.032	0.200	***0.946***	0.121	***0.811***
Hsf2bp	heat shock transcription factor 2 binding protein	-0.357	0.043	-0.145	***0.696***	0.114	***0.579***
Hsd17b1	hydroxysteroid (17-beta) dehydrogenase 1	-0.357	0.029	-0.100	***0.860***	-0.204	***0.947***
Lppr3	lipid phosphate phosphatase-related protein type 3	-0.357	0.042	0.108	***0.609***	-0.012	***0.549***
Pdzrn3	PDZ domain containing RING finger 3	-0.358	0.046	0.027	***0.488***	-0.110	***0.657***
Paqr6	progestin and adipoQ receptor family member VI	-0.358	0.049	-0.031	***0.681***	-0.053	***0.733***
Thra	thyroid hormone receptor alpha	-0.358	0.050	-0.087	***0.734***	0.044	***0.618***
Npr1	natriuretic peptide receptor A/guanylate cyclase A (atrionatriuretic peptide receptor A)	-0.358	0.043	0.082	***0.750***	-0.027	***0.500***
Crtap	cartilage associated protein	-0.358	0.029	-0.128	***0.625***	-0.668	0.000
Egfl8	EGF-like-domain, multiple 8	-0.359	0.039	-0.128	***0.926***	-0.180	***0.954***
LOC682105	similar to receptor expression enhancing protein 2	-0.359	0.036	0.254	***0.926***	0.038	***0.541***
Serp1	stress-associated endoplasmic reticulum protein 1	-0.359	0.041	-0.196	***0.541***	-0.117	***0.418***
Lgals9	lectin, galactoside-binding, soluble, 9	-0.359	0.034	-0.197	***0.857***	-0.114	***0.758***
Olr931	olfactory receptor 931	-0.359	0.026	-0.596	0.003	-0.431	0.011
Tesc	tescalcin	-0.359	0.028	-0.163	***0.676***	0.158	***0.198***
Gap43	growth associated protein 43	-0.360	0.038	0.163	***0.811***	-0.006	***0.394***
Rerg	RAS-like, estrogen-regulated, growth-inhibitor	-0.360	0.028	-0.303	0.036	-0.198	***0.874***
Gosr2	golgi SNAP receptor complex member 2	-0.360	0.043	-0.165	***0.889***	-0.081	***0.759***
Smtnl2	smoothelin-like 2	-0.360	0.039	-0.095	***0.753***	-0.113	***0.785***
Sqrdl	sulfide quinone reductase-like (yeast)	-0.360	0.041	-0.240	0.025	-0.028	***0.248***
Ly49i4	Ly49 inhibitory receptor 4	-0.360	0.029	-0.054	***0.279***	-0.164	***0.441***
Galnt11	UDP-N-acetyl-alpha-D-galactosamine:polypeptide N-acetylgalactosaminyltransferase 11 (GalNAc-T11)	-0.360	0.043	-0.037	***0.644***	-0.145	***0.874***
Slc7a14	solute carrier family 7 (cationic amino acid transporter, y+ system), member 14	-0.360	0.019	0.119	***0.882***	0.023	***0.524***
Nrxn2	neurexin 2	-0.361	0.045	-0.209	***0.805***	-0.179	***0.725***
Tlr3	toll-like receptor 3	-0.361	0.039	-0.163	***0.363***	-0.198	***0.460***
Zfp758	zinc finger protein 758	-0.361	0.044	-0.234	***0.808***	-0.259	***0.777***
S1pr5	sphingosine-1-phosphate receptor 5	-0.361	0.042	-0.329	0.029	-0.135	***0.641***
Klhl23	kelch-like 23 (Drosophila)	-0.361	0.047	-0.258	***0.969***	-0.054	***0.531***
Angptl3	angiopoietin-like 3	-0.362	0.044	-0.414	0.015	-0.348	***0.958***
Cldn23	claudin 23	-0.362	0.028	-0.283	0.037	-0.078	***0.401***
Lgals5	lectin, galactose binding, soluble 5	-0.362	0.027	-0.143	***0.736***	-0.152	***0.831***
Cdh11	cadherin 11	-0.362	0.035	0.033	***0.671***	0.051	***0.655***
Rab44	RAB44, member RAS oncogene family	-0.362	0.036	-0.402	0.012	-0.119	***0.193***

Cyp4b1	cytochrome P450, family 4, subfamily b, polypeptide 1	-0.363	0.025	-0.293	0.037	-0.187	***0.496***
Grin1	glutamate receptor, ionotropic, N-methyl D-aspartate 1	-0.363	0.039	-0.156	***0.909***	0.008	***0.403***
Pla2g7	phospholipase A2, group VII (platelet-activating factor acetylhydrolase, plasma)	-0.363	0.040	-0.120	***0.892***	-0.028	***0.493***
Ptpn7	protein tyrosine phosphatase, non-receptor type 7	-0.363	0.014	0.323	0.028	0.514	0.003
Ptprd	protein tyrosine phosphatase, receptor type, D	-0.363	0.025	-0.347	0.021	-0.117	***0.432***
Smpd4	sphingomyelin phosphodiesterase 4, neutral membrane	-0.363	0.021	0.018	***0.283***	-0.454	0.005
Slc8a1	solute carrier family 8 (sodium/calcium exchanger), member 1	-0.363	0.042	0.125	***0.775***	0.153	***0.775***
LOC685577	similar to major urinary protein 5	-0.363	0.022	-0.150	***0.893***	-0.161	***0.935***
Prss12	protease, serine, 12 neurotrypsin (motopsin)	-0.364	0.041	-0.167	***0.741***	-0.065	***0.432***
Pi16	peptidase inhibitor 16	-0.364	0.040	-0.207	***0.785***	-0.252	***0.833***
Cpb2	carboxypeptidase B2 (plasma)	-0.365	0.040	-0.308	***0.962***	-0.292	***0.953***
LOC680758	similar to spermatogenesis associated glutamate (E)-rich protein 4d	-0.365	0.037	-0.133	***0.677***	-0.317	***0.925***
Sycp2	synaptonemal complex protein 2	-0.365	0.034	-0.093	***0.842***	-0.034	***0.751***
Pcp4l1	Purkinje cell protein 4-like 1	-0.365	0.029	-0.047	***0.676***	-0.257	***0.951***
Npl	N-acetylneuraminate pyruvate lyase	-0.365	0.041	0.058	***0.601***	-0.025	***0.432***
Sepx1	selenoprotein X, 1	-0.365	0.039	0.064	***0.673***	-0.145	***0.908***
Slc16a12	solute carrier family 16, member 12 (monocarboxylic acid transporter 12)	-0.365	0.038	-0.240	***0.972***	-0.269	***0.964***
Ak1	adenylate kinase 1	-0.366	0.036	-0.025	***0.346***	-0.006	***0.395***
Rab3c	RAB3C, member RAS oncogene family	-0.366	0.038	-0.124	***0.841***	-0.109	***0.794***
MGC116197	similar to RIKEN cDNA 1700001E04	-0.366	0.030	0.160	***0.742***	0.137	***0.821***
Tubb2a	tubulin, beta 2A class IIa	-0.366	0.042	-0.212	***0.907***	-0.405	0.013
Prlr	prolactin receptor	-0.366	0.017	-0.407	0.008	-0.289	0.008
Grip2	glutamate receptor interacting protein 2	-0.367	0.026	-0.078	***0.729***	-0.053	***0.657***
RGD1306001	similar to 2210021J22Rik protein	-0.367	0.025	-0.192	0.039	-0.198	0.026
Ucp3	uncoupling protein 3 (mitochondrial, proton carrier)	-0.367	0.040	-0.231	***0.917***	-0.193	***0.900***
Pcolce	procollagen C-endopeptidase enhancer	-0.367	0.023	0.160	***0.877***	0.059	***0.712***
Coro6	coronin 6	-0.368	0.049	-0.100	***0.522***	-0.057	***0.416***
Mrps2	mitochondrial ribosomal protein S2	-0.368	0.034	-0.116	***0.792***	-0.120	***0.790***
Srd5a1	steroid-5-alpha-reductase, alpha polypeptide 1 (3-oxo-5 alpha-steroid delta 4-dehydrogenase alpha 1)	-0.368	0.037	-0.354	0.006	-0.148	0.033
Chrna10	cholinergic receptor, nicotinic, alpha 10	-0.368	0.046	-0.286	***0.752***	-0.231	***0.589***
Oaf	OAF homolog (Drosophila)	-0.368	0.033	0.054	***0.671***	-0.135	***0.789***
Ppp1r14a	protein phosphatase 1, regulatory (inhibitor) subunit 14A	-0.368	0.041	0.006	***0.596***	-0.012	***0.565***
LOC100363621	hypothetical protein LOC100363621	-0.368	0.029	-0.006	***0.280***	-0.073	***0.323***
Slc2a10	solute carrier family 2 (facilitated glucose transporter), member 10	-0.368	0.040	-0.353	0.009	-0.333	0.021
Egr1	early growth response 1	-0.368	0.022	-0.378	0.011	-0.826	0.000
Hcrtr1	hypocretin (orexin) receptor 1	-0.368	0.033	-0.132	***0.922***	-0.016	***0.625***
Klhdc8b	kelch domain containing 8B	-0.368	0.045	0.126	***0.737***	0.068	***0.629***
Bambi	BMP and activin membrane-bound inhibitor, homolog (Xenopus laevis)	-0.368	0.030	-0.125	***0.913***	0.024	***0.728***
Wfdc15b	WAP four-disulfide core domain 15B	-0.369	0.029	0.075	***0.607***	0.232	***0.929***
Atp1b2	ATPase, Na+/K+ transporting, beta 2 polypeptide	-0.369	0.036	0.251	***0.930***	0.220	***0.941***
LOC686921	hypothetical protein LOC686921	-0.369	0.025	0.139	***0.565***	0.042	***0.384***
Kcnf1	potassium voltage-gated channel, subfamily F, member 1	-0.369	0.031	-0.278	***0.916***	-0.497	0.004
P2rx2	purinergic receptor P2X, ligand-gated ion channel, 2	-0.369	0.026	-0.229	***0.576***	-0.291	***0.680***
Gpx1	glutathione peroxidase 1	-0.369	0.047	-0.054	***0.549***	-0.070	***0.599***
Ly6h	lymphocyte antigen 6 complex, locus H	-0.370	0.048	0.020	***0.413***	-0.268	***0.818***
Lppr4	lipid phosphate phosphatase-related protein type 4	-0.370	0.038	0.026	***0.471***	-0.019	***0.363***
Slc35a2	solute carrier family 35 (UDP-galactose transporter), member A2	-0.370	0.018	0.072	***0.572***	0.024	***0.647***
RGD1562963	similar to chromosome 6 open reading frame 52	-0.371	0.040	-0.427	0.013	-0.354	0.026
Adamdec1	ADAM-like, decysin 1	-0.371	0.014	0.267	***0.953***	0.053	***0.630***

LOC685808	similar to transmembrane NK cell receptor 2B4	-0.371	0.029	0.187	***0.737***	0.365	0.026
Tmem213	transmembrane protein 213	-0.371	0.027	-0.276	***0.911***	-0.140	***0.774***
Creld2	cysteine-rich with EGF-like domains 2	-0.372	0.034	0.109	***0.186***	0.184	***0.803***
Capn6	calpain 6	-0.372	0.012	0.390	0.016	0.132	***0.564***
Kctd15	potassium channel tetramerisation domain containing 15	-0.372	0.028	0.124	***0.794***	-0.170	***0.823***
Slc22a17	solute carrier family 22, member 17	-0.372	0.034	0.154	***0.925***	0.133	***0.898***
LOC689757	similar to osteoclast inhibitory lectin	-0.372	0.033	-0.313	0.035	-0.187	***0.650***
Gpr171	G protein-coupled receptor 171	-0.372	0.008	0.225	***0.516***	0.277	***0.603***
Dab2	disabled homolog 2 (Drosophila)	-0.372	0.041	0.077	***0.554***	0.021	***0.488***
Mdp1	magnesium-dependent phosphatase 1	-0.373	0.032	-0.336	0.031	-0.298	***0.987***
Nsdhl	NAD(P) dependent steroid dehydrogenase-like	-0.373	0.027	-0.187	***0.911***	-0.127	***0.791***
Syt16	synaptotagmin XVI	-0.373	0.028	-0.206	***0.636***	-0.243	***0.640***
Clcf1	cardiotrophin-like cytokine factor 1	-0.373	0.041	-0.175	***0.616***	-0.004	***0.288***
RT1-N1	RT1 class Ib, locus N1	-0.373	0.047	0.085	***0.336***	-0.013	***0.359***
Atp2b3	ATPase, Ca++ transporting, plasma membrane 3	-0.374	0.028	-0.144	***0.564***	-0.342	0.025
Nqo1	NAD(P)H dehydrogenase, quinone 1	-0.374	0.038	0.037	***0.084***	-0.108	***0.247***
Spata24	spermatogenesis associated 24	-0.374	0.037	-0.123	***0.727***	0.070	***0.783***
Ly49i6	Ly49 inhibitory receptor 6	-0.374	0.021	-0.212	***0.830***	0.202	***0.852***
Tm6sf1	transmembrane 6 superfamily member 1	-0.374	0.040	-0.079	***0.714***	-0.022	***0.557***
Gfra2	GDNF family receptor alpha 2	-0.374	0.036	0.080	***0.696***	0.111	***0.748***
Thbs1	thrombospondin 1	-0.374	0.039	-0.380	0.024	-0.577	0.002
Bcat1	branched chain amino acid transaminase 1, cytosolic	-0.375	0.031	0.064	***0.811***	0.059	***0.773***
Ngb	neuroglobin	-0.375	0.029	0.287	0.013	0.141	0.039
RGD1309676	similar to RIKEN cDNA 5730469M10	-0.375	0.035	-0.175	***0.650***	0.000	***0.440***
Trpc1	transient receptor potential cation channel, subfamily C, member 1	-0.375	0.033	-0.332	0.027	-0.482	0.005
Rassf9	Ras association (RalGDS/AF-6) domain family (N-terminal) member 9	-0.375	0.038	-0.134	***0.875***	-0.141	***0.882***
Amica1	adhesion molecule, interacts with CXADR antigen 1	-0.376	0.036	0.213	***0.906***	0.296	0.050
Fa2h	fatty acid 2-hydroxylase	-0.376	0.024	-0.377	0.012	-0.291	***0.661***
Csrnp3	cysteine-serine-rich nuclear protein 3	-0.376	0.030	-0.212	***0.610***	-0.010	***0.215***
Dusp8	dual specificity phosphatase 8	-0.377	0.029	-0.336	0.029	-0.455	0.005
Pde2a	phosphodiesterase 2A, cGMP-stimulated	-0.377	0.026	-0.279	0.035	-0.253	***0.685***
LOC684652	hypothetical protein LOC684652	-0.377	0.017	-0.196	***0.412***	-0.383	0.009
Ly6e	lymphocyte antigen 6 complex, locus E	-0.378	0.038	0.055	***0.311***	0.074	***0.374***
Ppp2r2b	protein phosphatase 2, regulatory subunit B, beta	-0.378	0.029	-0.006	***0.360***	-0.105	***0.521***
Tarbp2	TAR (HIV-1) RNA binding protein 2	-0.378	0.037	-0.432	0.010	-0.303	***0.933***
Dbn1	drebrin 1	-0.378	0.033	0.049	***0.752***	0.009	***0.673***
Fgf13	fibroblast growth factor 13	-0.378	0.014	0.132	***0.669***	0.013	***0.644***
Senp5	Sumo1/sentrin/SMT3 specific peptidase 5	-0.379	0.038	-0.022	***0.264***	-0.185	***0.518***
Hdgfrp3	hepatoma-derived growth factor, related protein 3	-0.379	0.035	-0.139	***0.861***	-0.164	***0.887***
Klrc3	killer cell lectin-like receptor subfamily C, member 3	-0.379	0.032	0.075	***0.746***	0.473	0.005
Hus1	HUS1 checkpoint homolog (S. pombe)	-0.380	0.012	0.468	0.004	0.167	***0.705***
LOC679566	hypothetical protein LOC679566	-0.380	0.021	0.040	***0.099***	-0.164	***0.058***
Mmd	monocyte to macrophage differentiation-associated	-0.380	0.036	-0.111	***0.785***	0.024	***0.539***
Plekhn1	pleckstrin homology domain containing, family N member 1	-0.380	0.026	0.105	***0.684***	0.117	***0.737***
Dcx	doublecortin	-0.380	0.031	0.337	0.012	0.079	***0.521***
Lsp1	lymphocyte-specific protein 1	-0.380	0.019	0.205	***0.801***	0.072	***0.502***
Clec4a1	C-type lectin domain family 4, member A1	-0.381	0.031	-0.040	***0.588***	0.249	***0.954***
Btnl5	butyrophilin-like 5	-0.381	0.026	0.169	***0.339***	0.420	0.007
Mmp10	matrix metallopeptidase 10	-0.381	0.012	0.114	***0.405***	0.557	0.001
Acsm4	acyl-CoA synthetase medium-chain family member 4	-0.382	0.017	-0.087	***0.161***	0.328	0.006
Ipcef1	interactor protein for cytohesin exchange factors 1	-0.382	0.020	-0.269	***0.957***	-0.115	***0.539***
Espl1	extra spindle pole bodies homolog 1 (S. cerevisiae)	-0.382	0.024	-0.451	0.007	-0.221	***0.892***
Gulp1	GULP, engulfment adaptor PTB domain containing 1	-0.382	0.031	-0.082	***0.804***	-0.124	***0.878***
Pcgf1	polycomb group ring finger 1	-0.383	0.025	0.007	***0.589***	0.126	***0.739***
Myef2	myelin expression factor 2	-0.383	0.032	-0.078	***0.728***	-0.185	***0.889***

Pde1b	phosphodiesterase 1B, calmodulin-dependent	-0.383	0.032	-0.029	***0.549***	0.073	***0.503***
RGD1564387	similar to RIKEN cDNA C030014K22 gene	-0.383	0.038	-0.114	***0.739***	-0.188	***0.802***
Gabbr1	gamma-aminobutyric acid (GABA) B receptor 1	-0.384	0.028	0.344	***0.881***	0.176	***0.636***
LOC679566	hypothetical protein LOC679566	-0.384	0.016	0.112	***0.069***	-0.198	0.040
Cmtm8	CKLF-like MARVEL transmembrane domain containing 8	-0.384	0.034	-0.364	0.019	-0.242	***0.863***
Rab5c	RAB5C, member RAS oncogene family	-0.384	0.046	-0.296	0.021	-0.325	***0.907***
RGD1565046	similar to CLM6	-0.384	0.030	-0.039	***0.686***	-0.081	***0.791***
Stx2	syntaxin 2	-0.384	0.033	-0.133	***0.919***	-0.117	***0.837***
Gmds	GDP-mannose 4, 6-dehydratase	-0.384	0.029	-0.422	0.007	-0.225	***0.710***
Tmed3	transmembrane emp24 protein transport domain containing 3	-0.384	0.026	-0.420	0.011	-0.181	***0.812***
Aqp4	aquaporin 4	-0.384	0.008	-0.351	0.008	-0.220	***0.936***
Mmp7	matrix metallopeptidase 7	-0.384	0.032	-0.202	***0.930***	-0.266	***0.966***
Gdnf	glial cell derived neurotrophic factor	-0.385	0.029	0.337	0.011	-0.259	***0.368***
Gria4	glutamate receptor, ionotrophic, AMPA 4	-0.385	0.031	-0.073	***0.673***	-0.080	***0.735***
Sema6d	sema domain, transmembrane domain (TM), and cytoplasmic domain, (semaphorin) 6D	-0.385	0.041	0.132	***0.653***	-0.098	***0.402***
Alox5ap	arachidonate 5-lipoxygenase activating protein	-0.385	0.022	-0.009	***0.671***	0.077	***0.811***
LOC686539	similar to immunoglobulin superfamily containing leucine-rich repeat	-0.385	0.032	-0.087	***0.816***	-0.048	***0.707***
Bok	BCL2-related ovarian killer	-0.385	0.035	-0.422	0.014	-0.297	***0.966***
Higd1b	HIG1 hypoxia inducible domain family, member 1B	-0.385	0.003	-0.325	0.005	-0.484	0.002
Pacrg	Park2 co-regulated	-0.385	0.029	0.209	***0.633***	0.133	***0.570***
Mogat1	monoacylglycerol O-acyltransferase 1	-0.385	0.031	-0.101	***0.752***	-0.160	***0.828***
Rap1gap	Rap1 GTPase-activating protein	-0.386	0.036	-0.490	0.001	-0.336	0.001
Tle2	transducin-like enhancer of split 2 (E(sp1) homolog, Drosophila)	-0.386	0.019	0.164	***0.927***	0.063	***0.618***
Zfp57	zinc finger protein 57	-0.386	0.030	-0.240	***0.898***	-0.313	***0.966***
Il34	interleukin 34	-0.386	0.031	-0.243	***0.905***	-0.297	***0.978***
Mpp4	membrane protein, palmitoylated 4 (MAGUK p55 subfamily member 4)	-0.387	0.035	0.119	***0.787***	0.120	***0.768***
Lrrn2	leucine rich repeat neuronal 2	-0.387	0.028	-0.004	***0.469***	-0.109	***0.698***
Rassf4	Ras association (RalGDS/AF-6) domain family member 4	-0.387	0.018	-0.445	0.005	-0.319	0.021
LOC367390	hypothetical LOC367390	-0.387	0.019	-0.330	0.030	-0.388	0.013
Inhbe	inhibin beta E	-0.387	0.043	-0.416	0.015	-0.087	***0.524***
Sdc2	syndecan 2	-0.387	0.018	0.029	***0.550***	-0.209	***0.894***
Lphn3	latrophilin 3	-0.388	0.037	0.189	***0.761***	0.177	***0.685***
Nsg1	neuron specific gene family member 1	-0.388	0.018	0.044	***0.433***	0.023	***0.554***
Mettl7a	methyltransferase like 7A	-0.388	0.031	-0.208	***0.796***	0.047	***0.370***
Rnf207	ring finger protein 207	-0.388	0.027	-0.047	***0.733***	0.030	***0.726***
LOC498276	Fc gamma receptor II beta	-0.389	0.018	0.180	***0.891***	0.063	***0.604***
Insc	inscuteable homolog (Drosophila)	-0.389	0.019	-0.081	***0.716***	-0.082	***0.723***
Lrrc38	leucine rich repeat containing 38	-0.389	0.035	-0.110	***0.390***	-0.042	***0.398***
Igfbp5	insulin-like growth factor binding protein 5	-0.390	0.007	0.381	0.019	0.092	***0.726***
St3gal3	ST3 beta-galactoside alpha-2,3-sialyltransferase 3	-0.390	0.039	-0.635	0.001	-0.635	0.001
Scn7a	sodium channel, voltage-gated, type VII, alpha	-0.391	0.029	0.050	***0.488***	-0.039	***0.345***
Vom2r5	vomeronasal 2 receptor, 5	-0.391	0.031	-0.112	***0.577***	0.084	***0.244***
Cabp2	calcium binding protein 2	-0.391	0.028	-0.317	0.022	-0.232	***0.895***
RGD1562674	similar to kinase suppressor of ras 2	-0.391	0.043	-0.361	0.036	0.075	***0.148***
Sik1	salt-inducible kinase 1	-0.391	0.021	-0.673	0.001	-0.707	0.001
Crtap	cartilage associated protein	-0.392	0.032	-0.402	0.011	-0.318	***0.911***
Acta2	smooth muscle alpha-actin	-0.392	0.027	-0.115	***0.911***	-0.223	***0.945***
O3far1	omega-3 fatty acid receptor 1	-0.392	0.025	-0.540	0.001	-0.355	0.019
Tlr7	toll-like receptor 7	-0.392	0.028	0.136	***0.683***	0.238	***0.710***
Pxdc1	PX domain containing 1	-0.392	0.019	-0.231	***0.667***	-0.140	***0.481***
Vof16	ischemia related factor vof-16	-0.392	0.038	0.204	***0.055***	0.096	***0.441***
Epgn	epithelial mitogen homolog (mouse)	-0.393	0.033	-0.243	***0.799***	-0.472	0.009
Tmed4	transmembrane emp24 protein transport domain containing 4	-0.393	0.033	-0.278	***0.677***	-0.422	0.008
Myh2	myosin, heavy chain 2, skeletal muscle, adult	-0.393	0.006	-0.534	0.005	-0.648	0.002
Cxcr7	chemokine (C-X-C motif) receptor 7	-0.393	0.018	-0.327	0.029	-0.650	0.000
Epor	erythropoietin receptor	-0.394	0.030	0.277	***0.937***	0.132	***0.703***
Glt8d1	glycosyltransferase 8 domain containing 1	-0.395	0.028	-0.122	***0.890***	-0.029	***0.550***
MGC116197	similar to RIKEN cDNA 1700001E04	-0.395	0.027	0.005	***0.506***	-0.231	***0.937***
RGD1563222	similar to RIKEN cDNA A930018P22	-0.395	0.026	-0.282	***0.939***	-0.005	***0.569***

Mgst1	microsomal glutathione S-transferase 1	-0.395	0.013	-0.046	***0.129***	-0.164	***0.216***
Fam158a	family with sequence similarity 158, member A	-0.395	0.027	-0.186	***0.829***	-0.027	***0.477***
Slc12a8	solute carrier family 12 (potassium/chloride transporters), member 8	-0.396	0.022	-0.540	0.002	-0.280	0.023
Phlda1	pleckstrin homology-like domain, family A, member 1	-0.396	0.019	-0.221	***0.906***	-0.299	***0.951***
Rgc32	response gene to complement 32	-0.396	0.023	-0.365	0.014	-0.374	0.017
RGD1304731	similar to RIKEN cDNA 5330437I02 gene	-0.396	0.011	-0.270	0.023	-0.130	***0.130***
Mfap5	microfibrillar associated protein 5	-0.396	0.004	0.039	***0.430***	-0.157	***0.923***
Ttyh1	tweety homolog 1 (Drosophila)	-0.396	0.032	-0.181	***0.584***	-0.257	***0.760***
Ccnj	cyclin J	-0.397	0.015	-0.125	***0.818***	-0.078	***0.735***
Slco3a1	solute carrier organic anion transporter family, member 3a1	-0.397	0.027	0.033	***0.698***	-0.041	***0.688***
Dtx3	deltex homolog 3 (Drosophila)	-0.397	0.030	0.085	***0.831***	0.040	***0.652***
Sptbn2	spectrin, beta, non-erythrocytic 2	-0.397	0.028	-0.074	***0.734***	0.003	***0.610***
Pla2g1b	phospholipase A2, group IB, pancreas	-0.398	0.019	-0.329	0.012	-0.373	0.010
Tacstd2	tumor-associated calcium signal transducer 2	-0.398	0.025	-0.340	0.017	-0.118	***0.814***
Arhgdig	Rho GDP dissociation inhibitor (GDI) gamma	-0.398	0.031	0.025	***0.538***	-0.139	***0.810***
Smarcd3	SWI/SNF related, matrix associated, actin dependent regulator of chromatin, subfamily d, member 3	-0.398	0.031	0.049	***0.760***	-0.027	***0.514***
Hapln2	hyaluronan and proteoglycan link protein 2	-0.398	0.023	-0.036	0.017	-0.191	***0.605***
Nxnl2	nucleoredoxin-like 2	-0.398	0.033	-0.129	***0.756***	-0.107	***0.685***
Usp18	ubiquitin specific peptidase 18	-0.398	0.030	-0.078	***0.417***	0.003	***0.430***
Runx3	runt-related transcription factor 3	-0.399	0.027	-0.173	***0.747***	-0.057	***0.502***
Cd4	Cd4 molecule	-0.399	0.023	0.235	***0.774***	0.150	***0.859***
Heatr8	HEAT repeat containing 8	-0.399	0.018	0.060	***0.473***	0.000	***0.323***
Nupr1	nuclear protein, transcriptional regulator, 1	-0.399	0.022	-0.043	0.008	-0.013	***0.209***
St3gal1	ST3 beta-galactoside alpha-2,3-sialyltransferase 1	-0.400	0.026	-0.316	0.024	-0.024	***0.601***
Taar7h	trace amine-associated receptor 7h	-0.400	0.029	-0.501	0.011	-0.178	***0.212***
Spdya	speedy homolog A (Xenopus laevis)	-0.400	0.026	-0.056	***0.341***	-0.255	***0.769***
Gspt2	G1 to S phase transition 2	-0.401	0.024	-0.326	0.025	-0.324	0.015
Cabp1	calcium binding protein 1	-0.401	0.027	-0.039	***0.727***	-0.203	***0.940***
Ergic1	endoplasmic reticulum-golgi intermediate compartment (ERGIC) 1	-0.401	0.027	-0.312	0.019	-0.172	***0.810***
Fstl3	follistatin-like 3 (secreted glycoprotein)	-0.401	0.018	-0.054	***0.691***	-0.183	***0.859***
Ncf4	neutrophil cytosolic factor 4	-0.401	0.015	0.238	***0.582***	0.233	***0.599***
Klhl23	kelch-like 23 (Drosophila)	-0.401	0.031	-0.228	***0.929***	-0.092	***0.678***
Cpxm1	carboxypeptidase X (M14 family), member 1	-0.402	0.022	0.252	***0.951***	0.028	***0.636***
Ikbip	IKBKB interacting protein	-0.402	0.021	-0.198	0.039	-0.198	0.036
Gpx4	glutathione peroxidase 4	-0.403	0.025	-0.132	***0.810***	-0.051	***0.707***
Btg3	BTG family, member 3	-0.403	0.029	-0.418	0.009	-0.342	***0.896***
Igf1	insulin-like growth factor 1	-0.403	0.018	0.306	***0.953***	0.070	***0.807***
RGD1565725	similar to hypothetical protein FLJ23834	-0.403	0.037	-0.425	0.015	-0.149	***0.252***
Dlg2	discs, large homolog 2 (Drosophila)	-0.404	0.025	0.036	***0.754***	0.037	***0.784***
Lman1l	lectin, mannose-binding, 1 like	-0.404	0.020	-0.292	***0.979***	-0.062	***0.603***
Tnnt3	troponin T type 3 (skeletal, fast)	-0.404	0.023	0.319	***0.829***	0.066	***0.541***
LOC690977	similar to RIKEN cDNA 5031410I06	-0.404	0.023	0.098	***0.119***	-0.151	***0.435***
Tmigd1	transmembrane and immunoglobulin domain containing 1	-0.404	0.018	-0.339	0.005	0.152	0.015
Tpd52l1	tumor protein D52-like 1	-0.404	0.029	0.102	***0.766***	0.032	***0.647***
Cml3	camello-like 3	-0.405	0.033	0.213	***0.849***	0.299	0.030
Kcne2	potassium voltage-gated channel, Isk-related family, member 2	-0.405	0.015	-0.219	***0.767***	-0.499	0.003
RT1-M1-4	RT1 class I, locus M1, gene 4	-0.405	0.024	-0.324	***0.896***	-0.303	***0.814***
Gpr37l1	G protein-coupled receptor 37-like 1	-0.405	0.027	-0.190	***0.952***	-0.230	***0.975***
Lrrc51	leucine rich repeat containing 51	-0.405	0.016	-0.309	0.021	-0.172	***0.920***
LOC680835	similar to cullin 7	-0.405	0.025	0.105	***0.862***	0.067	***0.671***
Rgs18	regulator of G-protein signaling 18	-0.406	0.029	-0.067	***0.523***	0.108	***0.584***
Tnnc2	troponin C type 2 (fast)	-0.406	0.001	-0.312	0.021	-0.465	0.006
Ddah2	dimethylarginine dimethylaminohydrolase 2	-0.406	0.026	0.055	***0.716***	-0.112	***0.876***
Sp2	Sp2 transcription factor	-0.407	0.031	-0.420	0.006	0.006	***0.152***
Dusp3	dual specificity phosphatase 3	-0.407	0.026	-0.011	***0.542***	-0.159	***0.790***
Pparg	peroxisome proliferator-activated receptor gamma	-0.408	0.027	-0.297	0.009	0.040	***0.199***
Ndufaf4	NADH dehydrogenase (ubiquinone) 1 alpha subcomplex, assembly factor 4	-0.408	0.012	-0.098	***0.763***	-0.105	***0.746***

Cdr2	cerebellar degeneration-related 2	-0.408	0.033	-0.281	***0.847***	-0.572	0.002
Olr594	olfactory receptor 594	-0.409	0.021	-0.158	***0.949***	-0.088	***0.685***
Itgax	integrin, alpha X	-0.410	0.040	-0.146	***0.334***	0.039	***0.391***
Tpm1	tropomyosin 1, alpha	-0.411	0.018	-0.058	***0.808***	-0.133	***0.701***
Rnd3	Rho family GTPase 3	-0.411	0.025	-0.324	0.029	-0.550	0.003
LOC685796	similar to similar to RIKEN cDNA 1700001E04	-0.411	0.027	0.142	***0.633***	0.100	***0.634***
Sema3d	sema domain, immunoglobulin domain (Ig), short basic domain, secreted, (semaphorin) 3D	-0.411	0.022	-0.031	***0.717***	-0.086	***0.829***
P2rx4	purinergic receptor P2X, ligand-gated ion channel 4	-0.411	0.024	-0.266	0.035	-0.248	***0.942***
Aard	alanine and arginine rich domain containing protein	-0.412	0.016	0.129	***0.543***	0.158	***0.756***
Mrgprf	MAS-related GPR, member F	-0.412	0.017	0.078	***0.604***	-0.033	***0.390***
LOC100364244	rCG43589-like	-0.412	0.018	0.061	***0.563***	0.085	***0.447***
Cuzd1	CUB and zona pellucida-like domains 1	-0.412	0.020	-0.203	***0.910***	-0.269	***0.924***
Hspa2	heat shock protein 2	-0.412	0.010	-0.102	***0.773***	-0.004	***0.539***
Aebp1	AE binding protein 1	-0.412	0.033	0.303	0.022	0.045	***0.550***
Lcat	lecithin cholesterol acyltransferase	-0.412	0.017	0.213	***0.776***	-0.099	***0.675***
Rimklb	ribosomal modification protein rimK-like family member B	-0.412	0.019	-0.212	0.037	-0.200	***0.584***
Zc3h12a	zinc finger CCCH type containing 12A	-0.413	0.015	-0.088	***0.656***	-0.305	***0.954***
RGD1565367	similar to Solute carrier family 23, member 2 (Sodium-dependent vitamin C transporter 2)	-0.413	0.030	0.106	***0.590***	0.004	***0.341***
Spock2	sparc/osteonectin, cwcv and kazal-like domains proteoglycan 2	-0.413	0.022	0.060	***0.595***	0.002	***0.430***
Kif6	kinesin family member 6	-0.413	0.031	-0.303	***0.910***	-0.248	***0.870***
Olr35	olfactory receptor 35	-0.413	0.026	-0.563	0.005	0.145	***0.082***
Ocm	oncomodulin	-0.413	0.032	-0.601	0.002	-0.211	0.013
Arf4	ADP-ribosylation factor 4	-0.414	0.026	-0.301	0.037	-0.205	***0.898***
Slc2a4	solute carrier family 2 (facilitated glucose transporter), member 4	-0.415	0.012	-0.032	***0.422***	-0.096	***0.518***
Egf	epidermal growth factor	-0.415	0.025	-0.515	0.003	-0.310	***0.930***
Cytl1	cytokine like 1	-0.415	0.020	-0.018	***0.658***	-0.070	***0.734***
Gys2	glycogen synthase 2	-0.415	0.042	0.468	0.010	0.184	0.016
Zfp692	zinc finger protein 692	-0.416	0.016	0.183	***0.794***	0.258	***0.951***
Art5	ADP-ribosyltransferase 5	-0.416	0.033	-0.449	0.011	-0.392	0.012
Hsd11b1	hydroxysteroid 11-beta dehydrogenase 1	-0.417	0.015	-0.054	***0.811***	-0.080	***0.809***
Pfn2	profilin 2	-0.417	0.025	0.056	***0.557***	-0.125	***0.847***
LOC301748	similar to RIKEN cDNA 1700001E04	-0.417	0.021	0.234	***0.633***	0.229	***0.828***
Pim3	pim-3 oncogene	-0.417	0.018	-0.410	0.013	-0.541	0.002
Folr2	folate receptor 2 (fetal)	-0.417	0.014	0.012	***0.538***	-0.127	***0.819***
Mylk	myosin light chain kinase	-0.418	0.015	-0.142	***0.783***	-0.289	***0.921***
Slitrk6	SLIT and NTRK-like family, member 6	-0.418	0.018	-0.294	***0.980***	-0.344	0.023
Uchl1	ubiquitin carboxyl-terminal esterase L1 (ubiquitin thiolesterase)	-0.418	0.014	0.129	***0.840***	0.042	***0.735***
LOC686921	hypothetical protein LOC686921	-0.419	0.020	0.136	***0.675***	0.087	***0.688***
Prrx1	paired related homeobox 1	-0.419	0.031	-0.122	***0.594***	-0.129	***0.590***
St3gal4	ST3 beta-galactoside alpha-2,3-sialyltransferase 4	-0.419	0.008	-0.076	***0.368***	-0.478	0.003
Scd	stearoyl-CoA desaturase (delta-9-desaturase)	-0.419	0.026	0.058	***0.446***	-0.414	0.011
LOC365985	similar to adenylate kinase 5 isoform 1	-0.419	0.019	0.206	***0.866***	0.063	***0.614***
Pxmp2	peroxisomal membrane protein 2	-0.419	0.021	-0.167	***0.701***	-0.143	***0.644***
Fam3c	family with sequence similarity 3, member C	-0.420	0.021	-0.269	***0.914***	-0.178	***0.853***
Soat1	sterol O-acyltransferase 1	-0.421	0.028	-0.249	***0.757***	-0.145	***0.580***
Fbxo44	F-box protein 44	-0.421	0.023	-0.418	0.009	-0.376	0.018
Col11a1	collagen, type XI, alpha 1	-0.422	0.020	-0.089	***0.750***	-0.122	***0.852***
Cacng6	calcium channel, voltage-dependent, gamma subunit 6	-0.422	0.025	-0.067	***0.643***	0.187	***0.883***
Steap2	STEAP family member 2, metalloreductase	-0.422	0.018	-0.253	***0.781***	-0.358	0.017
Oas3	2 ' -5 ' -oligoadenylate synthetase 3	-0.422	0.025	-0.067	***0.312***	-0.096	***0.356***
Cdh3	cadherin 3	-0.422	0.021	-0.112	***0.773***	-0.055	***0.710***
Fstl3	follistatin-like 3 (secreted glycoprotein)	-0.423	0.019	-0.027	***0.583***	-0.178	***0.850***
Cyp2b15	cytochrome P450, family 2, subfamily b, polypeptide 15	-0.423	0.031	-0.196	***0.653***	-0.100	***0.481***
Sacs	spastic ataxia of Charlevoix-Saguenay (sacsin)	-0.423	0.019	-0.083	***0.764***	-0.073	***0.663***
Art3	ADP-ribosyltransferase 3	-0.424	0.004	0.002	***0.597***	-0.189	***0.892***
Krt17	keratin 17	-0.424	0.031	-0.109	***0.442***	0.221	0.041
Slit2	slit homolog 2 (Drosophila)	-0.424	0.020	0.107	***0.711***	0.046	***0.691***

Bdh2	3-hydroxybutyrate dehydrogenase, type 2	-0.424	0.018	0.027	***0.450***	0.042	***0.459***
Abcb1b	ATP-binding cassette, subfamily B (MDR/TAP), member 1B	-0.424	0.017	-0.020	0.022	0.374	0.007
Casc4	cancer susceptibility candidate 4	-0.424	0.034	-0.302	***0.786***	-0.303	***0.760***
Gucy2g	guanylate cyclase 2G	-0.424	0.022	-0.349	0.015	-0.503	0.003
Ldlr	low density lipoprotein receptor	-0.425	0.022	-0.381	0.018	-0.442	0.010
Armcx6	armadillo repeat containing, X-linked 6	-0.425	0.022	-0.351	0.019	-0.254	***0.865***
Dab2	disabled homolog 2 (Drosophila)	-0.425	0.023	0.105	***0.502***	0.115	***0.730***
Scly	selenocysteine lyase	-0.425	0.015	-0.291	0.037	-0.259	***0.986***
Ppfibp2	PTPRF interacting protein, binding protein 2 (liprin beta 2)	-0.425	0.014	-0.207	***0.884***	-0.175	***0.870***
Hmgcr	3-hydroxy-3-methylglutaryl-CoA reductase	-0.425	0.021	-0.408	0.012	-0.314	0.022
Lilrc2	leukocyte immunoglobulin-like receptor, subfamily C, member 2	-0.425	0.020	-0.036	***0.556***	0.202	***0.874***
Dnajc15	DnaJ (Hsp40) homolog, subfamily C, member 15	-0.426	0.017	-0.287	***0.957***	-0.253	***0.856***
Mamdc2	MAM domain containing 2	-0.426	0.023	-0.123	***0.553***	-0.134	***0.602***
Gnmt	glycine N-methyltransferase	-0.427	0.029	-0.698	0.001	-0.573	0.003
Ccr5	chemokine (C-C motif) receptor 5	-0.428	0.007	-0.203	***0.724***	-0.040	***0.405***
Pcdh8	protocadherin 8	-0.428	0.022	-0.569	0.002	-0.227	***0.450***
Bst2	bone marrow stromal cell antigen 2	-0.428	0.017	0.028	***0.263***	0.171	0.040
Aldh1a1	aldehyde dehydrogenase 1 family, member A1	-0.429	0.015	-0.114	***0.360***	-0.384	0.011
Sel1l3	sel-1 suppressor of lin-12-like 3 (C. elegans)	-0.429	0.023	-0.444	0.006	-0.235	***0.858***
Art3	ADP-ribosyltransferase 3	-0.430	0.011	-0.034	***0.679***	-0.137	***0.767***
Ugp2	UDP-glucose pyrophosphorylase 2	-0.430	0.027	-0.464	0.015	-0.355	0.021
Nr0b2	nuclear receptor subfamily 0, group B, member 2	-0.431	0.003	-0.276	0.035	0.093	***0.728***
Pla2g4a	phospholipase A2, group IVA (cytosolic, calcium-dependent)	-0.431	0.017	-0.219	***0.900***	-0.196	***0.868***
Dio3	deiodinase, iodothyronine, type III	-0.431	0.010	-0.252	***0.594***	0.052	***0.420***
Gpx8	glutathione peroxidase 8	-0.431	0.019	0.059	***0.720***	-0.087	***0.787***
Efcab4a	EF-hand calcium binding domain 4A	-0.431	0.023	-0.409	0.019	-0.046	***0.499***
Foxi1	forkhead box I1	-0.431	0.042	-0.133	***0.203***	0.034	***0.123***
Caly	calcyon neuron-specific vesicular protein	-0.432	0.019	0.121	***0.732***	0.050	***0.704***
Clec4b2	C-type lectin domain family 4, member B2	-0.432	0.017	0.116	***0.579***	0.154	***0.638***
Aspn	asporin	-0.432	0.025	-0.250	***0.853***	-0.396	0.013
Klra1	killer cell lectin-like receptor subfamily A, member 1	-0.432	0.009	0.066	***0.751***	0.152	***0.919***
Myl9	myosin, light chain 9, regulatory	-0.432	0.005	0.093	***0.591***	-0.240	0.026
Hoxa9	homeobox A9	-0.433	0.005	-0.253	***0.884***	0.144	***0.111***
Rell2	RELT-like 2	-0.433	0.016	-0.062	***0.611***	-0.020	***0.537***
Slc35a1	solute carrier family 35 (CMP-sialic acid transporter), member A1	-0.433	0.022	-0.460	0.005	-0.194	***0.745***
LOC680443	hypothetical protein LOC680443	-0.434	0.021	0.013	***0.297***	0.144	***0.783***
Clec10a	C-type lectin domain family 10, member A	-0.434	0.018	0.011	***0.544***	0.084	***0.721***
Kcnj5	potassium inwardly-rectifying channel, subfamily J, member 5	-0.434	0.025	-0.577	0.003	-0.549	0.003
Pter	phosphotriesterase related	-0.434	0.019	-0.251	***0.656***	-0.201	***0.554***
Rbpms	RNA binding protein with multiple splicing	-0.435	0.016	-0.025	***0.623***	-0.151	***0.851***
Pbx3	pre-B-cell leukemia homeobox 3	-0.435	0.024	-0.059	***0.511***	-0.094	***0.487***
Clmp	CXADR-like membrane protein	-0.435	0.023	0.023	***0.557***	-0.021	***0.433***
Hck	hemopoietic cell kinase	-0.436	0.017	0.182	***0.647***	0.207	***0.657***
Gpx3	glutathione peroxidase 3	-0.436	0.006	-0.023	***0.541***	-0.087	***0.400***
LOC680835	similar to cullin 7	-0.436	0.021	0.076	***0.690***	0.046	***0.439***
Itgal	integrin, alpha L	-0.436	0.013	-0.072	***0.216***	0.206	***0.067***
Cacna2d1	calcium channel, voltage-dependent, alpha2/delta subunit 1	-0.437	0.011	0.057	***0.516***	0.139	***0.083***
Lox	lysyl oxidase	-0.437	0.019	0.010	***0.428***	0.317	0.033
Agt	angiotensinogen (serpin peptidase inhibitor, clade A, member 8)	-0.438	0.033	0.084	***0.117***	-0.221	0.029
MGC116197	similar to RIKEN cDNA 1700001E04	-0.438	0.016	0.193	***0.682***	0.175	***0.782***
Adh4	alcohol dehydrogenase 4 (class II), pi polypeptide	-0.438	0.013	-0.458	0.003	-0.049	***0.121***
RGD1308065	hypothetical LOC287935	-0.438	0.010	-0.239	***0.968***	-0.350	***0.994***
Apoa1	apolipoprotein A-I	-0.439	0.007	-1.201	0.000	-0.896	0.000
Bpifa2	BPI fold containing family A, member 2	-0.439	0.022	-0.386	0.020	-0.242	***0.904***
Prelid2	PRELI domain containing 2	-0.439	0.010	-0.509	0.003	-0.193	***0.831***
Kcnk13	potassium channel, subfamily K, member 13	-0.440	0.018	0.118	***0.853***	0.106	***0.717***
Sertad4	SERTA domain containing 4	-0.440	0.020	-0.049	***0.643***	-0.170	***0.891***
LOC302192	similar to RIKEN cDNA 1700001E04	-0.440	0.016	0.167	***0.636***	0.181	***0.584***

Nnat	neuronatin	-0.440	0.032	0.223	***0.781***	0.244	0.027
Gpr114	G protein-coupled receptor 114	-0.440	0.024	0.483	0.009	0.447	0.008
Cbln2	cerebellin 2 precursor	-0.441	0.008	0.055	***0.545***	-0.021	***0.467***
Galr2	galanin receptor 2	-0.441	0.020	-0.003	***0.634***	0.204	***0.880***
Podnl1	podocan-like 1	-0.441	0.022	-0.263	0.029	0.114	***0.051***
Mixl1	Mix paired-like homeobox 1	-0.442	0.012	-0.497	0.004	-0.490	0.004
Alppl2	alkaline phosphatase, placental-like 2	-0.442	0.020	-0.063	***0.539***	-0.122	***0.775***
Flnc	filamin C, gamma	-0.442	0.007	-0.159	***0.289***	-0.505	0.007
Siglec5	sialic acid binding Ig-like lectin 5	-0.442	0.020	0.199	***0.896***	0.108	***0.788***
Sphkap	SPHK1 interactor, AKAP domain containing	-0.443	0.041	0.091	***0.501***	0.110	***0.478***
RT1-CE10	RT1 class I, locus CE10	-0.444	0.007	-0.300	0.005	-0.315	***0.847***
RGD1359108	similar to RIKEN cDNA 3110043O21	-0.444	0.014	-0.137	***0.859***	-0.033	***0.676***
Tsga13	testis specific, 13	-0.444	0.021	-0.314	***0.919***	-0.071	***0.492***
Svs6	seminal vesicle secretory protein 6	-0.444	0.025	-0.248	***0.871***	-0.147	***0.714***
Akap12	A kinase (PRKA) anchor protein 12	-0.445	0.015	0.057	***0.778***	0.059	***0.748***
Hoxa9l	homeobox A9-like	-0.445	0.014	-0.200	***0.842***	0.055	***0.358***
LOC685157	similar to paired immunoglobin-like type 2 receptor beta	-0.445	0.032	0.231	***0.673***	0.295	0.037
Tmx4	thioredoxin-related transmembrane protein 4	-0.446	0.020	-0.179	***0.803***	-0.170	***0.797***
Marveld1	MARVEL domain containing 1	-0.447	0.015	-0.074	***0.758***	-0.107	***0.782***
RGD1359508	similar to protein C33A12.3	-0.447	0.013	-0.168	***0.611***	-0.316	0.022
MGC116197	similar to RIKEN cDNA 1700001E04	-0.448	0.011	0.143	***0.704***	0.136	***0.766***
LOC684055	hypothetical protein LOC684055	-0.448	0.008	-0.781	0.000	-0.518	0.002
T	brachyury homolog (mouse)	-0.448	0.018	-0.161	***0.566***	-0.329	***0.827***
Anp32a	acidic (leucine-rich) nuclear phosphoprotein 32 family, member A	-0.448	0.038	-0.505	0.006	-0.286	***0.613***
RGD1562344	similar to Gm566 protein	-0.448	0.015	-0.262	0.022	-0.213	***0.870***
Krtap21-2	keratin associated protein 21-2	-0.449	0.007	0.052	***0.290***	0.238	0.031
Cfl2	cofilin 2, muscle	-0.449	0.016	-0.227	***0.855***	-0.362	0.012
RGD1560208	similar to Farnesyl pyrophosphate synthetase (FPP synthetase)	-0.449	0.006	-0.312	0.035	-0.141	***0.751***
Slc5a7	solute carrier family 5 (choline transporter), member 7	-0.449	0.013	-0.050	***0.594***	-0.253	***0.937***
LOC681309	similar to Thrombospondin-3 precursor	-0.449	0.010	0.126	***0.850***	0.017	***0.494***
Syngr3	synaptogyrin 3	-0.450	0.011	0.081	***0.700***	0.033	***0.708***
Tbxa2r	thromboxane A2 receptor	-0.450	0.016	-0.155	***0.898***	-0.093	***0.733***
Pax4	paired box 4	-0.450	0.016	-0.076	***0.411***	-0.076	***0.452***
Csrp2	cysteine and glycine-rich protein 2	-0.451	0.015	-0.467	0.005	-0.244	***0.796***
Fdps	farnesyl diphosphate synthase	-0.451	0.009	-0.323	0.035	-0.133	***0.641***
RGD1309085	similar to F23N19.9	-0.452	0.010	-0.327	0.019	-0.009	***0.609***
Fn3k	fructosamine 3 kinase	-0.453	0.025	0.260	0.028	-0.132	***0.338***
Hs3st2	heparan sulfate (glucosamine) 3-O-sulfotransferase 2	-0.453	0.019	-0.017	***0.381***	-0.129	***0.715***
LOC363306	hypothetical protein LOC363306	-0.453	0.012	0.134	***0.741***	0.126	***0.784***
Grhpr	glyoxylate reductase/hydroxypyruvate reductase	-0.454	0.018	-0.239	***0.767***	-0.044	***0.445***
Ppap2b	phosphatidic acid phosphatase type 2B	-0.454	0.033	-0.139	***0.257***	-0.283	0.043
Syne1	spectrin repeat containing, nuclear envelope 1	-0.454	0.013	0.223	***0.883***	-0.119	***0.523***
Ccl2	chemokine (C-C motif) ligand 2	-0.455	0.010	0.042	***0.222***	-0.328	0.023
Stx19	syntaxin 19	-0.455	0.008	-0.519	0.002	-0.080	***0.514***
LOC100125384	hypothetical protein LOC100125384	-0.455	0.019	-0.073	***0.537***	-0.251	***0.761***
Tst	thiosulfate sulfurtransferase	-0.455	0.017	-0.255	0.026	-0.104	***0.271***
C2cd4b	C2 calcium-dependent domain containing 4B	-0.455	0.015	-0.821	0.000	-0.841	0.000
RGD1564704	similar to syntaxin 3A	-0.455	0.015	-0.424	0.012	-0.605	0.001
Apol9a	apolipoprotein L 9a	-0.456	0.015	-0.508	0.006	-0.576	0.003
Spdef	SAM pointed domain containing ets transcription factor	-0.456	0.013	-0.399	0.010	-0.219	***0.911***
Lrat	lecithin-retinol acyltransferase (phosphatidylcholine-retinol-O-acyltransferase)	-0.457	0.025	-0.196	***0.542***	-0.156	***0.436***
Chrd	chordin	-0.457	0.013	-0.242	***0.848***	-0.147	***0.670***
Tnnt2	troponin T type 2 (cardiac)	-0.457	0.006	-0.060	***0.449***	0.067	***0.643***
Lims2	LIM and senescent cell antigen like domains 2	-0.457	0.013	0.100	***0.668***	-0.131	***0.637***
LOC682835	similar to putative pheromone receptor (Go-VN5)	-0.458	0.019	0.064	***0.490***	0.114	***0.668***
LOC682968	similar to Retinal homeobox protein Rx (DRx1) (DRx)	-0.458	0.015	-0.423	0.004	-0.179	***0.549***
Efhd1	EF-hand domain family, member D1	-0.458	0.023	0.129	***0.504***	-0.079	***0.405***
LOC498829	Ab2-143	-0.458	0.014	0.048	***0.523***	0.693	0.000
RGD1564516	similar to smuckler	-0.458	0.040	0.036	0.011	0.042	0.044

Cth	cystathionase (cystathionine gamma-lyase)	-0.459	0.017	-0.327	0.012	0.077	***0.082***
C1qtnf3	C1q and tumor necrosis factor related protein 3	-0.459	0.007	-0.182	***0.871***	-0.084	***0.645***
Csf2ra	granulocyte-macrophage colony stimulating receptor alpha	-0.459	0.021	-0.064	***0.541***	-0.018	***0.505***
Rgc32	response gene to complement 32	-0.460	0.012	-0.442	0.006	-0.316	***0.982***
Ttll9	tubulin tyrosine ligase-like family, member 9	-0.460	0.023	-0.320	0.018	0.014	***0.319***
Wdyhv1	WDYHV motif containing 1	-0.460	0.015	-0.266	***0.892***	-0.368	0.019
Macrod1	MACRO domain containing 1	-0.460	0.014	-0.248	***0.967***	-0.024	***0.500***
Slc26a10	solute carrier family 26, member 10	-0.461	0.013	-0.031	***0.572***	0.103	***0.646***
LOC685904	similar to Spetex-2F protein	-0.461	0.009	0.086	***0.124***	0.248	0.039
LOC691995	hypothetical protein LOC691995	-0.461	0.015	-0.115	***0.855***	-0.042	***0.720***
Fam57b	family with sequence similarity 57, member B	-0.461	0.016	0.045	***0.420***	-0.102	***0.345***
Itgbl1	integrin, beta-like 1	-0.461	0.007	-0.089	***0.793***	-0.128	***0.798***
Actn2	actinin alpha 2	-0.461	0.015	0.038	***0.564***	-0.212	***0.931***
Hoxa2	homeo box A2	-0.462	0.021	-0.243	***0.720***	-0.159	***0.578***
Penk	proenkephalin	-0.463	0.012	0.040	***0.684***	-0.146	***0.820***
Crem	cAMP responsive element modulator	-0.464	0.015	-0.313	0.032	-0.566	0.001
Ces2j	carboxylesterase 2J	-0.464	0.011	-0.210	***0.755***	-0.046	***0.477***
C1qtnf6	C1q and tumor necrosis factor related protein 6	-0.464	0.013	-0.343	0.026	-0.201	***0.897***
Snap25	synaptosomal-associated protein 25	-0.465	0.012	-0.036	***0.346***	-0.069	***0.433***
Zfp9	zinc finger protein 9	-0.465	0.014	-0.407	0.009	-0.522	0.003
Mrpl18	mitochondrial ribosomal protein L18	-0.466	0.014	-0.318	0.023	-0.570	0.001
RGD1309808	similar to apolipoprotein L2; apolipoprotein L-II	-0.466	0.013	-0.396	0.016	-0.188	***0.769***
Klhdc8a	kelch domain containing 8A	-0.467	0.008	-0.493	0.004	-0.209	0.032
Cfl2	cofilin 2, muscle	-0.467	0.008	-0.235	***0.673***	-0.355	0.015
Chst8	carbohydrate (N-acetylgalactosamine 4-0) sulfotransferase 8	-0.467	0.013	-0.234	***0.910***	-0.363	0.023
Styx	serine/threonine/tyrosine interacting protein	-0.468	0.011	-0.315	***0.942***	-0.105	***0.582***
Tpm2	tropomyosin 2, beta	-0.468	0.009	-0.156	***0.850***	-0.326	***0.954***
Nexn	nexilin (F actin binding protein)	-0.468	0.010	-0.018	***0.501***	-0.205	***0.751***
Ppt1	palmitoyl-protein thioesterase 1	-0.469	0.015	0.125	***0.639***	0.125	***0.871***
Crabp1	cellular retinoic acid binding protein 1	-0.469	0.005	-0.008	***0.237***	0.060	***0.484***
Cst3	cystatin C	-0.469	0.014	0.002	***0.541***	0.037	***0.719***
Slc38a5	solute carrier family 38, member 5	-0.470	0.006	0.168	***0.899***	0.062	***0.469***
Slc6a7	solute carrier family 6 (neurotransmitter transporter, L-proline), member 7	-0.470	0.013	-0.173	***0.540***	-0.289	***0.719***
Tac1	tachykinin 1	-0.470	0.008	-0.069	***0.445***	0.000	***0.417***
Tifab	TRAF-interacting protein with forkhead-associated domain, family member B	-0.470	0.016	0.187	***0.455***	0.169	***0.782***
Fzd2	frizzled family receptor 2	-0.470	0.008	-0.178	***0.893***	-0.346	0.023
Trpc4	transient receptor potential cation channel, subfamily C, member 4	-0.471	0.005	-0.243	***0.318***	-0.450	0.018
Itga7	integrin, alpha 7	-0.471	0.007	-0.125	***0.611***	-0.260	***0.839***
Shisa3	shisa homolog 3 (Xenopus laevis)	-0.472	0.009	0.030	***0.399***	-0.048	***0.526***
Maob	monoamine oxidase B	-0.472	0.010	-0.020	***0.589***	0.023	***0.603***
Lgi4	leucine-rich repeat LGI family, member 4	-0.472	0.014	0.058	***0.645***	0.027	***0.576***
Rarres1	retinoic acid receptor responder (tazarotene induced) 1	-0.472	0.003	-0.249	***0.360***	0.107	***0.120***
Cdc42ep3	CDC42 effector protein (Rho GTPase binding) 3	-0.472	0.012	-0.420	0.013	-0.487	0.004
Derl3	Der1-like domain family, member 3	-0.473	0.023	0.181	***0.654***	0.287	0.017
Copz2	coatomer protein complex, subunit zeta 2	-0.473	0.014	-0.321	0.024	-0.320	***0.889***
Mrgprx3	MAS-related GPR, member X3	-0.473	0.027	0.165	***0.514***	0.402	0.033
Map1b	microtubule-associated protein 1B	-0.474	0.015	-0.305	0.037	-0.209	***0.667***
Sync	syncoilin	-0.474	0.016	-0.179	***0.773***	-0.177	***0.826***
Opn4	opsin 4	-0.474	0.024	-0.333	0.035	-0.270	***0.674***
LOC686318	similar to NIMA (never in mitosis gene a)-related expressed kinase 5	-0.475	0.007	-0.463	0.005	-0.090	***0.528***
LOC100366216	nuclear antigen Sp100-like	-0.475	0.014	-0.046	***0.481***	0.156	***0.691***
LOC686323	similar to thyroid receptor-interacting protein 6	-0.475	0.012	-0.112	***0.889***	-0.231	***0.974***
Nrn1l	neuritin 1-like	-0.476	0.017	-0.231	***0.905***	-0.144	***0.745***
Fdps	farnesyl diphosphate synthase	-0.476	0.009	-0.342	0.028	-0.127	***0.613***
Bok	BCL2-related ovarian killer	-0.477	0.014	-0.375	0.021	-0.348	0.027
Ccdc163	coiled-coil domain containing 163	-0.477	0.012	-0.190	***0.922***	-0.127	***0.821***
C6	complement component 6	-0.477	0.027	-0.112	0.045	-0.292	***0.548***
Prima1	proline rich membrane anchor 1	-0.477	0.014	-0.166	***0.534***	-0.124	***0.419***
Pkhd1l1	polycystic kidney and hepatic disease 1-like 1	-0.478	0.018	0.099	***0.418***	0.038	***0.432***

Isg15	ISG15 ubiquitin-like modifier	-0.478	0.011	-0.263	***0.720***	0.097	***0.476***
Fbp1	fructose-1,6-bisphosphatase 1	-0.479	0.009	-0.201	***0.936***	-0.079	***0.585***
Fes	feline sarcoma oncogene	-0.479	0.014	-0.103	***0.767***	0.033	***0.681***
Slc24a3	solute carrier family 24 (sodium/potassium/calcium exchanger), member 3	-0.479	0.013	-0.012	***0.385***	-0.105	***0.489***
Bag2	Bcl2-associated athanogene 2	-0.480	0.009	-0.285	***0.789***	-0.475	0.007
Slc35c1	solute carrier family 35, member C1	-0.480	0.012	-0.277	0.016	-0.263	***0.966***
Ng23	Ng23 protein	-0.480	0.011	-0.013	***0.726***	-0.019	***0.597***
LOC100366259	rCG52099-like	-0.480	0.011	-0.094	***0.706***	-0.165	***0.907***
LOC501089	similar to Discs large homolog 5 (Placenta and prostate DLG) (Discs large protein P-dlg)	-0.481	0.008	0.031	***0.402***	0.054	***0.397***
Aox1	aldehyde oxidase 1	-0.481	0.010	0.225	***0.946***	0.041	***0.584***
LOC501222	similar to Discs large homolog 5 (Placenta and prostate DLG) (Discs large protein P-dlg)	-0.482	0.008	0.023	***0.368***	0.021	***0.492***
Amt	aminomethyltransferase	-0.483	0.011	-0.438	0.008	-0.225	***0.639***
Fgg	fibrinogen gamma chain	-0.483	0.018	0.027	***0.166***	0.180	***0.774***
Cav3	caveolin 3	-0.483	0.010	-0.215	***0.570***	-0.161	0.049
Fkbp7	FK506 binding protein 7	-0.483	0.012	-0.033	***0.724***	-0.019	***0.558***
Creb3l4	cAMP responsive element binding protein 3-like 4	-0.484	0.012	-0.262	***0.949***	-0.119	***0.751***
Grp	gastrin releasing peptide	-0.484	0.001	0.071	***0.513***	0.049	***0.563***
Sat2	spermidine/spermine N1-acetyltransferase family member 2	-0.484	0.012	0.018	***0.562***	-0.088	***0.804***
P4htm	prolyl 4-hydroxylase, transmembrane	-0.485	0.008	-0.141	***0.895***	-0.211	***0.955***
LOC689826	hypothetical protein LOC689826	-0.485	0.006	-0.087	***0.471***	-0.130	***0.511***
Zfat	zinc finger and AT hook domain containing	-0.485	0.011	-0.214	***0.946***	-0.149	***0.833***
Des	desmin	-0.485	0.007	-0.215	***0.896***	-0.277	***0.826***
Mpz	myelin protein zero	-0.485	0.017	0.351	0.021	0.500	0.006
Ms4a4a	membrane-spanning 4-domains, subfamily A, member 4A	-0.486	0.005	0.097	***0.488***	0.060	***0.414***
Epha7	Eph receptor A7	-0.486	0.019	-0.111	***0.204***	0.028	***0.066***
Hfe	hemochromatosis	-0.486	0.013	-0.252	***0.775***	-0.176	***0.629***
Pxmp2	peroxisomal membrane protein 2	-0.487	0.009	-0.230	***0.952***	-0.276	***0.965***
Kcnmb1	potassium large conductance calcium-activated channel, subfamily M, beta member 1	-0.487	0.008	-0.081	***0.449***	-0.164	***0.586***
Adamdec1	ADAM-like, decysin 1	-0.488	0.004	0.131	***0.855***	0.207	***0.839***
Car9	carbonic anhydrase 9	-0.489	0.010	-0.560	0.001	-0.167	***0.748***
Schip1	schwannomin interacting protein 1	-0.489	0.010	-0.195	***0.942***	-0.322	***0.992***
Sez6	seizure related 6 homolog (mouse)	-0.490	0.011	0.022	***0.691***	-0.072	***0.822***
Noxo1	NADPH oxidase organizer 1	-0.491	0.005	-0.201	***0.830***	-0.081	***0.651***
Kctd14	potassium channel tetramerisation domain containing 14	-0.491	0.012	-0.374	0.012	-0.278	***0.712***
Fa2h	fatty acid 2-hydroxylase	-0.491	0.005	-0.580	0.000	-0.133	0.017
Enc1	ectodermal-neural cortex 1	-0.491	0.009	-0.737	0.000	-0.783	0.000
Lect1	leukocyte cell derived chemotaxin 1	-0.491	0.010	-0.294	***0.949***	-0.352	0.025
Hspb1	heat shock protein 1	-0.492	0.005	-0.023	***0.467***	-0.230	***0.873***
Slc25a25	solute carrier family 25 (mitochondrial carrier, phosphate carrier), member 25	-0.492	0.010	-0.332	0.031	-0.617	0.001
Slc35f2	solute carrier family 35, member F2	-0.492	0.012	-0.053	***0.593***	-0.145	***0.837***
Zbp1	Z-DNA binding protein 1	-0.492	0.006	0.020	***0.396***	0.242	***0.804***
C1qc	complement component 1, q subcomponent, C chain	-0.492	0.009	0.162	***0.808***	0.132	***0.791***
Ptprv	protein tyrosine phosphatase, receptor type, V	-0.492	0.007	-0.285	***0.951***	-0.023	***0.581***
Nsg2	neuron specific gene family member 2	-0.493	0.014	-0.287	0.038	-0.227	***0.674***
Slc4a4	solute carrier family 4, sodium bicarbonate cotransporter, member 4	-0.493	0.008	-0.614	0.001	-0.062	0.018
Ppp1cb	protein phosphatase 1, catalytic subunit, beta isozyme	-0.493	0.011	-0.473	0.004	-0.202	***0.529***
Ptpla	protein tyrosine phosphatase-like (proline instead of catalytic arginine), member a	-0.493	0.009	-0.083	***0.602***	-0.231	***0.778***
Vsig2	V-set and immunoglobulin domain containing 2	-0.493	0.008	-0.593	0.001	0.075	***0.853***
LOC688090	similar to RT1 class II histocompatibility antigen, B-1 beta chain precursor (RT1.B-beta(1))	-0.493	0.026	0.285	0.030	0.361	0.018
Sstr2	somatostatin receptor 2	-0.493	0.006	-0.823	0.000	-0.547	0.002
Padi1	peptidyl arginine deiminase, type I	-0.494	0.007	-0.313	0.037	-0.146	***0.824***
Cd68	Cd68 molecule	-0.495	0.012	0.169	***0.817***	0.228	***0.902***
Fmod	fibromodulin	-0.495	0.006	0.337	0.028	0.165	***0.767***

LOC683626	similar to limb-bud and heart	-0.496	0.010	-0.451	0.009	-0.136	***0.587***
Angptl1	angiopoietin-like 1	-0.496	0.017	-0.247	***0.710***	-0.556	0.002
Cpz	carboxypeptidase Z	-0.497	0.010	0.445	0.010	0.305	0.033
Fas	Fas (TNF receptor superfamily, member 6)	-0.497	0.008	-0.290	***0.989***	-0.482	0.004
Adh7	alcohol dehydrogenase 7 (class IV), mu or sigma polypeptide	-0.497	0.009	-0.449	0.004	-0.034	***0.255***
Sacm1l	SAC1 suppressor of actin mutations 1-like (yeast)	-0.498	0.012	-0.294	***0.812***	-0.273	***0.727***
Gpr55	G protein-coupled receptor 55	-0.498	0.016	0.031	***0.479***	0.001	***0.407***
Fam64a	family with sequence similarity 64, member A	-0.499	0.009	-0.556	0.002	-0.247	0.044
LOC680236	hypothetical protein LOC680236	-0.499	0.010	0.138	***0.492***	0.105	***0.734***
LOC683751	similar to trophinin isoform 1	-0.499	0.015	-0.019	***0.067***	0.315	0.023
Tgfb2	transforming growth factor, beta 2	-0.499	0.023	-0.305	0.047	-0.531	0.018
Slc4a3	solute carrier family 4 (anion exchanger), member 3	-0.499	0.009	0.230	***0.982***	-0.015	***0.500***
LOC685438	similar to paired immunoglobin-like type 2 receptor beta	-0.500	0.030	0.179	***0.336***	0.338	0.010
Olr664	olfactory receptor 664	-0.500	0.012	-0.024	***0.289***	0.317	0.028
Tagln	transgelin	-0.501	0.007	-0.107	***0.751***	-0.331	0.022
Cd44	Cd44 molecule	-0.501	0.009	-0.649	0.001	-0.357	0.019
RGD1310039	similar to hypothetical protein FLJ10058	-0.501	0.026	-0.498	0.018	-0.079	***0.388***
Dtnb	dystrobrevin, beta	-0.501	0.009	-0.168	***0.853***	0.020	***0.586***
RGD1561472	similar to mKIAA2005 protein	-0.502	0.012	0.034	***0.286***	0.090	***0.354***
Cmpk2	cytidine monophosphate (UMP-CMP) kinase 2, mitochondrial	-0.502	0.011	-0.236	***0.780***	-0.143	***0.688***
Ppt1	palmitoyl-protein thioesterase 1	-0.502	0.009	0.121	***0.633***	0.199	***0.930***
C1qa	complement component 1, q subcomponent, A chain	-0.502	0.014	0.169	***0.757***	0.095	***0.686***
Fxyd1	FXYD domain-containing ion transport regulator 1	-0.502	0.010	0.015	***0.617***	0.035	***0.658***
LOC691485	hypothetical protein LOC691485	-0.503	0.014	-0.171	***0.457***	-0.267	***0.790***
C1qtnf2	C1q and tumor necrosis factor related protein 2	-0.503	0.010	0.028	***0.674***	-0.088	***0.789***
Ap1s2	adaptor-related protein complex 1, sigma 2 subunit	-0.504	0.010	0.064	***0.620***	0.172	***0.874***
Fam176a	family with sequence similarity 176, member A	-0.505	0.009	-0.283	***0.975***	-0.298	***0.992***
LOC681383	similar to Protein C10orf11 homolog	-0.505	0.013	-0.019	***0.393***	0.016	***0.481***
Cpsf4l	cleavage and polyadenylation specific factor 4-like	-0.505	0.027	-0.515	0.011	-0.032	***0.143***
Lrrc4	leucine rich repeat containing 4	-0.505	0.027	-0.247	***0.458***	-0.345	***0.701***
Lrrn3	leucine rich repeat neuronal 3	-0.505	0.018	-0.374	0.034	-0.058	***0.399***
Nkain4	Na+/K+ transporting ATPase interacting 4	-0.505	0.030	0.047	***0.408***	-0.009	***0.119***
PVR	poliovirus receptor	-0.506	0.006	-0.419	0.011	-0.425	0.009
Car13	carbonic anhydrase 13	-0.506	0.011	-0.574	0.001	-0.296	0.018
Kazald1	Kazal-type serine peptidase inhibitor domain 1	-0.506	0.008	0.018	***0.671***	0.111	***0.881***
Lgals3bp	lectin, galactoside-binding, soluble, 3 binding protein	-0.507	0.008	0.145	***0.832***	0.128	***0.808***
Efemp2	EGF-containing fibulin-like extracellular matrix protein 2	-0.508	0.010	0.110	***0.831***	-0.056	***0.618***
Myoz1	myozenin 1	-0.509	0.006	-0.046	***0.595***	-0.175	***0.866***
Rtp4	receptor (chemosensory) transporter protein 4	-0.510	0.007	-0.305	***0.939***	-0.235	***0.751***
Pltp	phospholipid transfer protein	-0.510	0.003	0.145	***0.882***	0.207	***0.914***
RGD1560792	RGD1560792	-0.511	***0.058***	0.240	0.009	0.059	0.023
Serpine2	serine (or cysteine) peptidase inhibitor, clade E, member 2	-0.511	0.008	-0.129	***0.712***	-0.112	***0.640***
Ces1d	carboxylesterase 1D	-0.511	0.000	-0.278	0.025	-1.045	0.000
Hif1a	hypoxia-inducible factor 1, alpha subunit (basic helix-loop-helix transcription factor)	-0.511	0.008	-0.274	0.029	-0.340	0.015
Slco6b1	solute carrier organic anion transporter family, member 6b1	-0.511	0.022	-0.409	0.026	-0.177	***0.489***
Ambp	alpha-1-microglobulin/bikunin precursor	-0.512	0.006	-0.087	***0.617***	-0.111	***0.577***
LOC688582	similar to hemicentin 1	-0.513	0.010	0.026	***0.319***	-0.127	***0.462***
Mptx	mucosal pentraxin	-0.514	0.001	1.054	0.000	1.409	0.000
Aldh1l2	aldehyde dehydrogenase 1 family, member L2	-0.514	0.008	-0.281	0.027	-0.206	***0.579***
Popdc2	popeye domain containing 2	-0.514	0.005	-0.057	***0.560***	-0.310	***0.986***
P2ry12	purinergic receptor P2Y, G-protein coupled, 12	-0.515	0.012	0.131	***0.644***	0.363	0.013
Cd55	Cd55 molecule	-0.515	0.011	-0.214	0.044	0.258	0.008
Pi15	peptidase inhibitor 15	-0.515	0.012	0.208	***0.363***	0.012	***0.363***
Nr4a3	nuclear receptor subfamily 4, group A, member 3	-0.515	0.040	-0.201	***0.327***	-0.463	***0.875***

Slc39a4	solute carrier family 39 (zinc transporter), member 4	-0.515	0.007	-0.461	0.006	0.061	***0.616***
Exnef	exonuclease NEF-sp	-0.515	0.005	-0.342	0.033	0.156	***0.763***
RGD1561916	similar to testes development-related NYD-SP22 isoform 1	-0.515	0.009	-0.449	0.008	-0.514	0.003
Gpc3	glypican 3	-0.517	0.007	0.142	***0.745***	-0.054	***0.496***
Azgp1	alpha-2-glycoprotein 1, zinc-binding	-0.517	0.003	-0.330	0.001	-1.262	0.000
Dmbt1	deleted in malignant brain tumors 1	-0.518	0.001	-0.781	0.000	0.208	0.014
Mllt11	myeloid/lymphoid or mixed-lineage leukemia (trithorax homolog, Drosophila); translocated to, 11	-0.518	0.007	-0.242	***0.843***	-0.363	***0.971***
Baiap2l2	BAI1-associated protein 2-like 2	-0.520	0.008	-0.221	***0.819***	0.013	***0.314***
Olr1364	olfactory receptor 1364	-0.520	0.013	0.364	0.006	0.128	0.035
Dcn	decorin	-0.522	0.003	0.202	***0.811***	-0.035	***0.326***
RGD1311080	similar to RIKEN cDNA A930038C07	-0.522	0.016	-0.100	***0.298***	-0.105	***0.284***
Slc7a10	solute carrier family 7 (neutral amino acid transporter light chain, asc system), member 10	-0.522	0.014	0.095	***0.058***	-0.130	***0.054***
C4bpb	complement component 4 binding protein, beta	-0.522	0.009	-0.323	0.023	0.256	***0.129***
Klk8	kallikrein related-peptidase 8	-0.523	0.008	-0.214	0.031	0.058	***0.072***
Nkd2	naked cuticle homolog 2 (Drosophila)	-0.523	0.009	-0.061	***0.652***	-0.237	***0.935***
Dusp27	dual specificity phosphatase 27 (putative)	-0.524	0.008	-0.650	0.001	-0.559	0.002
Krtap16-5	keratin associated protein 16-5	-0.524	0.002	-0.043	***0.290***	0.216	***0.051***
RGD1559710	similar to DC-SIGN	-0.524	0.008	-0.055	***0.506***	-0.082	***0.602***
Ntrk3	neurotrophic tyrosine kinase, receptor, type 3	-0.525	0.008	0.198	***0.853***	0.011	***0.276***
Xirp1	xin actin-binding repeat containing 1	-0.525	0.041	-0.663	0.004	-0.232	0.021
Mid1ip1	MID1 interacting protein 1 (gastrulation specific G12 homolog (zebrafish))	-0.525	0.009	-0.439	0.010	-0.454	0.006
P2ry13	purinergic receptor P2Y, G-protein coupled, 13	-0.526	0.008	0.007	***0.560***	0.046	***0.500***
Tnnt2	troponin T type 2 (cardiac)	-0.526	0.003	0.034	***0.484***	0.141	***0.807***
Best1	bestrophin 1	-0.527	0.028	-0.637	0.004	-0.121	***0.205***
Rarres2	retinoic acid receptor responder (tazarotene induced) 2	-0.528	0.005	0.145	***0.838***	0.086	***0.418***
Mx1	myxovirus (influenza virus) resistance 1	-0.528	0.005	-0.004	***0.367***	0.242	***0.812***
Lypd6b	LY6/PLAUR domain containing 6B	-0.529	0.036	-0.444	0.036	-0.205	***0.323***
LOC499806	similar to RIKEN cDNA 4933404M02	-0.530	0.034	-0.299	***0.062***	-0.088	0.045
Nxph1	neurexophilin 1	-0.531	0.014	-0.352	0.021	-0.311	***0.831***
Coq10b	coenzyme Q10 homolog B (S. cerevisiae)	-0.532	0.006	-0.282	***0.911***	-0.373	0.022
Adam28	ADAM metallopeptidase domain 28	-0.533	0.012	-0.305	***0.719***	-0.186	***0.463***
Calcb	calcitonin-related polypeptide, beta	-0.533	0.005	-0.037	***0.436***	-0.056	***0.513***
Tgm2	transglutaminase 2, C polypeptide	-0.533	0.007	0.154	***0.816***	0.110	***0.678***
Fxyd2	FXYD domain-containing ion transport regulator 2	-0.533	0.007	0.071	***0.758***	0.018	***0.610***
Ccna1	cyclin A1	-0.534	0.005	-0.509	0.002	-0.415	0.007
Defb25	defensin beta 25	-0.534	0.008	-0.306	***0.962***	-0.268	***0.932***
Ramp1	receptor (G protein-coupled) activity modifying protein 1	-0.535	0.008	-0.448	0.005	-0.201	***0.782***
Pklr	pyruvate kinase, liver and RBC	-0.536	0.005	-0.425	0.005	-0.137	***0.438***
Lrrc36	leucine rich repeat containing 36	-0.538	0.007	-0.356	0.029	-0.161	***0.574***
LOC362347	similar to protease, serine, 3 (mesotrypsin)	-0.538	0.005	0.012	0.000	-0.148	0.004
Fam176a	family with sequence similarity 176, member A	-0.538	0.007	-0.331	0.031	-0.229	***0.929***
Slc35b3	solute carrier family 35, member B3	-0.538	0.007	-0.250	0.019	-0.105	***0.347***
Plp1	proteolipid protein 1	-0.540	0.007	-0.134	***0.813***	-0.310	***0.985***
Eya2	eyes absent homolog 2 (Drosophila)	-0.541	0.007	-0.268	***0.903***	-0.025	***0.424***
Rhoq	ras homolog gene family, member Q	-0.541	0.008	-0.299	0.035	-0.358	0.020
Egf	epidermal growth factor	-0.542	0.008	-0.562	0.002	-0.338	0.024
RGD1565161	similar to dendritic cell-derived immunoglobulin(Ig)-like receptor 1, DIgR1 - mouse	-0.542	0.006	0.080	***0.682***	-0.055	***0.491***
Trpc3	transient receptor potential cation channel, subfamily C, member 3	-0.544	0.005	-0.078	***0.808***	-0.149	***0.914***
Fbxl22	F-box and leucine-rich repeat protein 22	-0.544	0.003	-0.126	***0.713***	-0.092	***0.629***
Has1	hyaluronan synthase 1	-0.544	0.004	-0.306	0.025	-0.669	0.000
RGD1564419	similar to hypothetical gene supported by BC025338	-0.545	0.004	-0.174	***0.818***	-0.104	***0.774***
Filip1	filamin A interacting protein 1	-0.545	0.008	-0.047	***0.388***	-0.458	0.010
S100b	S100 calcium binding protein B	-0.546	0.016	0.165	***0.646***	-0.041	***0.323***
Gdf10	growth differentiation factor 10	-0.546	0.007	0.080	***0.618***	0.125	***0.724***
Gria4	glutamate receptor, ionotrophic, AMPA 4	-0.546	0.008	-0.142	***0.614***	-0.094	***0.618***
Plekhb1	pleckstrin homology domain containing, family B (evectins) member 1	-0.547	0.007	0.138	***0.673***	-0.051	***0.615***

Pkhd1l1	polycystic kidney and hepatic disease 1-like 1	-0.547	0.005	-0.063	***0.750***	-0.187	***0.861***
Tspan2	tetraspanin 2	-0.549	0.007	-0.092	***0.596***	-0.185	***0.774***
Adrb3	adrenergic, beta-3-, receptor	-0.549	0.005	0.062	***0.718***	-0.257	***0.905***
C1qb	complement component 1, q subcomponent, B chain	-0.549	0.007	0.170	***0.812***	0.121	***0.803***
Nts	neurotensin	-0.551	0.024	-0.603	0.007	-0.171	***0.333***
Fam5b	family with sequence similarity 5, member B	-0.552	0.006	-0.266	***0.945***	-0.448	0.007
RGD1561574	similar to Hypothetical protein MGC76322	-0.552	0.007	-0.539	0.003	-0.267	0.004
Stc1	stanniocalcin 1	-0.552	0.009	-0.308	0.030	-0.010	***0.352***
Hspb8	heat shock protein B8	-0.554	0.004	-0.013	***0.503***	-0.268	***0.960***
Fst	follistatin	-0.555	0.005	0.445	0.003	0.613	0.000
Mst1	Macrophage stimulating 1 (hepatocyte growth factor-like)	-0.555	0.012	0.071	***0.087***	-0.279	***0.557***
Cst6	cystatin E/M	-0.555	0.006	-0.500	0.003	-0.062	***0.384***
Ldhb	lactate dehydrogenase B	-0.556	0.005	-0.031	***0.726***	0.058	***0.811***
Slc39a8	solute carrier family 39 (zinc transporter), member 8	-0.557	0.007	-0.437	0.008	-0.220	***0.753***
Nppc	natriuretic peptide C	-0.558	0.004	-0.291	***0.972***	-0.229	***0.972***
Spock2	sparc/osteonectin, cwcv and kazal-like domains proteoglycan 2	-0.558	0.005	0.144	***0.733***	0.173	***0.694***
LOC301193	similar to Discs large homolog 5 (Placenta and prostate DLG) (Discs large protein P-dlg)	-0.559	0.007	0.174	***0.545***	0.167	***0.843***
Tacr2	tachykinin receptor 2	-0.559	0.004	0.122	***0.360***	-0.024	***0.285***
Vtn	vitronectin	-0.559	0.005	-0.046	***0.659***	-0.179	***0.913***
Car13	carbonic anhydrase 13	-0.560	0.006	-0.422	0.012	-0.277	***0.808***
LOC500118	similar to RIKEN cDNA D330028D13	-0.560	0.007	-0.385	0.021	-0.406	0.015
Prelid2	PRELI domain containing 2	-0.561	0.005	-0.391	0.007	-0.157	***0.527***
Elmod1	ELMO/CED-12 domain containing 1	-0.561	0.007	0.007	***0.224***	-0.116	***0.430***
Vegfb	vascular endothelial growth factor B	-0.562	0.005	0.091	***0.750***	-0.036	***0.567***
Lgals1	lectin, galactoside-binding, soluble, 1	-0.562	0.005	0.065	***0.778***	-0.074	***0.650***
Ltc4s	leukotriene C4 synthase	-0.562	0.003	-0.140	***0.897***	-0.205	***0.957***
Cd44	Cd44 molecule	-0.563	0.004	-0.565	0.002	-0.495	0.003
Fam132a	family with sequence similarity 132, member A	-0.563	0.005	-0.012	***0.506***	-0.196	***0.866***
Ccdc80	coiled-coil domain containing 80	-0.563	0.004	0.389	0.017	0.004	***0.490***
RGD1561530	similar to Tle6 protein	-0.563	0.005	-0.399	0.009	-0.356	0.022
Cd99l2	CD99 molecule-like 2	-0.564	0.005	-0.652	0.000	-0.210	***0.779***
Mlxipl	MLX interacting protein-like	-0.564	0.007	-0.038	***0.053***	0.229	0.027
Kcnh2	potassium voltage-gated channel, subfamily H (eag-related), member 2	-0.564	0.007	-0.062	***0.501***	-0.114	***0.510***
Tbxas1	thromboxane A synthase 1, platelet	-0.565	0.005	-0.426	0.008	-0.327	***0.981***
Spta1	spectrin, alpha, erythrocytic 1 (elliptocytosis 2)	-0.565	0.038	-0.232	***0.485***	-0.048	***0.156***
LOC100364551	rCG52842-like	-0.566	0.006	-0.460	0.005	-0.331	0.027
Fndc1	fibronectin type III domain containing 1	-0.567	0.003	0.083	***0.457***	-0.001	***0.338***
Scd4	stearoyl-coenzyme A desaturase 4	-0.567	0.025	0.037	***0.057***	-0.211	0.040
Ccl7	chemokine (C-C motif) ligand 7	-0.568	0.006	0.008	***0.325***	-0.243	***0.681***
P2rx2	purinergic receptor P2X, ligand-gated ion channel, 2	-0.569	0.006	-0.286	***0.881***	-0.268	***0.833***
Ntm	neurotrimin	-0.570	0.007	-0.358	0.027	-0.330	0.025
Itih4	inter alpha-trypsin inhibitor, heavy chain 4	-0.571	0.004	-0.107	***0.609***	0.017	***0.385***
Prkg2	protein kinase, cGMP-dependent, type II	-0.573	0.005	-0.427	0.000	0.166	0.037
Pdk3	pyruvate dehydrogenase kinase, isozyme 3	-0.573	0.005	-0.215	0.021	-0.201	***0.568***
Ttll7	tubulin tyrosine ligase-like family, member 7	-0.573	0.004	-0.101	***0.731***	-0.229	***0.949***
Gatm	glycine amidinotransferase (L-arginine:glycine amidinotransferase)	-0.574	0.005	-0.128	***0.786***	-0.049	***0.655***
Sec11c	SEC11 homolog C (S. cerevisiae)	-0.574	0.004	-0.251	***0.943***	-0.119	***0.741***
Pcsk4	proprotein convertase subtilisin/kexin type 4	-0.574	0.006	-0.435	0.010	-0.297	***0.881***
RGD1562667	similar to leukocyte mono-Ig-like receptor2	-0.575	0.004	-0.061	***0.577***	0.037	***0.476***
Pla2g4f	phospholipase A2, group IVF	-0.577	***0.061***	-0.541	0.024	0.023	***0.087***
RT1-Bb	RT1 class II, locus Bb	-0.577	0.014	0.295	0.024	0.423	0.014
Ube2q2l	ubiquitin-conjugating enzyme E2Q family member 2-like	-0.577	0.006	-0.601	0.001	-0.380	0.012
Adam1a	a disintegrin and metallopeptidase domain 1a	-0.578	0.002	-0.139	***0.838***	-0.425	0.006
Cnn1	calponin 1, basic, smooth muscle	-0.578	0.000	-0.132	***0.674***	-0.317	0.010
Usp18	ubiquitin specific peptidase 18	-0.578	0.004	-0.140	***0.580***	0.037	***0.344***
Ppic	peptidylprolyl isomerase C	-0.578	0.004	-0.191	***0.775***	-0.202	***0.793***
Scarf2	scavenger receptor class F, member 2	-0.578	0.005	-0.029	***0.666***	-0.093	***0.603***
Tspan11	tetraspanin 11	-0.578	0.003	0.067	***0.717***	-0.013	***0.567***

Bend5	BEN domain containing 5	-0.579	0.005	-0.113	***0.523***	0.086	***0.558***
Muc2	mucin 2, oligomeric mucus/gel-forming	-0.579	0.003	-0.563	0.001	0.210	0.029
Ly6h	lymphocyte antigen 6 complex, locus H	-0.579	0.007	0.080	***0.585***	-0.082	***0.628***
Nell2	NEL-like 2 (chicken)	-0.579	0.005	-0.149	***0.605***	-0.126	***0.556***
Zkscan2	zinc finger with KRAB and SCAN domains 2	-0.580	0.009	-0.455	0.008	-0.115	***0.152***
Dusp1	dual specificity phosphatase 1	-0.581	0.002	-0.209	***0.721***	-0.476	0.004
Tpm2	tropomyosin 2, beta	-0.581	0.004	0.137	***0.653***	-0.147	***0.838***
Itih3	inter-alpha trypsin inhibitor, heavy chain 3	-0.581	0.003	0.049	***0.411***	-0.051	***0.618***
Srcrb4d	scavenger receptor cysteine rich domain containing, group B (4 domains)	-0.582	0.003	-0.093	***0.265***	-0.300	0.006
Acad10	acyl-CoA dehydrogenase family, member 10	-0.584	0.003	-0.216	***0.779***	-0.385	0.013
LOC679119	similar to bone morphogenetic protein 8b	-0.584	0.006	-0.410	0.001	-0.480	0.005
Mest	mesoderm specific transcript homolog (mouse)	-0.584	0.009	0.390	0.021	0.220	***0.053***
Casq1	calsequestrin 1 (fast-twitch, skeletal muscle)	-0.584	0.004	-0.173	***0.486***	0.027	***0.402***
Dpep1	dipeptidase 1 (renal)	-0.585	0.002	-0.626	0.000	0.013	0.019
Adra2a	adrenergic, alpha-2A-, receptor	-0.585	0.005	-0.281	***0.823***	-0.266	0.022
Ptpro	protein tyrosine phosphatase, receptor type, O	-0.586	0.007	-0.444	0.012	-0.391	0.020
Pld4	phospholipase D family, member 4	-0.586	0.003	-0.013	***0.646***	0.124	***0.680***
Defa10	defensin alpha 10	-0.587	0.010	-0.871	0.003	-0.110	0.039
Efhc2	EF-hand domain (C-terminal) containing 2	-0.587	0.004	-0.309	0.019	-0.230	***0.765***
Sult1c2a	sulfotransferase family, cytosolic, 1C, member 2a	-0.588	0.002	-0.604	0.004	-0.147	0.018
Col11a2	collagen, type XI, alpha 2	-0.589	0.004	0.186	***0.080***	0.061	***0.331***
Thbs2	thrombospondin 2	-0.590	0.003	-0.026	***0.699***	0.150	***0.791***
Cyp8b1	cytochrome P450, family 8, subfamily b, polypeptide 1	-0.590	0.008	-0.629	0.001	-0.226	0.026
Hbegf	heparin-binding EGF-like growth factor	-0.592	0.004	-0.489	0.006	-0.601	0.002
Plp1	proteolipid protein 1	-0.593	0.004	-0.337	0.021	-0.167	***0.923***
Trpm5	transient receptor potential cation channel, subfamily M, member 5	-0.593	0.004	0.185	***0.062***	-0.008	***0.316***
RGD1559960	similar to Sulfotransferase K1 (rSULT1C2)	-0.596	0.004	-0.475	0.005	-0.241	0.026
Sult1c2	sulfotransferase family, cytosolic, 1C, member 2	-0.596	0.003	0.511	0.000	0.562	0.000
Aoc3	amine oxidase, copper containing 3 (vascular adhesion protein 1)	-0.596	0.003	0.127	***0.902***	-0.057	***0.646***
Tyrobp	Tyro protein tyrosine kinase binding protein	-0.596	0.004	0.224	***0.823***	0.267	***0.909***
Cebpe	CCAAT/enhancer binding protein (C/EBP), epsilon	-0.597	0.003	-0.188	***0.940***	0.062	***0.700***
Gpr37	G protein-coupled receptor 37	-0.597	0.004	-0.541	0.002	-0.289	***0.933***
Sp5	Sp5 transcription factor	-0.597	0.005	-0.674	0.001	-0.163	0.042
RGD1307220	similar to RIKEN cDNA E330026B02	-0.599	0.002	0.314	***0.951***	0.207	***0.804***
Hspb7	heat shock protein family, member 7 (cardiovascular)	-0.599	0.005	-0.466	0.008	-0.532	0.002
Rgs9	regulator of G-protein signaling 9	-0.599	0.004	-0.178	***0.643***	-0.065	***0.379***
Mustn1	musculoskeletal, embryonic nuclear protein 1	-0.600	0.004	-0.183	***0.844***	-0.254	***0.856***
Insig1	insulin induced gene 1	-0.601	0.003	-0.356	0.018	-0.164	***0.572***
LOC686151	similar to cell division cycle associated 5	-0.601	0.010	-0.597	0.004	-0.216	0.017
RGD1562284	similar to Glutaminyl-peptide cyclotransferase precursor (QC)	-0.602	0.004	-0.084	***0.449***	-0.140	***0.431***
LOC690326	hypothetical protein LOC690326	-0.603	0.004	-0.533	0.000	-0.469	0.001
Gpr34	G protein-coupled receptor 34	-0.603	0.003	-0.164	***0.700***	0.189	***0.916***
Ckm	creatine kinase, muscle	-0.604	0.002	-0.242	***0.823***	-0.252	***0.757***
Cryba2	crystallin, beta A2	-0.604	0.003	-0.493	0.004	-0.218	***0.853***
Cmtm5	CKLF-like MARVEL transmembrane domain containing 5	-0.606	0.004	0.026	***0.713***	-0.004	***0.468***
Alpl	alkaline phosphatase, liver/bone/kidney	-0.607	0.004	-0.099	***0.629***	-0.258	***0.903***
LOC685125	similar to RIKEN cDNA 5031410I06	-0.607	0.003	0.122	***0.068***	-0.449	0.006
Asb2	ankyrin repeat and SOCS box-containing 2	-0.607	0.004	0.268	***0.851***	0.015	***0.316***
RGD1561849	similar to RIKEN cDNA 3110035E14	-0.609	0.004	-0.276	***0.938***	-0.201	***0.763***
RGD1565033	similar to hypothetical protein LOC284018 isoform b	-0.609	0.003	-0.509	0.005	-0.402	0.007
Agr2	anterior gradient 2 homolog (Xenopus laevis)	-0.610	0.004	-0.360	0.006	-0.422	0.004
Ocm	oncomodulin	-0.610	0.006	-0.930	0.000	-0.273	0.004
LOC685752	hypothetical protein LOC685752	-0.613	0.005	-0.307	0.028	-0.047	***0.256***
Slc6a17	solute carrier family 6 (neurotransmitter transporter), member 17	-0.613	0.007	-0.162	***0.543***	-0.301	***0.765***
Slc16a7	solute carrier family 16, member 7 (monocarboxylic acid transporter 2)	-0.614	0.004	-0.682	0.000	-0.355	0.010
Krtap16-5	keratin associated protein 16-5	-0.615	0.002	-0.028	***0.226***	0.218	0.037
LOC689744	similar to carboxylesterase 2	-0.615	0.003	-0.481	0.005	-0.152	***0.635***

Nr4a3	nuclear receptor subfamily 4, group A, member 3	-0.616	0.012	-0.514	0.045	-1.429	0.000
RGD1562392	similar to Sulfotransferase K1 (rSULT1C2)	-0.616	0.005	0.691	0.000	0.738	0.000
Chst9	carbohydrate (N-acetylgalactosamine 4-0) sulfotransferase 9	-0.616	0.003	-0.441	0.006	-0.325	0.019
LOC690020	similar to killer cell lectin-like receptor, subfamily A, member 17	-0.616	0.004	-0.135	***0.690***	-0.010	***0.419***
Apoe	apolipoprotein E	-0.617	0.042	0.315	0.005	0.306	0.005
Wfikkn2	WAP, follistatin/kazal, immunoglobulin, kunitz and netrin domain containing 2	-0.617	0.004	-0.143	***0.758***	-0.124	***0.783***
Itgb1bp2	integrin beta 1 binding protein 2	-0.617	0.004	-0.408	0.012	-0.230	***0.746***
Rxrg	retinoid X receptor gamma	-0.617	0.004	0.034	***0.547***	0.023	***0.506***
Ramp1	receptor (G protein-coupled) activity modifying protein 1	-0.618	0.003	-0.451	0.004	-0.380	0.016
Tmem38a	transmembrane protein 38a	-0.618	0.003	-0.117	***0.644***	-0.128	***0.770***
Aldh1b1	aldehyde dehydrogenase 1 family, member B1	-0.618	0.002	-0.347	0.014	-0.327	0.013
Cryab	crystallin, alpha B	-0.620	0.003	-0.052	***0.421***	-0.428	0.011
Slc28a2	solute carrier family 28 (sodium-coupled nucleoside transporter), member 2	-0.621	0.002	0.035	***0.400***	0.088	***0.696***
Pycard	PYD and CARD domain containing	-0.621	0.002	0.002	***0.196***	0.195	***0.783***
Pcsk1	proprotein convertase subtilisin/kexin type 1	-0.621	0.003	0.120	***0.053***	-0.373	0.004
Palmd	palmdelphin	-0.623	0.005	-0.494	0.004	-0.652	0.001
Prelid2	PRELI domain containing 2	-0.623	0.002	-0.519	0.002	-0.177	***0.682***
Tnnt1	troponin T type 1 (skeletal, slow)	-0.623	0.005	-0.063	0.025	0.365	0.016
Fndc5	fibronectin type III domain containing 5	-0.624	0.003	-0.052	***0.509***	-0.008	***0.581***
Ptpro	protein tyrosine phosphatase, receptor type, O	-0.625	0.006	-0.477	0.007	-0.479	0.009
Abca8a	ATP-binding cassette, subfamily A (ABC1), member 8a	-0.625	0.004	-0.102	***0.450***	-0.214	***0.591***
Edn3	endothelin 3	-0.626	0.014	-0.573	0.010	-0.460	0.011
Aif1	allograft inflammatory factor 1	-0.627	0.002	0.308	***0.927***	0.349	0.048
Cd44	Cd44 molecule	-0.628	0.003	-0.791	0.000	-0.550	0.002
Muc1	mucin 1, cell surface associated	-0.628	0.002	-0.408	0.012	0.154	***0.669***
Aqp9	aquaporin 9	-0.628	0.002	0.156	***0.893***	0.289	***0.051***
Mmd2	monocyte to macrophage differentiation-associated 2	-0.628	0.003	0.002	***0.547***	-0.043	***0.728***
Fos	FBJ osteosarcoma oncogene	-0.629	0.003	-0.868	0.000	-0.816	0.000
Zp2	zona pellucida glycoprotein 2 (sperm receptor)	-0.630	0.002	-0.180	0.008	0.256	0.001
MGC105567	similar to cDNA sequence BC023105	-0.630	0.001	0.126	***0.618***	0.122	***0.728***
Nucb2	nucleobindin 2	-0.632	0.002	-0.228	***0.913***	-0.178	***0.812***
Tnnt1	troponin T type 1 (skeletal, slow)	-0.632	0.003	-0.067	***0.463***	0.172	***0.498***
Adra1a	adrenergic, alpha-1A-, receptor	-0.632	0.034	-0.502	0.032	0.082	***0.091***
RGD1561459	similar to RIKEN cDNA 1810020D17	-0.632	0.002	-0.506	0.001	-0.389	0.010
Gucy1a3	guanylate cyclase 1, soluble, alpha 3	-0.632	0.002	-0.020	***0.360***	-0.044	***0.319***
Gpihbp1	glycosylphosphatidylinositol anchored high density lipoprotein binding protein 1	-0.633	0.002	0.019	***0.429***	-0.058	***0.663***
Lilra5	leukocyte immunoglobulin-like receptor, subfamily A (with TM domain), member 5	-0.634	0.002	-0.144	***0.871***	0.148	***0.911***
Tmem45a	transmembrane protein 45A	-0.634	0.004	-0.465	0.002	-0.426	0.003
RGD1561551	similar to Hypothetical protein MGC75664	-0.634	0.003	-0.598	0.003	-0.459	0.012
Spock1	sparc/osteonectin, cwcv and kazal-like domains proteoglycan (testican) 1	-0.637	0.005	-0.250	***0.538***	-0.118	***0.281***
Tmem72	transmembrane protein 72	-0.637	0.002	-0.359	0.009	-0.053	***0.281***
Ckmt2	creatine kinase, mitochondrial 2, sarcomeric	-0.637	0.002	-0.326	0.037	0.118	***0.812***
Car1	carbonic anhydrase 1	-0.637	0.003	-0.272	0.000	0.239	0.003
RGD1311946	similar to RIKEN cDNA 1810055G02	-0.638	0.006	-0.467	0.004	-0.464	0.007
Fer1l4	fer-1-like 4 (C. elegans)	-0.639	0.003	0.254	***0.392***	0.623	0.001
Chgb	chromogranin B	-0.640	0.004	-0.372	0.005	-0.105	***0.279***
Dgat2	diacylglycerol O-acyltransferase 2	-0.643	0.003	-0.365	0.009	-0.065	***0.419***
Zg16	zymogen granule protein 16	-0.643	0.003	-0.241	0.018	-0.412	0.005
Igf2	insulin-like growth factor 2	-0.644	0.003	-0.039	***0.611***	0.090	***0.608***
C1qtnf9	C1q and tumor necrosis factor related protein 9	-0.645	0.007	-0.290	***0.690***	-0.204	***0.528***
Cxcr6	chemokine receptor CXCR6	-0.645	0.018	-0.257	***0.208***	0.052	0.033
Apoa4	apolipoprotein A-IV	-0.645	0.000	-1.955	0.000	-1.273	0.000
Slc22a1	solute carrier family 22 (organic cation transporter), member 1	-0.646	0.002	-0.159	***0.570***	-0.119	***0.467***
Bag2	Bcl2-associated athanogene 2	-0.647	0.001	-0.280	***0.752***	-0.482	0.004
Mustn1	musculoskeletal, embryonic nuclear protein 1	-0.647	0.002	-0.166	***0.918***	-0.284	***0.980***
Mmp23	matrix metallopeptidase 23	-0.647	0.001	0.029	***0.463***	-0.026	***0.646***
Myom1	myomesin 1	-0.647	0.003	-0.097	***0.832***	-0.096	***0.649***

LOC681458	similar to stearoyl-coenzyme A desaturase 3	-0.648	0.014	0.176	0.027	-0.093	***0.091***
Car11	carbonic anhydrase 11	-0.649	0.003	0.269	***0.960***	0.005	***0.439***
MGC109340	similar to Microsomal signal peptidase 23 kDa subunit (SPase 22 kDa subunit) (SPC22/23)	-0.649	0.003	-0.312	0.034	-0.371	0.011
Il1b	interleukin 1 beta	-0.649	0.001	0.221	***0.361***	0.249	***0.616***
C2cd4d	C2 calcium-dependent domain containing 4D	-0.650	0.002	-0.247	***0.927***	-0.410	0.009
Cpe	carboxypeptidase E	-0.653	0.002	-0.457	0.006	-0.235	***0.843***
C4b	complement component 4B (Chido blood group)	-0.654	0.002	0.226	0.021	0.415	0.003
Klk1c6	kallikrein 1-related peptidase C6	-0.654	0.003	-0.662	0.000	-0.244	0.028
Isg20	interferon stimulated exonuclease gene 20	-0.654	0.002	-0.311	0.022	-0.296	***0.940***
Tmem116	transmembrane protein 116	-0.656	0.002	-0.368	0.026	-0.219	***0.795***
LOC688542	carboxylesterase 5-like	-0.658	0.002	-0.308	0.001	-0.184	0.023
RGD1307461	similar to RIKEN cDNA 6430571L13 gene; similar to g20 protein	-0.658	0.002	-0.866	0.000	-0.771	0.000
Dner	delta/notch-like EGF repeat containing	-0.659	0.004	-0.157	***0.510***	-0.025	***0.296***
Nxph3	neurexophilin 3	-0.659	0.005	-0.110	***0.310***	-0.036	***0.250***
Prr5l	proline rich 5 like	-0.659	0.003	-0.611	0.001	-0.183	0.039
Kif6	kinesin family member 6	-0.659	0.005	-0.557	0.003	-0.228	***0.432***
Noxa1	NADPH oxidase activator 1	-0.659	0.001	-0.241	***0.890***	-0.110	***0.656***
Reg4	regenerating islet-derived family, member 4	-0.660	0.002	-0.262	***0.820***	-0.276	***0.783***
Dpt	dermatopontin	-0.660	0.001	-0.088	0.029	-0.540	0.000
Emp1	epithelial membrane protein 1	-0.660	0.003	-0.494	0.001	-0.124	***0.053***
Sparcl1	SPARC-like 1 (hevin)	-0.664	0.001	-0.063	***0.412***	-0.156	***0.497***
Tpm1	tropomyosin 1, alpha	-0.664	0.002	-0.142	***0.674***	-0.348	0.016
LOC500827	similar to hypothetical protein FLJ35821	-0.664	0.003	-0.595	0.002	-0.277	0.030
Pcp4	Purkinje cell protein 4	-0.665	0.000	-0.151	***0.784***	-0.084	***0.513***
Crym	crystallin, mu	-0.666	0.002	-0.839	0.000	-0.253	0.028
Sqle	squalene epoxidase	-0.669	0.002	-0.539	0.002	-0.334	0.025
Hspb3	heat shock protein 3	-0.672	0.002	-0.663	0.000	-0.395	0.004
Spink8	serine peptidase inhibitor, Kazal type 8	-0.672	0.001	-0.134	***0.700***	-0.140	***0.692***
Abcb1a	ATP-binding cassette, sub-family B (MDR/TAP), member 1A	-0.673	0.002	0.084	0.036	0.267	0.025
Slc28a2	solute carrier family 28 (sodium-coupled nucleoside transporter), member 2	-0.673	0.002	-0.083	***0.537***	0.100	***0.830***
LOC679368	similar to carboxylesterase 5	-0.677	0.002	-0.347	0.001	-0.400	0.006
RGD1563975	similar to testis expressed sequence 13B	-0.678	0.010	-0.729	0.003	-0.204	***0.348***
Mpz	myelin protein zero	-0.679	0.021	-0.583	0.013	-0.103	***0.138***
Fabp3	fatty acid binding protein 3, muscle and heart	-0.681	0.001	-0.459	0.012	-0.457	0.009
Fxyd6	FXYD domain-containing ion transport regulator 6	-0.682	0.001	-0.184	***0.852***	-0.232	***0.938***
LOC100363825	rCG56028-like	-0.682	0.002	-0.028	0.008	0.257	0.015
LOC690948	similar to paired-Ig-like receptor A11	-0.682	0.003	-0.008	***0.477***	0.021	***0.524***
LOC690097	similar to immunoreceptor Ly49si3	-0.683	0.002	-0.026	***0.344***	0.286	***0.069***
Upk1b	uroplakin 1B	-0.684	0.000	0.327	0.008	0.310	0.006
Selm	selenoprotein M	-0.686	0.001	-0.366	0.011	-0.222	***0.783***
Snhg11	small nucleolar RNA host gene 11	-0.686	0.001	0.296	***0.955***	0.176	***0.733***
Sytl3	synaptotagmin-like 3	-0.687	0.001	-0.097	***0.700***	0.048	***0.601***
Tgfb3	transforming growth factor, beta 3	-0.687	0.003	-0.187	***0.422***	-0.328	***0.689***
LOC687856	similar to Myeloid cell surface antigen CD33 precursor (Siglec-3)	-0.688	0.002	0.116	***0.746***	0.161	***0.867***
LOC499465	hypothetical protein LOC499465	-0.688	0.002	-0.302	0.027	-0.305	***0.606***
Plcd4	phospholipase C, delta 4	-0.689	0.002	-0.228	***0.673***	-0.250	***0.762***
Plcd4	phospholipase C, delta 4	-0.690	0.002	-0.295	0.030	-0.278	***0.836***
Abp1	amiloride binding protein 1 (amine oxidase, copper-containing)	-0.693	0.002	-0.211	***0.696***	-0.262	***0.760***
Arc	activity-regulated cytoskeleton-associated protein	-0.694	0.007	-0.277	***0.469***	-0.503	0.014
Fxyd2	FXYD domain-containing ion transport regulator 2	-0.695	0.001	-0.033	***0.364***	-0.163	***0.611***
Tlr11	toll-like receptor 11	-0.697	0.001	0.116	***0.721***	0.224	***0.808***
Emr1	EGF-like module containing, mucin-like, hormone receptor-like 1	-0.699	0.001	0.187	***0.784***	0.133	***0.657***
Mamdc2	MAM domain containing 2	-0.701	0.002	-0.187	***0.062***	-0.218	0.048
Igsf11	immunoglobulin superfamily, member 11	-0.701	0.001	-0.550	0.003	-0.125	0.050
Tlr12	toll-like receptor 12	-0.702	0.001	0.113	***0.388***	0.377	0.028
Ddc	dopa decarboxylase (aromatic L-amino acid decarboxylase)	-0.703	0.001	-0.555	0.001	-0.092	***0.671***
Mcpt3	mast cell peptidase 3	-0.703	0.002	0.195	***0.322***	0.507	0.005
Olr1566	olfactory receptor 1566	-0.704	0.005	-0.605	0.005	-0.177	***0.325***
Pbp2	phosphatidylethanolamine binding protein 2	-0.704	0.003	-0.672	0.001	-0.166	***0.051***

Tac2	tachykinin 2	-0.704	0.001	-0.147	***0.871***	-0.190	***0.892***
Ptgs2	prostaglandin-endoperoxide synthase 2	-0.710	0.002	-0.147	***0.350***	-0.219	***0.601***
Tekt4	tektin 4	-0.711	0.004	-0.608	0.001	-0.136	0.010
Srgn	serglycin	-0.711	0.001	-0.022	***0.275***	0.277	0.030
Atp6v0a4	ATPase, H+ transporting, lysosomal V0 subunit A4	-0.712	0.002	-0.448	0.006	-0.262	0.031
Pi16	peptidase inhibitor 16	-0.712	0.002	-0.180	***0.361***	-0.323	0.021
LOC685716	similar to OX-2 membrane glycoprotein precursor (MRC OX-2 antigen) (CD200 antigen)	-0.712	0.001	-0.490	0.004	0.049	***0.400***
Ltbp2	latent transforming growth factor beta binding protein 2	-0.712	0.002	-0.493	0.010	-0.382	0.025
Celf3	CUGBP, Elav-like family member 3	-0.713	0.004	-0.660	0.001	-0.473	0.004
Fcer1g	Fc fragment of IgE, high affinity I, receptor for; gamma polypeptide	-0.714	0.001	0.105	***0.694***	0.122	***0.726***
Etv5	ets variant 5	-0.715	0.002	-0.704	0.000	-0.382	0.008
Artn	artemin	-0.715	0.001	-0.333	0.039	-0.215	***0.934***
Glycam1	glycosylation dependent cell adhesion molecule 1	-0.716	0.000	-0.839	0.001	0.860	0.000
Herc6	hect domain and RLD 6	-0.719	0.001	-0.159	***0.843***	0.028	***0.626***
Dnase1l3	deoxyribonuclease 1-like 3	-0.720	0.002	0.313	0.027	0.413	0.011
Inmt	indolethylamine N-methyltransferase	-0.722	0.002	-0.167	***0.541***	-0.247	***0.696***
Mfap4	microfibrillar-associated protein 4	-0.725	0.001	0.099	***0.787***	0.003	***0.460***
Alkbh6	alkB, alkylation repair homolog 6 (E. coli)	-0.728	0.001	-0.279	***0.951***	-0.127	***0.589***
Tnfrsf11b	tumor necrosis factor receptor superfamily, member 11b	-0.730	0.001	-0.626	0.002	-0.987	0.000
Fam5c	family with sequence similarity 5, member C	-0.730	0.002	-0.219	0.036	-0.348	***0.781***
Fxyd6	FXYD domain-containing ion transport regulator 6	-0.733	0.001	-0.174	***0.889***	-0.305	***0.974***
Ifi27l2b	interferon, alpha-inducible protein 27 like 2B	-0.733	0.001	-0.270	***0.516***	0.061	0.048
RGD1559482	similar to immunoglobulin superfamily, member 7	-0.733	0.001	-0.045	***0.660***	0.042	***0.366***
Slc22a1	solute carrier family 22 (organic cation transporter), member 1	-0.733	0.001	-0.178	***0.635***	-0.064	***0.404***
Ptpns1l3	protein tyrosine phosphatase, non-receptor type substrate 1-like 3	-0.734	0.001	0.062	***0.732***	-0.079	***0.607***
Nrbp2	nuclear receptor binding protein 2	-0.735	0.001	0.002	***0.577***	0.017	***0.460***
Ifi27l2b	interferon, alpha-inducible protein 27 like 2B	-0.736	0.001	-0.243	***0.453***	0.016	***0.077***
Igfbp3	insulin-like growth factor binding protein 3	-0.737	0.001	0.285	***0.862***	0.037	***0.397***
B3gnt6	UDP-GlcNAc:betaGal beta-1,3-N-acetylglucosaminyltransferase 6 (core 3 synthase)	-0.737	0.002	-0.548	0.003	-0.425	0.013
Efcab6	EF-hand calcium binding domain 6	-0.739	0.002	-0.465	0.002	-0.448	0.001
Lipt2	lipoyl(octanoyl) transferase 2 (putative)	-0.740	0.016	-0.790	0.003	-0.218	0.022
RGD1561778	similar to dendritic cell-derived immunoglobulin(Ig)-like receptor 1, DIgR1 - mouse	-0.742	0.001	-0.020	***0.643***	0.138	***0.609***
Dnajc10	DnaJ (Hsp40) homolog, subfamily C, member 10	-0.744	0.001	-0.396	0.012	-0.405	0.012
RGD1560700	similar to palmitoyl-protein thioesterase	-0.744	0.000	-1.842	0.000	-1.172	0.000
Chrdl2	chordin-like 2	-0.744	0.001	-0.329	***0.877***	-0.508	0.003
Fam198b	family with sequence similarity 198, member B	-0.745	0.001	-0.267	***0.888***	-0.294	***0.849***
LOC498480	similar to Spetex-2C protein	-0.745	0.001	-0.182	0.024	0.092	***0.080***
Casp1	caspase 1	-0.746	0.001	-0.368	0.005	-0.161	***0.545***
Fkbp11	FK506 binding protein 11	-0.749	0.001	-0.583	0.002	-0.283	***0.671***
Ccnjl	cyclin J-like	-0.750	0.001	-0.341	0.022	-0.071	***0.356***
RGD1565709	similar to ovostatin-2	-0.751	0.005	-0.828	0.001	-0.426	0.007
Snap25	synaptosomal-associated protein 25	-0.753	0.001	0.159	***0.482***	-0.064	***0.497***
Ankrd1	ankyrin repeat domain 1	-0.753	0.004	-1.022	0.000	-1.264	0.000
Msmo1	methylsterol monooxygenase 1	-0.754	0.001	-0.476	0.006	-0.232	***0.800***
Nrbp2	nuclear receptor binding protein 2	-0.756	0.001	0.044	***0.727***	0.024	***0.405***
RGD1305627	hypothetical LOC314467	-0.757	0.001	-0.599	0.001	-0.276	***0.973***
C3	complement component 3	-0.757	0.010	0.090	0.011	0.360	0.001
RGD1562551	similar to C20orf82	-0.758	0.001	-0.287	***0.747***	-0.355	***0.827***
Nat8l	N-acetyltransferase 8-like	-0.759	0.003	0.121	***0.416***	-0.191	***0.379***
Aif1	allograft inflammatory factor 1	-0.759	0.001	0.225	***0.648***	0.296	***0.056***
Hrc	histidine rich calcium binding protein	-0.759	0.001	-0.548	0.003	-0.193	***0.678***
RGD1305645	similar to RIKEN cDNA 1500015O10	-0.760	0.001	-0.067	***0.428***	-0.103	***0.741***
Ccdc19	coiled-coil domain containing 19	-0.761	0.001	-0.547	0.004	-0.416	0.013
Selm	selenoprotein M	-0.761	0.001	-0.424	0.009	-0.229	***0.816***
RGD1561459	similar to RIKEN cDNA 1810020D17	-0.762	0.001	-0.463	0.003	-0.267	***0.788***
Muc2	mucin 2, oligomeric mucus/gel-forming	-0.763	0.001	-0.760	0.000	0.085	***0.067***
Klk1c8	kallikrein 1-related peptidase C8	-0.763	0.001	-0.724	0.000	-0.404	0.008
Umod	uromodulin	-0.763	0.001	-0.440	0.006	-0.392	0.013
Cbs	cystathionine beta synthase	-0.766	0.001	-0.313	***0.948***	-0.169	***0.770***

Tmem45a	transmembrane protein 45A	-0.771	0.001	-0.519	0.002	-0.346	0.007
Hpgds	hematopoietic prostaglandin D synthase	-0.772	0.001	0.015	***0.427***	0.139	***0.699***
Clec4a2	C-type lectin domain family 4, member A2	-0.774	0.001	0.002	***0.245***	0.013	***0.417***
Kb23	type II keratin Kb23	-0.776	0.001	-0.462	0.005	-0.468	0.005
Vipr2	vasoactive intestinal peptide receptor 2	-0.781	0.001	-0.112	***0.628***	-0.071	***0.472***
Htr2b	5-hydroxytryptamine (serotonin) receptor 2B	-0.781	0.001	-0.034	***0.367***	-0.130	***0.521***
LOC688256	similar to paired immunoglobin-like type 2 receptor beta	-0.781	0.017	-0.883	0.003	-0.037	***0.098***
Cpxm2	carboxypeptidase X (M14 family), member 2	-0.782	0.000	-0.079	***0.723***	-0.080	***0.604***
Sncg	synuclein, gamma (breast cancer-specific protein 1)	-0.782	0.001	-0.147	***0.765***	-0.178	***0.796***
Gsta3	glutathione S-transferase A3	-0.783	0.001	0.139	***0.656***	-0.017	***0.466***
RGD1564571	similar to DC-SIGN	-0.783	0.006	-0.084	0.049	0.150	0.016
Adora3	adenosine A3 receptor	-0.784	0.000	0.095	***0.647***	0.268	***0.947***
RT1-T24-1	RT1 class I, locus T24, gene 1	-0.785	0.001	-0.128	***0.705***	-0.225	***0.881***
Fcer1g	Fc fragment of IgE, high affinity I, receptor for; gamma polypeptide	-0.786	0.001	-0.016	***0.563***	0.062	***0.649***
Tac1	tachykinin 1	-0.788	0.000	-0.163	***0.586***	-0.128	***0.634***
Chst4	carbohydrate (N-acetylglucosamine 6-O) sulfotransferase 4	-0.788	0.001	-0.695	0.000	-0.165	***0.698***
Sik1	salt-inducible kinase 1	-0.789	0.001	-1.043	0.000	-1.256	0.000
LOC690020	similar to killer cell lectin-like receptor, subfamily A, member 17	-0.789	0.001	-0.065	***0.542***	-0.116	***0.562***
F13a1	coagulation factor XIII, A1 polypeptide	-0.792	0.001	0.010	***0.405***	0.057	***0.461***
Tmprss8	transmembrane protease, serine 8	-0.792	0.000	-0.626	0.001	-0.458	0.003
Myh8	myosin, heavy chain 8, skeletal muscle, perinatal	-0.794	0.000	-0.812	0.000	-0.793	0.000
Rcan1	regulator of calcineurin 1	-0.799	0.001	-0.432	0.018	-0.707	0.000
Adra1d	adrenergic, alpha-1D-, receptor	-0.803	0.004	-0.365	0.021	-0.367	0.020
Txn1	thioredoxin 1	-0.803	0.000	-0.091	0.005	-0.297	0.014
Hoxa10	homeo box A10	-0.805	0.002	-0.181	0.031	-0.177	0.041
Abo2	histo-blood group ABO system transferase 2	-0.807	0.001	-0.579	0.001	-0.096	***0.058***
Prdx6	peroxiredoxin 6	-0.807	0.001	-0.687	0.000	0.005	0.045
Tmed6	transmembrane emp24 protein transport domain containing 6	-0.808	0.001	-0.465	0.004	-0.129	***0.053***
Klk1	kallikrein 1	-0.809	0.001	-0.637	0.000	-0.286	0.018
Dnmt3l	DNA (cytosine-5-)-methyltransferase 3-like	-0.810	0.000	-0.185	***0.614***	0.074	***0.426***
Gpc2	glypican 2	-0.810	0.000	-0.328	0.032	-0.210	***0.669***
Sostdc1	sclerostin domain containing 1	-0.811	0.000	-0.288	***0.967***	-0.253	***0.977***
Sult1c2a	sulfotransferase family, cytosolic, 1C, member 2a	-0.811	0.000	-0.610	0.001	-0.289	0.007
LOC680029	similar to Spetex-2F protein	-0.817	0.001	-0.205	0.012	-0.092	***0.059***
Trdn	triadin	-0.817	0.001	-0.156	***0.380***	0.267	0.016
Itm2a	integral membrane protein 2A	-0.819	0.000	0.234	***0.938***	0.053	***0.665***
Cbr1	carbonyl reductase 1	-0.821	0.001	-0.455	0.004	-0.493	0.003
Clec9a	C-type lectin domain family 9, member A	-0.826	0.000	0.011	***0.389***	0.368	0.033
Chmp4bl1	chromatin modifying protein 4B-like 1	-0.826	0.003	-1.149	0.000	-1.016	0.000
LOC688173	similar to Telethonin (Titin cap protein)	-0.829	0.001	-0.372	0.034	-0.112	***0.055***
Klra17	killer cell lectin-like receptor, subfamily A, member 17	-0.829	0.001	0.000	***0.317***	0.381	0.035
Abra	actin-binding Rho activating protein	-0.830	0.000	-0.490	0.006	-0.664	0.000
C7	complement component 7	-0.838	0.000	0.152	***0.679***	0.222	***0.915***
Klk1c7	kallikrein 1-related peptidase C7	-0.839	0.001	-0.724	0.000	-0.309	0.020
Wfdc1	WAP four-disulfide core domain 1	-0.842	0.000	-0.650	0.001	-0.249	***0.922***
Ltbp2	latent transforming growth factor beta binding protein 2	-0.844	0.000	-0.663	0.001	-0.597	0.001
Ddc	dopa decarboxylase (aromatic L-amino acid decarboxylase)	-0.853	0.000	-0.621	0.001	-0.143	***0.862***
Pdgfrl	platelet-derived growth factor receptor-like	-0.853	0.000	-0.228	***0.619***	-0.132	***0.379***
Rsad2	radical S-adenosyl methionine domain containing 2	-0.854	0.001	-0.174	0.001	0.814	0.000
RGD1563091	similar to OEF2	-0.855	0.001	-0.267	***0.596***	0.066	***0.062***
Rpa3	replication protein A3	-0.856	0.000	-0.678	0.000	-0.413	0.007
Cgref1	cell growth regulator with EF hand domain 1	-0.863	0.001	-0.758	0.000	-0.442	0.003
Pygm	phosphorylase, glycogen, muscle	-0.864	0.000	-0.146	***0.823***	-0.051	***0.601***
Akap5	A kinase (PRKA) anchor protein 5	-0.867	0.000	-0.169	***0.840***	-0.180	***0.856***
Lpl	lipoprotein lipase	-0.868	0.000	-0.102	***0.481***	-0.299	***0.879***
Rcan1	regulator of calcineurin 1	-0.873	0.000	-0.478	0.011	-0.673	0.001
Actg2	actin, gamma 2, smooth muscle, enteric	-0.874	0.001	-0.469	0.029	-0.643	0.005
Gsg1l	GSG1-like	-0.876	0.000	-0.200	***0.677***	-0.180	***0.627***

Tubal3	tubulin, alpha-like 3	-0.878	0.001	-1.052	0.000	-0.193	0.002
LOC494527	11 kDa protein 2	-0.879	0.001	-0.951	0.000	-0.962	0.000
Ctxn3	cortexin 3	-0.880	0.000	-0.421	0.013	-0.106	***0.486***
Muc2	mucin 2, oligomeric mucus/gel-forming	-0.884	0.000	-0.534	0.002	-0.243	0.013
Zcchc12	zinc finger, CCHC domain containing 12	-0.884	0.001	-0.526	0.002	-0.286	0.017
LOC691277	similar to Robo-1	-0.885	0.000	-0.429	0.004	0.054	0.018
Lix1	Lix1 homolog (chicken)	-0.885	0.000	0.008	***0.522***	-0.074	***0.719***
Grhpr	glyoxylate reductase/hydroxypyruvate reductase	-0.890	0.000	-0.717	0.000	-0.328	0.010
Rgs5	regulator of G-protein signaling 5	-0.891	0.000	-0.383	0.006	-0.223	***0.723***
Abhd16b	abhydrolase domain containing 16B	-0.892	0.023	-1.153	0.004	-0.859	0.015
Nr4a1	nuclear receptor subfamily 4, group A, member 1	-0.895	0.000	-0.729	0.000	-1.407	0.000
Cldn15	claudin 15	-0.895	0.000	-0.679	0.000	-0.346	0.006
Ptn	pleiotrophin	-0.895	0.000	0.051	***0.361***	-0.084	***0.587***
Cd5l	Cd5 molecule-like	-0.903	0.018	0.222	0.043	0.016	0.015
LOC686384	similar to Kif19A CG9913-PB, isoform B	-0.903	0.021	-0.889	0.009	-0.324	0.024
Etv5	ets variant 5	-0.910	0.000	-0.785	0.000	-0.577	0.001
Clec4a3	C-type lectin domain family 4, member A3	-0.912	0.000	-0.220	***0.746***	0.086	***0.513***
Alox12e	arachidonate 12-lipoxygenase, epidermal	-0.914	0.000	-0.203	***0.765***	0.194	***0.809***
Ms4a7	membrane-spanning 4-domains, subfamily A, member 7	-0.916	0.000	0.386	0.013	0.387	0.007
Rgs11	regulator of G-protein signaling 11	-0.916	0.000	0.115	***0.390***	-0.291	0.014
Rec8	REC8 homolog (yeast)	-0.918	0.000	-0.714	0.000	-0.078	***0.345***
Cd52	CD52 antigen	-0.920	0.000	0.011	***0.535***	0.442	0.012
RGD1562533	similar to mKIAA0774 protein	-0.922	0.000	-0.511	0.005	-0.419	0.009
Clec11a	C-type lectin domain family 11, member A	-0.924	0.000	0.009	***0.371***	-0.024	***0.216***
Itln1	intelectin 1 (galactofuranose binding)	-0.929	0.000	0.021	0.022	0.713	0.000
Btg4	B-cell translocation gene 4	-0.930	0.004	-1.054	0.001	-0.157	0.015
Fcnb	ficolin B	-0.932	0.009	-0.382	0.022	0.090	0.049
Fabp2	fatty acid binding protein 2, intestinal	-0.933	0.000	-0.696	0.000	-0.242	0.009
Slamf9	SLAM family member 9	-0.934	0.000	-0.027	***0.488***	0.135	***0.656***
Hmgcs1	3-hydroxy-3-methylglutaryl-CoA synthase 1 (soluble)	-0.939	0.000	-0.747	0.000	-0.426	0.005
Serpinf1	serpin peptidase inhibitor, clade F (alpha-2 antiplasmin, pigment epithelium derived factor), member 1	-0.941	0.000	-0.003	***0.535***	-0.168	***0.792***
F3	coagulation factor III (thromboplastin, tissue factor)	-0.942	0.000	-0.586	0.001	-0.554	0.001
Tff2	trefoil factor 2	-0.943	0.001	-0.817	0.000	-0.455	0.002
Epsti1	epithelial stromal interaction 1 (breast)	-0.944	0.000	-0.526	0.002	-0.280	***0.791***
LOC494527	11 kDa protein 2	-0.945	0.002	-0.363	0.012	-0.114	0.029
Spp1	secreted phosphoprotein 1	-0.945	0.000	-0.702	0.000	-0.199	***0.744***
Actg2	actin, gamma 2, smooth muscle, enteric	-0.946	0.000	-0.512	0.018	-0.583	0.007
Bpifb6	BPI fold containing family B, member 6	-0.946	0.000	-0.674	0.000	-0.385	0.002
Sectm1a	secreted and transmembrane 1A	-0.959	0.000	-0.167	***0.754***	-0.033	***0.451***
Tapbpl	TAP binding protein-like	-0.960	0.013	-1.189	0.001	-1.409	0.001
Il12a	interleukin 12A	-0.965	0.000	-0.384	0.013	-0.650	0.000
Cacna1h	calcium channel, voltage-dependent, T type, alpha 1H subunit	-0.965	0.001	-0.431	0.012	-0.209	0.023
G0s2	G0/G1switch 2	-0.967	0.000	-0.115	***0.691***	-0.334	***0.978***
Cox6a2	cytochrome c oxidase subunit VIa polypeptide 2	-0.971	0.000	-0.463	0.004	-0.215	***0.862***
Olr621	olfactory receptor 621	-0.972	0.019	-0.751	0.020	-0.235	***0.101***
Idi1	isopentenyl-diphosphate delta isomerase 1	-0.973	0.000	-0.821	0.000	-0.354	0.016
Aldob	aldolase B, fructose-bisphosphate	-0.973	0.000	-0.443	0.001	0.043	0.024
Derl3	Der1-like domain family, member 3	-0.975	0.000	0.181	***0.671***	0.661	0.001
Steap1	six transmembrane epithelial antigen of the prostate 1	-0.975	0.000	-0.830	0.000	-0.575	0.001
LOC691277	similar to Robo-1	-0.976	0.000	-0.490	0.002	0.086	0.022
Cndp1	carnosine dipeptidase 1 (metallopeptidase M20 family)	-0.978	0.000	-0.429	0.004	-0.215	0.025
Masp1	mannan-binding lectin serine peptidase 1	-0.979	0.018	-0.804	0.028	-0.194	0.040
RT1-CE5	RT1 class I, locus CE5	-0.980	0.001	-0.509	0.028	-0.717	0.004
Gpr149	G protein-coupled receptor 149	-0.981	0.000	-0.490	0.001	-0.589	0.001
Nkg7	natural killer cell group 7 sequence	-0.981	0.000	-0.027	***0.633***	0.070	***0.683***
Cyp4f40	cytochrome P450, family 4, subfamily f, polypeptide 40	-0.981	0.000	-0.444	0.000	0.058	0.003
Cd5l	Cd5 molecule-like	-0.983	0.028	0.285	0.018	0.075	0.031
Ctgf	connective tissue growth factor	-0.984	0.000	-0.153	0.001	-0.618	0.000

Capn8	calpain 8	-0.997	0.000	-0.587	0.002	-0.125	0.047
KIFC2	kinesin family member C2	-0.998	0.000	-0.453	0.007	-0.171	***0.836***
Syce2	synaptonemal complex central element protein 2	-1.000	0.029	-1.117	0.002	0.028	***0.087***
Chga	chromogranin A	-1.011	0.000	-0.328	0.001	-0.133	0.041
Smpx	small muscle protein, X-linked	-1.016	0.000	-0.349	0.025	-0.381	0.018
Myl2	myosin, light polypeptide 2, regulatory, cardiac, slow	-1.019	0.000	-0.940	0.000	-0.823	0.000
Ptgis	prostaglandin I2 (prostacyclin) synthase	-1.026	0.000	-0.370	0.015	-0.173	***0.515***
Cfd	complement factor D (adipsin)	-1.037	0.000	-0.087	***0.070***	0.009	0.038
Msln	mesothelin	-1.041	0.000	0.382	0.000	0.377	0.001
LOC360228	WDNM1 homolog	-1.041	0.000	0.559	0.001	0.231	0.005
Kcnj5	potassium inwardly-rectifying channel, subfamily J, member 5	-1.043	0.000	-1.062	0.000	-0.766	0.000
Car3	carbonic anhydrase 3	-1.045	0.000	0.278	0.004	-0.263	0.004
Slpil2	antileukoproteinase-like 2	-1.050	0.000	0.102	***0.233***	0.425	0.020
Ca2	carbonic anhydrase 2	-1.055	0.000	-0.551	0.000	-0.087	0.028
Rgs2	regulator of G-protein signaling 2	-1.055	0.000	-0.909	0.000	-0.845	0.000
Rgs2	regulator of G-protein signaling 2	-1.059	0.000	-0.856	0.001	-0.760	0.001
Mfsd2a	major facilitator superfamily domain containing 2A	-1.059	0.000	-0.455	0.011	-0.262	***0.852***
Tnfrsf12a	tumor necrosis factor receptor superfamily, member 12a	-1.059	0.000	-0.600	0.003	-1.353	0.000
Car8	carbonic anhydrase 8	-1.060	0.000	-0.525	0.002	-0.339	0.010
Slfn3	schlafen 3	-1.060	0.000	0.308	0.018	0.362	0.009
Mybpc2	myosin binding protein C, fast-type	-1.064	0.000	-0.459	0.011	-0.353	***0.929***
Gcnt3	glucosaminyl (N-acetyl) transferase 3, mucin type	-1.071	0.000	-0.549	0.003	-0.183	0.015
Mrap	melanocortin 2 receptor accessory protein	-1.072	0.001	-0.045	0.036	-0.363	0.032
Tmprss9	transmembrane protease, serine 9	-1.072	0.000	-1.083	0.000	-0.683	0.000
Tmprss9	transmembrane protease, serine 9	-1.077	0.000	-1.024	0.000	-0.582	0.001
Gldn	gliomedin	-1.085	0.000	-0.522	0.003	-0.161	***0.694***
Hsd3b6	hydroxy-delta-5-steroid dehydrogenase, 3 beta- and steroid delta-isomerase 6	-1.086	0.000	-0.518	0.000	-0.228	0.000
Casq1	calsequestrin 1 (fast-twitch, skeletal muscle)	-1.086	0.000	-0.436	0.011	-0.282	***0.886***
RGD1560314	RGD1560314	-1.089	0.008	-0.959	0.003	-0.119	***0.081***
RGD1309651	similar to 1190005I06Rik protein	-1.094	0.000	-0.426	0.017	-0.103	***0.630***
Ly49i2	Ly49 inhibitory receptor 2	-1.102	0.001	-0.604	0.002	-1.177	0.000
Tph1	tryptophan hydroxylase 1	-1.102	0.000	-0.603	0.001	-0.298	0.025
LOC363060	similar to RIKEN cDNA 1600029D21	-1.108	0.000	-0.829	0.000	-0.616	0.001
Gsta5	glutathione S-transferase Yc2 subunit	-1.111	0.000	-0.107	***0.608***	0.033	***0.357***
LOC688635	similar to Spetex-2F protein	-1.113	0.000	-0.189	0.015	-0.038	***0.085***
RGD1562035	similar to mast cell protease 1-like 3 precursor	-1.116	0.000	-0.261	***0.774***	0.046	***0.522***
Kif1c	kinesin family member 1C	-1.125	0.007	-0.943	0.005	-0.701	0.046
LOC687842	similar to Cytochrome P450 2C24 (CYPIIC24) (P450-PROS2)	-1.126	0.000	-0.830	0.000	-0.489	0.000
Scx	scleraxis	-1.131	0.000	-0.368	0.022	-0.413	0.018
Ccl11	chemokine (C-C motif) ligand 11	-1.133	0.000	-0.413	0.016	-0.028	***0.364***
Slc13a1	solute carrier family 13 (sodium/sulfate symporters), member 1	-1.134	0.000	-0.585	0.001	-0.301	0.017
Ccr10	chemokine (C-C motif) receptor 10	-1.137	0.000	0.036	***0.110***	0.419	0.004
Mzb1	marginal zone B and B1 cell-specific protein	-1.140	0.000	0.064	0.042	0.772	0.000
Ms4a2	membrane-spanning 4-domains, subfamily A, member 2 (Fc fragment of IgE, high affinity I, receptor for; beta polypeptide)	-1.144	0.000	-0.127	***0.555***	0.012	***0.402***
Rup2	urinary protein 2	-1.145	0.009	-1.201	0.000	-0.220	0.030
Rrad	Ras-related associated with diabetes	-1.150	0.000	-0.320	0.036	-0.193	***0.418***
Fbln7	fibulin 7	-1.156	0.000	-0.540	0.001	-0.398	0.009
Bpifb6	BPI fold containing family B, member 6	-1.156	0.000	-0.825	0.000	-0.560	0.000
Cd55	Cd55 molecule	-1.162	0.000	-0.438	0.005	-0.237	0.044
F2	coagulation factor II	-1.165	0.000	-0.640	0.000	-0.315	0.012
Fabp4	fatty acid binding protein 4, adipocyte	-1.170	0.000	-0.233	0.047	-0.334	0.013
Myom2	myomesin 2	-1.171	0.000	-0.772	0.000	-0.257	0.025
RGD1305928	hypothetical LOC300207	-1.180	0.000	-0.851	0.000	-0.388	0.005
Slc18a1	solute carrier family 18 (vesicular monoamine), member 1	-1.184	0.000	-0.791	0.000	-0.599	0.001
Rgs1	regulator of G-protein signaling 1	-1.184	0.000	-0.675	0.001	-0.604	0.001
Dapl1	death associated protein-like 1	-1.196	0.000	-0.753	0.003	-0.375	0.025
Tmem22	transmembrane protein 22	-1.199	0.000	-0.583	0.000	-0.419	0.002
Ccl12	chemokine (C-C motif) ligand 12	-1.212	0.000	0.206	0.010	0.243	0.006

Siglec1	sialic acid binding Ig-like lectin 1, sialoadhesin	-1.213	0.000	0.130	***0.672***	0.102	***0.849***
Fcgr3a	Fc fragment of IgG, low affinity IIIa, receptor	-1.230	0.000	-0.225	***0.934***	0.136	***0.683***
LOC686141	similar to Spetex-2C protein	-1.230	0.000	-0.200	0.006	0.140	0.033
Me1	malic enzyme 1, NADP(+)-dependent, cytosolic	-1.231	0.000	-0.451	0.008	-0.309	0.014
LOC683753	hypothetical protein LOC683753	-1.232	0.004	-0.795	0.034	-0.121	***0.057***
Bhlha15	basic helix-loop-helix family, member a15	-1.233	0.000	-0.496	0.006	-0.067	0.033
LOC688507	similar to Spetex-2F protein	-1.238	0.000	-0.184	0.010	0.110	0.049
H19	H19, imprinted maternally expressed transcript (non-protein coding)	-1.242	0.000	-0.227	***0.052***	-0.317	0.025
Fcer1a	Fc fragment of IgE, high affinity I, receptor for; alpha polypeptide	-1.249	0.000	-0.326	0.032	-0.234	***0.850***
Cxcl11	chemokine (C-X-C motif) ligand 11	-1.260	0.000	-0.859	0.000	-0.267	0.006
Rgs4	regulator of G-protein signaling 4	-1.262	0.000	-0.570	0.003	-0.720	0.000
Ces2c	carboxylesterase 2C	-1.269	0.000	-0.944	0.000	-0.445	0.002
Fut9	fucosyltransferase 9 (alpha (1,3) fucosyltransferase)	-1.270	0.000	-0.997	0.000	-0.334	0.003
Srpk3	SRSF protein kinase 3	-1.272	0.000	-0.242	***0.776***	-0.042	***0.491***
RGD1560949	similar to testis nuclear RNA-binding protein-like	-1.273	0.000	-1.471	0.000	-1.135	0.000
Npw	neuropeptide W	-1.276	0.000	-0.777	0.000	-0.406	0.002
Rbp4	retinol binding protein 4, plasma	-1.283	0.000	-0.026	***0.059***	-0.205	0.015
Olfm4	olfactomedin 4	-1.291	0.000	-0.826	0.000	0.002	***0.082***
Mt3	metallothionein 3	-1.298	0.000	-0.631	0.001	-0.372	0.012
Casq2	calsequestrin 2 (cardiac muscle)	-1.298	0.000	-0.643	0.001	-0.218	***0.835***
Hsd3b5	hydroxy-delta-5-steroid dehydrogenase, 3 beta- and steroid delta-isomerase 5	-1.302	0.000	-0.648	0.000	-0.340	0.001
Gsta5	glutathione S-transferase Yc2 subunit	-1.308	0.000	-0.560	0.001	-0.158	***0.448***
Pln	phospholamban	-1.309	0.000	-0.384	0.001	-0.349	0.010
Nox1	NADPH oxidase 1	-1.313	0.000	-0.256	0.024	0.207	0.023
Me1	malic enzyme 1, NADP(+)-dependent, cytosolic	-1.324	0.000	-0.443	0.009	-0.264	***0.853***
Casc4	cancer susceptibility candidate 4	-1.334	0.000	-0.688	0.000	-0.715	0.000
LOC682360	hypothetical protein LOC682360	-1.335	0.000	-0.459	0.000	-0.314	0.001
Sln	sarcolipin	-1.360	0.000	-0.649	0.001	-0.410	0.004
RGD1563231	similar to immunoglobulin kappa-chain VK-1	-1.370	0.000	-0.170	0.040	-0.580	0.001
Slpi	secretory leukocyte peptidase inhibitor	-1.378	0.000	0.188	***0.186***	0.552	0.006
RGD1562127	similar to chromosome 11 open reading frame 9	-1.382	0.000	-0.687	0.000	-0.360	0.018
Sgcg	sarcoglycan, gamma (dystrophin-associated glycoprotein)	-1.386	0.000	-0.884	0.000	-0.543	0.002
RGD1565970	similar to mast cell protease 8	-1.386	0.000	-0.165	***0.509***	0.310	0.024
LOC685106	similar to ribosomal protein L6	-1.390	0.000	0.215	0.007	-0.613	0.000
Hdc	histidine decarboxylase	-1.393	0.000	-0.281	***0.982***	-0.076	***0.791***
Retnlb	resistin like beta	-1.420	0.000	1.223	0.000	1.939	0.000
RGD1565374	similar to hypothetical protein LOC199675	-1.430	0.000	-0.901	0.000	-0.378	0.010
Apobec2	apolipoprotein B mRNA editing enzyme, catalytic polypeptide-like 2	-1.436	0.000	-0.613	0.001	-0.243	***0.804***
Sypl2	synaptophysin-like 2	-1.457	0.000	-0.801	0.000	-0.867	0.000
Fcer1a	Fc fragment of IgE, high affinity I, receptor for; alpha polypeptide	-1.497	0.000	-0.248	***0.795***	-0.283	***0.824***
Hsd17b6	hydroxysteroid (17-beta) dehydrogenase 6	-1.503	0.000	-0.988	0.000	-0.325	0.001
RGD1565970	similar to mast cell protease 8	-1.509	0.000	-0.198	0.028	0.413	0.009
Tph1	tryptophan hydroxylase 1	-1.518	0.000	-0.642	0.001	-0.305	0.016
Mcpt1l4	mast cell protease 1-like 4	-1.530	0.000	-0.217	***0.840***	0.081	***0.722***
Cfb	complement factor B	-1.551	0.000	0.167	0.013	0.560	0.001
Slfn3	schlafen 3	-1.558	0.000	0.166	***0.551***	0.249	0.018
Retnlg	resistin-like gamma	-1.560	0.000	-0.018	***0.528***	0.509	0.005
LOC679045	Ig gamma chain segment	-1.562	0.000	-0.216	0.004	-0.234	0.001
Cma1	chymase 1, mast cell	-1.583	0.000	-0.288	***0.636***	0.256	0.040
Cpa3	carboxypeptidase A3, mast cell	-1.609	0.000	-0.258	0.028	-0.115	***0.480***
Retnla	resistin like alpha	-1.627	0.000	0.878	0.000	1.162	0.000
Cyp4f1	cytochrome P450, family 4, subfamily f, polypeptide 1	-1.634	0.000	-0.582	0.000	0.169	0.000
Npc1l1	NPC1 (Niemann-Pick disease, type C1, gene)-like 1	-1.662	0.000	-1.294	0.000	-0.707	0.000
Tff1	trefoil factor 1	-1.663	0.000	-0.786	0.000	-0.310	0.001
Atf3	activating transcription factor 3	-1.681	0.000	-1.015	0.000	-1.715	0.000
Fabp1	fatty acid binding protein 1, liver	-1.682	0.000	-0.780	0.000	-0.074	0.001
Tnfrsf17	tumor necrosis factor receptor superfamily, member 17	-1.690	0.000	-0.139	***0.172***	0.572	0.001
Spink4	serine peptidase inhibitor, Kazal type 4	-1.714	0.000	-2.376	0.000	-1.237	0.000

LOC100360169	rCG21044-like	-1.718	0.000	0.232	0.004	1.101	0.000
Ros1	c-ros oncogene 1 , receptor tyrosine kinase	-1.733	0.000	-1.914	0.000	-1.672	0.000
Thrsp	thyroid hormone responsive	-1.747	0.000	-0.067	0.004	-0.380	0.001
RatNP-3b	defensin RatNP-3 precursor	-1.761	0.000	-2.005	0.000	-0.576	0.002
RGD1564563	similar to SIGNR4	-1.793	0.000	-0.383	0.007	-0.092	***0.672***
Mcpt8	mast cell protease 8	-1.824	0.000	-0.324	0.010	0.315	0.023
Mcpt8l3	mast cell protease 8-like 3	-1.824	0.000	-0.462	0.004	0.151	***0.564***
Adipoq	adiponectin, C1Q and collagen domain containing	-1.826	0.000	0.053	0.007	-0.265	0.002
Mcpt9	mast cell protease 9	-1.834	0.000	-0.384	0.006	0.240	***0.057***
Mcpt4	mast cell protease 4	-1.952	0.000	0.233	0.025	0.296	0.039
Mcpt1	mast cell protease 1	-1.966	0.000	-0.014	***0.058***	0.285	0.018
Mcpt10	mast cell protease 10	-1.985	0.000	-0.523	0.002	0.192	***0.727***
LOC100359793	mCG1050586-like	-1.987	0.000	-0.141	0.019	1.137	0.000
Scd1	stearoyl-Coenzyme A desaturase 1	-1.998	0.000	0.138	0.002	-0.400	0.000
Mcpt8l2	mast cell protease 8-like 2	-2.003	0.000	-0.532	0.002	0.196	***0.877***
Pga5	pepsinogen 5, group I	-2.086	0.000	-1.394	0.000	-0.775	0.000
Gkn2	gastrokine 2	-2.099	0.000	-1.000	0.000	-1.584	0.000
Mcpt1l4	mast cell protease 1-like 4	-2.170	0.000	-0.112	0.049	0.239	0.034
RGD1562035	similar to mast cell protease 1-like 3 precursor	-2.179	0.000	-0.248	0.020	0.176	***0.624***
Pzp	pregnancy-zone protein	-2.223	0.000	-1.221	0.000	-1.117	0.000
Fcrls	Fc receptor-like S, scavenger receptor	-2.342	0.000	-0.529	0.001	0.208	0.018
Bpifb2	BPI fold containing family B, member 2	-2.508	0.000	-1.532	0.000	-1.300	0.000
Mcpt2	mast cell protease 2	-2.545	0.000	-0.290	0.030	0.255	***0.053***
Tac4	tachykinin 4	-2.766	0.000	-1.731	0.000	-1.279	0.000
