# Supplementary material for: Cocoa polyphenols and fiber modify colonic gene expression in rats
Source: Eur J Nutr. 2016 Jun 2;56(5):1871–85. doi: 10.1007/s00394-016-1230-0 (PMC5534200; doi:10.1007/s00394-016-1230-0)
Supplement: Supplementary file 2 — Supplementary material 2 (DOC 107 kb) [file 394_2016_1230_MOESM2_ESM.doc]

|  |  | **C10 vs REF** | | |  | **CF vs REF** | | **I vs REF** | | |
| --- | --- | --- | --- | --- | --- | --- | --- | --- | --- | --- |
|  | **GO TERM** | **Exp Counts** | **Counts** | **P-Value** | **Exp Counts** | **Counts** | **P-Value** | **Exp Counts** | **Counts** | **P-Value** |
| **Biological Process** | chemical homeostasis (GO:0048878) | 3 | 12 | 1.57E-05 | 1 | 6 | 1.04E-03 |  |  |  |
|  | response to inorganic substance (GO:0010035) | 2 | 9 | 2.68E-05 | 1 | 7 | 3.62E-06 |  |  |  |
|  | detoxification of copper ion (GO:0010273) | 0 | 2 | 3.60E-05 |  |  |  |  |  |  |
|  | ion homeostasis (GO:0050801) | 2 | 10 | 5.03E-05 | 1 | 5 | 2.17E-03 |  |  |  |
|  | oxygen transport (GO:0015671) | 0 | 2 | 7.19E-05 | 0 | 2 | 1.34E-05 |  |  |  |
|  | homeostatic process (GO:0042592) | 4 | 13 | 9.04E-05 | 2 | 7 | 1.02E-03 |  |  |  |
|  | cellular response to zinc ion (GO:0071294) | 0 | 2 | 1.20E-04 |  |  |  |  |  |  |
|  | prostate glandular acinus morphogenesis (GO:0060526) | 0 | 2 | 2.50E-04 | 0 | 1 | 1.07E-02 |  |  |  |
|  | prostate epithelial cord arborization involved in prostate glandular acinus morphogenesis (GO:0060527) | 0 | 2 | 2.50E-04 | 0 | 1 | 1.07E-02 |  |  |  |
|  | regulation of cell-cell adhesion mediated by integrin (GO:0033632) | 0 | 2 | 3.32E-04 | 0 | 1 | 1.22E-02 |  |  |  |
|  | gas transport (GO:0015669) | 0 | 2 | 5.32E-04 | 0 | 3 | 1.00E-04 |  |  |  |
|  | response to stress (GO:0006950) | 8 | 15 | 5.66E-03 | 0 | 11 | 1.14E-04 |  |  |  |
|  | hydrogen peroxide catabolic process (GO:0042744) | 0 | 2 | 2.20E-03 | 0 | 2 | 4.19E-04 |  |  |  |
|  | protein heterooligomerization (GO:0051291) | 0 | 3 | 5.75E-03 | 0 | 3 | 5.13E-04 |  |  |  |
|  | response to hypoxia (GO:0001666) | 1 | 5 | 3.40E-03 | 0 | 4 | 8.99E-04 |  |  |  |
|  | response to decreased oxygen levels (GO:0036293) | 1 | 5 | 3.55E-03 | 0 | 4 | 9.34E-04 |  |  |  |
| **Cellular Component** | hemoglobin complex (GO:0005833) | 0 | 5 | 2.07E-10 | 0 | 4 | 2.94E-09 |  |  |  |
|  | haptoglobin-hemoglobin complex (GO:0031838) | 0 | 2 | 7.31E-05 | 0 | 2 | 1.94E-05 |  |  |  |
|  | extracellular region (GO:0005576) | 5 | 15 | 7.50E-05 | 3 | 10 | 1.20E-04 | 1 | 4 | 2.01E-03 |
|  | cytosolic part (GO:0044445) | 1 | 5 | 4.58E-04 | 0 | 5 | 1.83E-05 |  |  |  |
|  | rough endoplasmic reticulum (GO:0005791) | 0 | 3 | 1.18E-03 |  |  |  |  |  |  |
|  | 4-aminobutyrate transaminase complex (GO:0032144) | 0 | 1 | 3.53E-03 | 0 | 1 | 1.83E-03 |  |  |  |
|  | subapical complex (GO:0035003) | 0 | 1 | 3.53E-03 |  |  |  |  |  |  |
|  | apical part of cell (GO:0045177) | 1 | 5 | 4.41E-03 | 1 | 3 | 1.80E-02 |  |  |  |
|  | intracellular canaliculus (GO:0046691) | 0 | 1 | 7.05E-03 |  |  |  |  |  |  |
|  | invadopodium membrane (GO:0071438) | 0 | 1 | 7.05E-03 |  |  |  |  |  |  |
|  | phosphopyruvate hydratase complex (GO:0000015) |  |  |  | 0 | 1 | 7.31E-03 |  |  |  |
|  | apical plasma membrane (GO:0016324) | 1 | 4 | 8.77E-03 | 0 | 3 | 8.34E-03 |  |  |  |
|  | sodium channel complex (GO:0034706) |  |  |  | 0 | 1 | 2.89E-02 |  |  |  |
|  | Aggresome (GO:0016235) |  |  |  | 0 | 1 | 3.25E-02 |  |  |  |
| **Molecular Function** | oxygen binding (GO:0019825) | 0 | 5 | 1.09E-08 | 0 | 4 | 6.70E-08 |  |  |  |
|  | oxygen transporter activity (GO:0005344) | 0 | 4 | 5.31E-08 | 0 | 3 | 1.08E-06 |  |  |  |
|  | heme binding (GO:0020037) | 1 | 8 | 8.44E-08 | 0 | 6 | 4.22E-07 |  |  |  |
|  | tetrapyrrole binding (GO:0046906) | 1 | 8 | 1.14E-07 | 0 | 6 | 5.29E-07 |  |  |  |
|  | iron ion binding (GO:0005506) | 1 | 9 | 2.39E-07 | 0 | 6 | 6.30E-06 |  |  |  |
|  | haptoglobin binding (GO:0031720) | 0 | 2 | 3.98E-05 | 0 | 2 | 1.09E-05 |  |  |  |
|  | hemoglobin alpha binding (GO:0031721) | 0 | 2 | 3.98E-05 | 0 | 2 | 1.09E-05 |  |  |  |
|  | CCR10 chemokine receptor binding (GO:0031735) | 0 | 2 | 3.98E-05 | 0 | 2 | 1.09E-05 | 0 | 1 | 1.67E-03 |
|  | substrate-specific transporter activity (GO:0022892) | 3 | 12 | 6.57E-05 | 2 | 5 | 1.96E-04 |  |  |  |
|  | hemoglobin binding (GO:0030492) | 0 | 2 | 2.76E-04 | 0 | 2 | 7.62E-05 |  |  |  |

**Table S2** Summary of the most enriched over-represented GO terms in up-regulated genes belonging to biological process (BP), cellular component (CC) and molecular function (MF), in the C10 and FC groups in comparison with the REF group, adding those shared with the I diet. The number of the expected genes, the counts as well as the *P*-value for each GO term are included (n=4/group)
