# Supplementary material for: Cocoa polyphenols and fiber modify colonic gene expression in rats
Source: Eur J Nutr. 2016 Jun 2;56(5):1871–85. doi: 10.1007/s00394-016-1230-0 (PMC5534200; doi:10.1007/s00394-016-1230-0)
Supplement: Supplementary file 3 — Supplementary material 3 (DOC 133 kb) [file 394_2016_1230_MOESM3_ESM.doc]

| **Table S3** Summary of the most enriched over-represented GO terms in down-regulated genes belonging to biological process (BP), cellular component (CC) and molecular function (MF) in the C10 and FC groups in comparison with the REF group, adding those shared with the I diet. The number of the expected genes, the counts as well as the *P-*value for each GO term are included (n=4/group) |  | **C10 vs REF** | | |  | **CF vs REF** | | **I vs REF** | | |
| --- | --- | --- | --- | --- | --- | --- | --- | --- | --- | --- |
|  | **GO TERM** | **Exp Counts** | **Counts** | **P-Value** | **Exp Counts** | **Counts** | **P-Value** | **Exp Counts** | **Counts** | **P-Value** |
| **Biological Process** | digestive system process (GO:0022600) | 1 | 8 | 5.43E-08 | 0 | 6 | 8.69E-09 | 0 | 3 | 1.10E-04 |
|  | cellular cation homeostasis (GO:0030003) | 3 | 15 | 3.81E-07 |  |  |  |  |  |  |
|  | digestion (GO:0007586) | 1 | 8 | 6.67E-07 | 0 | 6 | 6.00E-08 | 0 | 3 | 2.83E-04 |
|  | chemical homeostasis (GO:0048878) | 7 | 23 | 8.08E-07 |  |  |  |  |  |  |
|  | regulation of biological quality (GO:0065008) | 19 | 40 | 1.85E-06 | 6 | 12 | 5.75E-03 |  |  |  |
|  | response to organic substance (GO:0010033) | 16 | 35 | 2.10E-06 |  |  |  | 3 | 8 | 3.74E-03 |
|  | cation homeostasis (GO:0055080) | 4 | 15 | 2.38E-06 |  |  |  |  |  |  |
|  | regulation of calcium ion transport into cytosol (GO:0010522) | 0 | 6 | 2.98E-06 |  |  |  |  |  |  |
|  | cellular metal ion homeostasis (GO:0006875) | 3 | 13 | 3.22E-06 |  |  |  |  |  |  |
|  | cytosolic calcium ion transport (GO:0060401) | 1 | 7 | 3.81E-06 |  |  |  |  |  |  |
|  | intestinal cholesterol absorption (GO:0030299) | 0 | 2 | 2.81E-03 | 0 | 3 | 1.39E-06 | 0 | 2 | 8.81E-05 |
|  | lipid digestion (GO:0044241) | 0 | 2 | 4.24E-03 | 0 | 3 | 2.71E-06 | 0 | 2 | 1.34E-04 |
|  | intestinal absorption (GO:0050892) |  |  |  | 0 | 3 | 1.33E-05 | 0 | 2 | 3.71E-04 |
|  | steroid metabolic process (GO:0008202) | 2 | 9 | 2.13E-04 | 1 | 6 | 2.44E-05 |  |  |  |
|  | regulation of digestive system process (GO:0044058) |  |  |  | 0 | 3 | 4.16E-05 | 0 | 3 | 9.14E-06 |
|  | positive regulation of cholesterol esterification (GO:0010873) |  |  |  | 0 | 2 | 1.00E-04 | 0 | 2 | 3.68E-05 |
|  | regulation of intestinal cholesterol absorption (GO:0030300) |  |  |  | 0 | 2 | 1.00E-04 | 0 | 2 | 3.68E-05 |
|  | cellular hormone metabolic process (GO:0034754) |  |  |  | 0 | 4 | 1.17E-04 |  |  |  |
| **Cellular Component** | extracellular region (GO:0005576) | 12 | 44 | 5.78E-15 | 4 | 16 | 1.22E-07 | 2 | 12 | 1.86E-07 |
|  | extracellular space (GO:0005615) | 6 | 26 | 2.03E-10 | 2 | 9 | 5.58E-05 | 1 | 8 | 5.31E-06 |
|  | extracellular region part (GO:0044421) | 8 | 27 | 5.42E-09 | 2 | 10 | 5.59E-05 | 1 | 9 | 2.79E-06 |
|  | sarcoplasmic reticulum (GO:0016529) | 0 | 5 | 2.72E-05 |  |  |  |  |  |  |
|  | sarcoplasm (GO:0016528) | 0 | 5 | 4.74E-05 |  |  |  |  |  |  |
|  | sarcoplasmic reticulum lumen (GO:0033018) | 0 | 2 | 4.26E-04 |  |  |  |  |  |  |
|  | endoplasmic reticulum part (GO:0044432) | 5 | 13 | 1.14E-03 |  |  |  |  |  |  |
|  | myofibril (GO:0030016) | 1 | 6 | 1.39E-03 |  |  |  |  |  |  |
|  | contractile fiber (GO:0043292) | 1 | 6 | 1.90E-03 |  |  |  |  |  |  |
|  | extracellular matrix (GO:0031012) | 3 | 9 | 3.03E-03 |  |  |  | 1 | 3 | 1.50E-02 |
|  | very-low-density lipoprotein particle (GO:0034361) | 0 | 2 | 8.98E-03 | 0 | 2 | 8.42E-04 | 0 | 2 | 2.90E-04 |
|  | triglyceride-rich lipoprotein particle (GO:0034385) | 0 | 2 | 8.98E-03 | 0 | 2 | 8.42E-04 | 0 | 2 | 2.90E-04 |
|  | high-density lipoprotein particle (GO:0034364) |  |  |  | 0 | 2 | 1.06E-03 | 0 | 2 | 3.65E-04 |
|  | plasma lipoprotein particle (GO:0034358) | 0 | 2 | 2.19E-02 | 0 | 2 | 2.14E-03 | 0 | 2 | 7.43E-04 |
|  | protein-lipid complex (GO:0032994) | 0 | 2 | 2.35E-02 | 0 | 2 | 2.30E-03 | 0 | 2 | 7.99E-04 |
|  | endocytic vesicle (GO:0030139) |  |  |  | 0 | 2 | 1.34E-02 |  |  |  |
|  | meiotic cohesin complex (GO:0030893) | 0 | 1 | 5.00E-02 | 0 | 1 | 1.52E-02 |  |  |  |
| **Molecular Function** | serine-type endopeptidase activity (GO:0004252) | 1 | 9 | 1.07E-05 |  |  |  |  |  |  |
|  | enzyme inhibitor activity (GO:0004857) | 2 | 11 | 2.28E-05 | 1 | 5 | 1.03E-03 | 0 | 5 | 4.87E-05 |
|  | serine-type peptidase activity (GO:0008236) | 2 | 9 | 2.71E-05 |  |  |  |  |  |  |
|  | serine hydrolase activity (GO:0017171) | 2 | 9 | 3.08E-05 |  |  |  |  |  |  |
|  | peptidase inhibitor activity (GO:0030414) | 1 | 8 | 8.85E-05 | 0 | 4 | 9.38E-04 | 0 | 4 | 1.06E-04 |
|  | carbonate dehydratase activity (GO:0004089) | 0 | 3 | 9.56E-05 |  |  |  |  |  |  |
|  | endopeptidase activity (GO:0004175) | 3 | 11 | 1.85E-04 |  |  |  |  |  |  |
|  | immunoglobulin binding (GO:0019865) | 0 | 3 | 2.07E-04 |  |  |  |  |  |  |
|  | IgE receptor activity (GO:0019767) | 0 | 2 | 2.16E-04 |  |  |  |  |  |  |
|  | ATPase inhibitor activity (GO:0042030) | 0 | 2 | 2.16E-04 |  |  |  |  |  |  |
|  | steroid dehydrogenase activity, acting on the CH-OH group of donors, NAD or NADP as acceptor (GO:0033764) |  |  |  | 0 | 3 | 4.73E-05 |  |  |  |
|  | phosphatidylcholine-sterol O-acyltransferase activator activity (GO:0060228) |  |  |  | 0 | 2 | 4.73E-05 | 0 | 2 | 1.34E-05 |
|  | steroid dehydrogenase activity (GO:0016229) |  |  |  | 0 | 3 | 9.13E-05 |  |  |  |
|  | 3-beta-hydroxy-delta5-steroid dehydrogenase activity (GO:0003854) | 0 | 2 | 1.48E-03 | 0 | 2 | 1.65E-04 |  |  |  |
|  | sterol transporter activity (GO:0015248) |  |  |  | 0 | 2 | 4.28E-04 | 0 | 2 | 1.22E-04 |
|  | cholesterol transporter activity (GO:0017127) |  |  |  | 0 | 2 | 4.28E-04 | 0 | 2 | 1.22E-04 |
|  | endopeptidase inhibitor activity (GO:0004866) |  |  |  | 0 | 4 | 9.38E-04 | 0 | 4 | 7.94E-05 |
|  | endopeptidase regulator activity (GO:0061135) | 1 | 6 | 2.59E-03 | 0 | 4 | 1.11E-03 | 0 | 4 | 9.43E-05 |
